# PI3K drives the de novo synthesis of coenzyme A from vitamin B5

**DOI:** 10.1038/s41586-022-04984-8

**Published:** 2022-07-27

**Authors:** Christian C. Dibble, Samuel A. Barritt, Grace E. Perry, Evan C. Lien, Renee C. Geck, Sarah E. DuBois-Coyne, David Bartee, Thomas T. Zengeya, Emily B. Cohen, Min Yuan, Benjamin D. Hopkins, Jordan L. Meier, John G. Clohessy, John M. Asara, Lewis C. Cantley, Alex Toker

**Affiliations:** 1 Department of Pathology, Cancer Research Institute, Beth Israel Deaconess Medical Center, Harvard Medical School, Boston, MA USA; 2 Chemical Biology Laboratory, National Cancer Institute, Frederick, MD USA; 3 Mass Spectrometry Core, Department of Medicine, Beth Israel Deaconess Medical Center, Harvard Medical School, Boston, MA USA; 4 Department of Genetics and Genomic Sciences, Tisch Cancer Institute, Icahn School of Medicine at Mount Sinai, New York, NY USA; 5 Preclinical Murine Pharmacogenetics Facility and Mouse Hospital, Department of Medicine, Beth Israel Deaconess Medical Center, Boston, MA USA; 6 The Sandra and Edward Meyer Cancer Center, Weill Medical College of Cornell University, New York, NY USA; 7 Ludwig Center at Harvard, Harvard Medical School, Boston, MA USA

**Keywords:** Metabolomics, Cancer metabolism

## Abstract

In response to hormones and growth factors, the class I phosphoinositide-3-kinase (PI3K) signalling network functions as a major regulator of metabolism and growth, governing cellular nutrient uptake, energy generation, reducing cofactor production and macromolecule biosynthesis^1^. Many of the driver mutations in cancer with the highest recurrence, including in receptor tyrosine kinases, Ras, PTEN and PI3K, pathologically activate PI3K signalling^2,3^. However, our understanding of the core metabolic program controlled by PI3K is almost certainly incomplete. Here, using mass-spectrometry-based metabolomics and isotope tracing, we show that PI3K signalling stimulates the de novo synthesis of one of the most pivotal metabolic cofactors: coenzyme A (CoA). CoA is the major carrier of activated acyl groups in cells^4,5^ and is synthesized from cysteine, ATP and the essential nutrient vitamin B5 (also known as pantothenate)^6,7^. We identify pantothenate kinase 2 (PANK2) and PANK4 as substrates of the PI3K effector kinase AKT^8^. Although PANK2 is known to catalyse the rate-determining first step of CoA synthesis, we find that the minimally characterized but highly conserved PANK4^9^ is a rate-limiting suppressor of CoA synthesis through its metabolite phosphatase activity. Phosphorylation of PANK4 by AKT relieves this suppression. Ultimately, the PI3K–PANK4 axis regulates the abundance of acetyl-CoA and other acyl-CoAs, CoA-dependent processes such as lipid metabolism and proliferation. We propose that these regulatory mechanisms coordinate cellular CoA supplies with the demands of hormone/growth-factor-driven or oncogene-driven metabolism and growth.

## Abstract (web summary)

The PI3K–PANK4 axis regulates coenzyme A synthesis, the abundance of acetyl-CoA, and CoA-dependent processes such as lipid metabolism, and these regulatory mechanisms coordinate cellular CoA supplies with the demands of hormone and growth-factor-driven or oncogene-driven metabolism and growth.

## Main

**Fig. 1:**
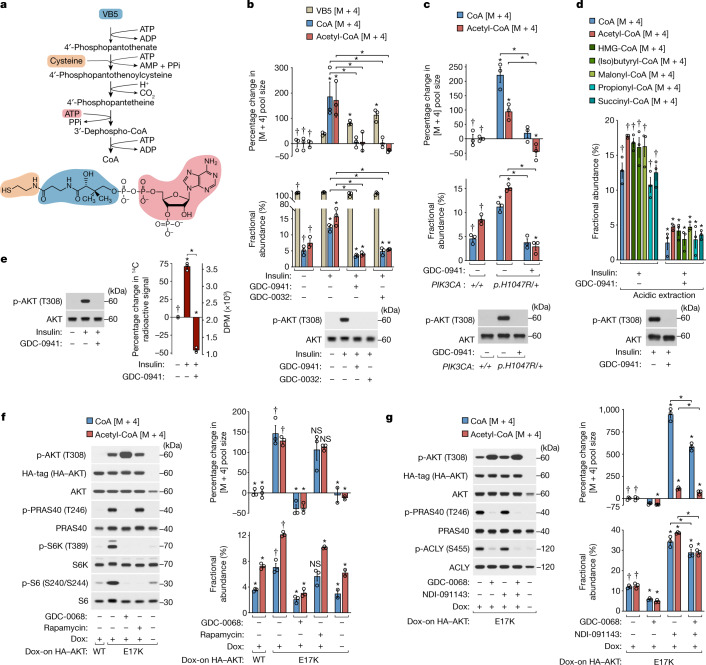
PI3K–AKT signalling stimulates de novo CoA synthesis. **a**, The CoA de novo synthesis pathway. **b**, Insulin (100 nM) and PI3K inhibitor (GDC-0941 or GDC-0032; 2 μM) treatments with concurrent ^13^C_3_^15^N_1_-VB5 labelling (3 h), preceded by serum/growth factor deprivation (18 h) and inhibitor pretreatment (15 min) in MCF10A cells. **c**, *PIK3CA*^+/+^ and *PIK3CA*^*p.H1047R/+*^-knockin MCF10A cells with PI3K inhibitor (GDC-0941; 2 μM) treatment. Labelling and conditions were otherwise as described in **b**. **d**, Acid-extracted CoA and short-chain acyl-CoAs with the cells and conditions as described in **b**, except with 4 h treatments and labelling. **e**, Radioactive ^14^C-VB5 labelling (3 h) with the cells and conditions otherwise as described in **b**, followed by a chase (1 h) in medium without VB5. Disintegrations per minute (DPM) normalized to protein. **f**, AKT inhibitor (GDC-0068, 2 μΜ) and mTORC1 inhibitor (rapamycin, 100 nM) treatments with concurrent ^13^C_3_^15^N_1_-VB5 labelling (3 h) of MCF10A cells expressing doxycycline (Dox)-inducible HA-tagged wild-type (WT) or constitutively active (E17K) AKT. Treatments and labelling were preceded by doxycycline incubation (48 h), serum and growth factor deprivation (18 h) and inhibitor pretreatment (15 min). **g**, AKT inhibitor (GDC-0068, 2 μΜ) and ACLY inhibitor (NDI-091143, 15 μM) treatments with the cells, labelling and conditions otherwise as described in **f**. For **b–d**, **f** and **g**, metabolites were measured using LC–MS/MS and normalized to protein; labelled metabolites (mass + 4 [M + 4]); fractional abundance is [M + 4]/total. For the percentage change graphs, the left-most treatment group mean was set to 0%. For **b**–**g**, *n* = 3 biological replicates (circles). Data are mean ± s.e.m. Statistical analysis was performed using one-way analysis of variance (ANOVA) with Tukey test; asterisks (*) indicate significant differences compared with the treatment groups marked with daggers (†) or between treatments indicated with brackets (*P* *<* 0.05). Immunoblotting analysis probed for total and phosphorylated (p) proteins.

**Extended Data Fig. 1:**
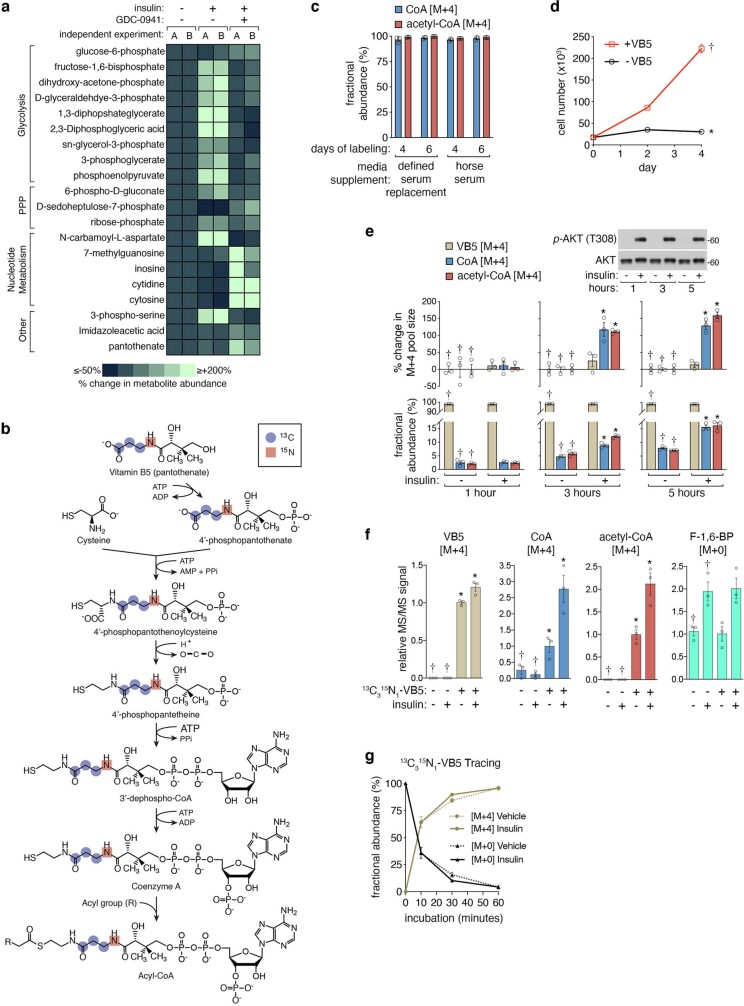
Data supporting Fig. 1. **a**, PI3K-regulated metabolite screen. Serum/growth factor-deprived (18 h) MCF10A cells treated (1 h) with insulin (100 nM) and PI3K inhibitor (GDC-0941, 2 μM). Graphed metabolites were significantly (one-way ANOVA, p<alpha 0.05) different by ≥ 50% between any two treatments in both independent experiments (A and B). **b**, Diagram of heavy isotope (^13^C_3_^15^N_1_)-labelled Vitamin B5 (VB5) tracing into CoA and acyl-CoAs. Labelled carbon (blue), nitrogen (red). Labelled VB5, CoA, and acyl-CoAs are all native mass + 4 [M+4]. **c**, Steady state ^13^C_3_^15^N_1_-VB5 labelling of MCF10A cells (4–6 days) in media containing only ^13^C_3_^15^N_1_-VB5, supplemented with either defined serum replacement or horse serum. Metabolites measured by LC-MS/MS and displayed as fractional abundance of labelled metabolites ((M+4)/total). Replicate samples (○); mean (bars); SEM (error bars); n = 3 biological replicates. **d**, Growth of MCF10A cells over 4 days in serum-free media containing growth factors and defined serum replacement, in the presence or absence of 1 µM VB5. Cells were grown in the absence of VB5 for 48 h prior to plating. Mean (points on line); SEM (error bars); n = 3 biological replicates. *significant difference from treatment marked (†), two-sided student’s t-test, (p<alpha 0.05). **e**, Time course of MCF10A cells serum/growth factor-deprived (18 h), pre-incubated (1 h) with ^13^C_3_^15^N_1_-VB5, and treated (1, 3, or 5 h) with insulin (100 nM) and ^13^C_3_^15^N_1_-VB5 labelling. Protein immunoblots probed with antibodies to total and phosphorylated (*p*) AKT (left); molecular weight markers in kD (right). Graphed metabolites measured by LC-MS/MS. Replicate samples (○); mean (bars); SEM (error bars); n = 3 biological replicates. *significant difference from treatment marked (†), two-way ANOVA with Sidak’s test, (p<alpha 0.05). **f**, Validation of MS/MS methods for ^13^C_3_^15^N_1_-VB5 tracing into VB5, CoA, and acetyl-CoA. Serum/growth factor-deprived (18 h) MCF10A cells were treated (3 h) with insulin (100 nM) and labelled with ^13^C_3_^15^N_1_-VB5. Unlabelled fructose-1,6-bisphosphate (F-1,6-BP) shown as control. Graphed metabolites measured by LC-MS/MS. Replicate samples (○); mean (bars); SEM (error bars); n = 3 biological replicates. *significant difference from treatment marked (†), one-way ANOVA with Tukey’s test, (p<alpha 0.05). **g**, Time course showing intracellular levels of ^13^C_3_^15^N_1_-VB5 in serum/growth factor deprived (18 h) MCF10A cells upon incubation in media exclusively containing ^13^C_3_^15^N_1_-VB5 for 10–60 min. Graphed metabolites measured my LC-MS/MS. Mean (points on line); SEM (error bars); n = 3 biological replicates.

In a search for core components of the class I PI3K-driven metabolic program, we performed an unlabelled, targeted liquid chromatography–tandem mass spectrometry (LC–MS/MS)-based metabolomics screen using the non-transformed human breast epithelial cell line MCF10A. PI3K has important roles in mammary gland growth and is a major driver of breast cancer^10,11^. MCF10A cells are similarly dependent on growth-factor-stimulated PI3K signalling for growth and proliferation. Cells were treated with insulin to acutely stimulate PI3K signalling in the presence or absence of a PI3K inhibitor (Extended Data Fig. 1a). In addition to inducing changes in known PI3K-regulated metabolic pathways^1^, we observed that PI3K inhibition increased the concentration of pantothenate, an essential nutrient that is also known as vitamin B5 (VB5). VB5 is uniquely used in combination with cysteine and ATP for de novo biosynthesis of the cofactor CoA^6,7^ (Fig. 1a). As the major carrier of activated acyl groups within cells, CoA has a critical role in a variety of core metabolic processes, including nutrient catabolism and lipid synthesis^4,5^.

## PI3K and AKT stimulate CoA synthesis

**Extended Data Fig. 2:**
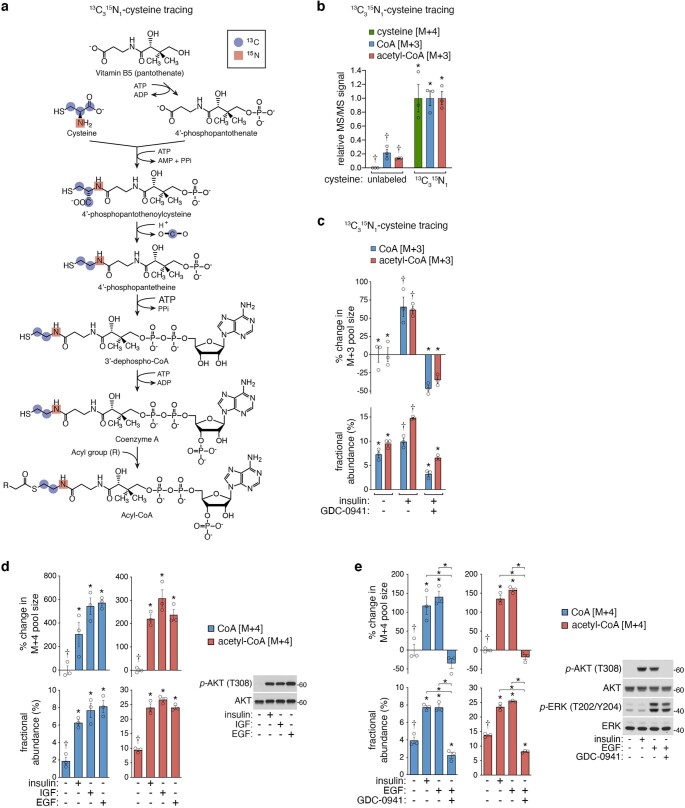
Data supporting Fig. 1. **a**, Diagram of heavy isotope (^13^C_3_^15^N_1_)-labelled cysteine tracing into CoA and acyl-CoAs. Labelled carbon (blue) and nitrogen (red). Note that labelled cysteine is M+4 but that labelled CoA and acyl-CoAs are M+3 due to the decarboxylation step of CoA synthesis. **b**, Validation of MS/MS methods for ^13^C_3_^15^N_1_-cysteine tracing. Serum/growth factor-deprived (18 h) MCF10A cells were treated (3 h) with insulin (100 nM) and concurrently labelled with ^13^C_3_^15^N_1_-cysteine. Graphed metabolites measured by LC-MS/MS. Replicate samples (○); mean (bars); SEM (error bars); n = 3 biological replicates. *significant difference from treatment marked (†), two-way ANOVA with Sidak’s test, (p<alpha 0.05). **c**, ^13^C_3_^15^N_1_-cysteine tracing (3 h) with concurrent insulin (100 nM) and PI3K inhibitor (GDC-0941, 2 μM) treatments in MCF10A cells. Cells were serum/growth factor-deprived (18 h) and pretreated with inhibitor prior to tracing. Graphed metabolites measured by LC-MS/MS. Replicate samples (○); mean (bars); SEM (error bars); n = 3 biological replicates. *significant difference from treatment marked (†), one-way ANOVA with Tukey’s test, (p<alpha 0.05). **d**, Insulin (100 nM), Epidermal Growth Factor (EGF, 50 ng ml^−1^), Insulin-like growth factor 1 (IGF-1, 50 ng ml^−1^) treatments with concurrent ^13^C_3_^15^N_1_-VB5 labelling (3 h) of serum/growth factor-deprived (18 h) MCF10A cells. Protein immunoblots probed with indicated antibodies (left); molecular weight markers in kD (right). Graphed metabolites measured by LC-MS/MS. Replicate samples (○); mean (bars); SEM (error bars). *significant difference from treatment marked (†), one-way ANOVA with Tukey’s test, (p<alpha 0.05). **e**, Insulin (100 nM), Epidermal Growth Factor (EGF, 50 ng ml^−1^), PI3K inhibitor (GDC-0941, 2 µM) treatments with concurrent ^13^C_3_^15^N_1_-VB5 labelling (3 h) of serum/growth factor-deprived (18 h) MCF10A cells, pretreated with inhibitor prior to tracing. Protein immunoblots probed with indicated antibodies (left); molecular weight markers in kD (right). Graphed metabolites measured by LC-MS/MS. Replicate samples (○); mean (bars); SEM (error bars); n = 3 biological replicates. *significant difference from treatment marked (†), one-way ANOVA with Tukey’s test, (p<alpha 0.05).

Early studies of CoA synthesis in mammals reported effects of hormones on CoA abundance, but the molecular regulatory mechanisms underlying these responses remain undefined^6,7^. To investigate the connection between PI3K, VB5 and CoA, we monitored the incorporation of heavy isotope (^13^C_3_^15^N_1_)-labelled VB5 into free unacylated CoA, acetyl-CoA and other acyl-CoAs using targeted LC–MS/MS^12,13^ (Extended Data Fig. 1b). Under steady-state labelling conditions, VB5, CoA and acetyl-CoA pools approached 100% fractional labelling, whether the medium included horse serum or a defined serum replacement lacking unlabelled VB5 (Extended Data Fig. 1c). Consistent with this result and its role as an essential nutrient, VB5 was required for cell proliferation (Extended Data Fig. 1d). Thus, under the conditions used to culture MCF10A cells, nearly all CoA is ultimately derived from VB5 supplied in the medium. In cells deprived of serum and growth factors (to minimize PI3K signalling) and incubated with ^13^C_3_^15^N_1_-VB5, insulin activated PI3K as indicated by AKT phosphorylation^8^ and substantially increased the abundance of newly synthesized (labelled) CoA and acetyl-CoA after 3–5 h (Extended Data Fig. 1e; MS/MS methods validated in Extended Data Fig. 1f). The fraction of labelled VB5 approached 100% within an hour and insulin had little effect on the initial rate of VB5 uptake, although VB5 slightly accumulated with insulin treatment at later time points (Extended Data Fig. 1e,g). Pharmacological inhibition of PI3K completely blocked the insulin-induced increase in labelled CoA and acetyl-CoA abundance, while increasing labelled VB5 abundance (Fig. 1b). A complementary tracing strategy using heavy-isotope-labelled ^13^C_3_^15^N_1_-cysteine yielded similar results (Extended Data Fig. 2a–c). The growth factors IGF-1 and EGF also stimulated an increase in labelled CoA and acetyl-CoA abundance and, in EGF-stimulated cells, PI3K inhibition blocked this increase without affecting MEK–ERK activation (Extended Data Fig. 2d,e). In the absence of growth factors, expression of a cancer-derived, constitutively active point mutant (H1047R) of PI3Kα was sufficient to increase labelled CoA and acetyl-CoA abundance (Fig. 1c). Thus, PI3K signalling stimulates an increase in the abundance of CoA and acetyl-CoA synthesized de novo from VB5.

**Extended Data Fig. 3:**
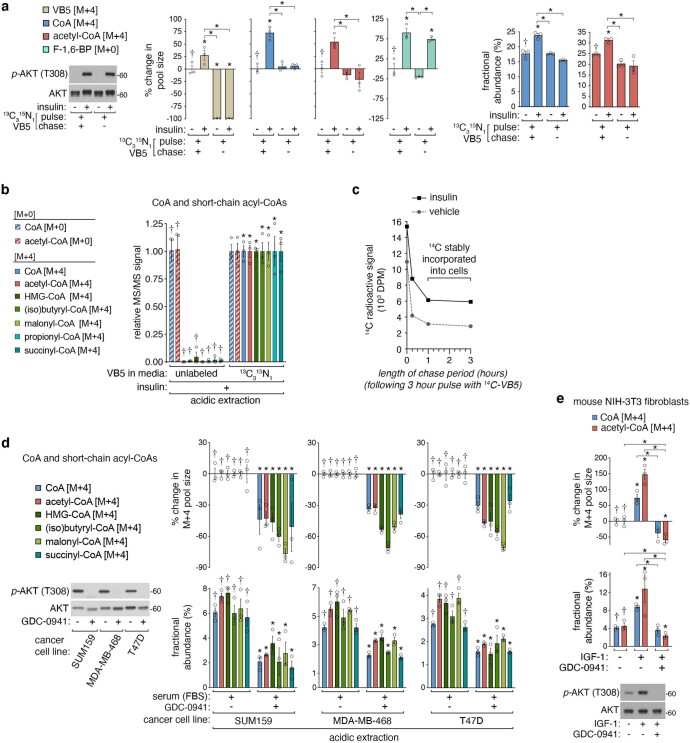
Data supporting Fig. 1. **a**, Pulse-chase labelling of insulin-stimulated MCF10A cells. Cells were serum/growth factor-deprived (18 h), pulse-labelled (4 h) with ^13^C_3_^15^N_1_-VB5, incubated in media without VB5 (1 h), and then chased (3 h) in media with or without ^13^C_3_^15^N_1_-VB5 and insulin (100 nM). Protein immunoblots probed with indicated antibodies (left); molecular size makers in kD (right). Graphed metabolites measured by LC-MS/MS. Replicate samples (○); mean (bars); SEM (error bars); n = 3 biological replicates. *significant difference from treatment marked (†), one-way ANOVA with Tukey’s test, (p<alpha 0.05). **b**, Validation of LC-MS/MS methods for ^13^C_3_^15^N_1_-VB5 tracing into short-chain acyl-CoAs. Serum/growth factor-deprived (18 h) MCF10A cells were treated (4 h) with insulin (100 nM) and ^13^C_3_^15^N_1_-VB5. Metabolites were extracted under acidic conditions. Unlabelled (M+0) CoA and acetyl-CoA levels are shown for reference. Replicate samples (○); mean (bars); SEM (error bars); n = 3 biological replicates. *significant difference from treatment marked (†), one-way ANOVA with Tukey’s test, (p<alpha 0.05). **c**, Intracellular radioactivity counts during chase period after a pulse with radioactive ^14^C-VB5. Serum/growth factor-deprived (18 h) MCF10A cells were pulsed (3 h) with radioactive ^14^C-VB5 and insulin (100 nM) and then chased in media lacking ^14^C-VB5 for 15 min, 1 h, and 3 h to wash out unincorporated ^14^C-VB5. Intracellular levels of radioactivity at each timepoint were measured using scintillation counting of disintegrations per minute (DPM). **d**, PI3K inhibitor (GDC-0941, 2 μΜ) treatment of breast cancer cell lines SUM159, MDA-MB-468, and T47D grown in serum-containing media with concurrent ^13^C_3_^15^N_1_-VB5 labelling (3 h). Protein immunoblots probed with indicated antibodies (left); molecular weight markers (right). Graphed metabolites measured by LC-MS/MS. Replicate samples (○); mean (bars); SEM (error bars); n = 3 biological replicates. *significant difference from treatment marked (†), two-way ANOVA with Sidak’s test, (p<alpha 0.05). **e**, Mouse NIH-3T3 fibroblasts deprived of growth factors and treated with IGF-1 (50 ng ml^−1^) and PI3K inhibitor (GDC-0941, 2 μM) with concurrent ^13^C_3_^15^N_1_-VB5 labelling (3 h). Cells were incubated with 1% serum but without growth factors (18 h) and pretreated with inhibitor (15 min) prior to treatments. Protein immunoblots probed with indicated antibodies (left); molecular weight markers in kD (right). Graphed metabolites measured by LC-MS/MS. Replicate samples (○); mean (bars); SEM (error bars); n = 3 biological replicates. *significant difference from treatment marked (†), one-way ANOVA with Tukey’s test, (p<alpha 0.05).

Next, we assessed whether processes other than increased de novo synthesis (for example, increased deacylation of acyl-CoAs, or decreased CoA usage or degradation^14^) contribute to PI3K-dependent accumulation of newly synthesized CoA. To test the effects of PI3K signalling on intracellular pools of CoA and acetyl-CoA disconnected from additional de novo synthesis, we pulse-labelled cells with ^13^C_3_^15^N_1_-VB5 and chased in medium lacking VB5, before stimulating with insulin. Prelabelled pools of CoA and acetyl-CoA did not expand in response to insulin, whereas PI3K signalling responded normally (Extended Data Fig. 3a). Moreover, CoA synthesized in a PI3K-dependent manner was used to form multiple short-chain acyl-CoAs (Fig. 1d; validation of LC–MS/MS methods is shown in Extended Data Fig. 3b). As an orthogonal approach for monitoring CoA synthesis, we pulse-labelled cells with radiolabelled ^14^C-VB5 and then chased in medium lacking VB5 to deplete free, unincorporated ^14^C-VB5. Stable radiolabel incorporation was insulin-responsive and PI3K-dependent, supporting our MS measurements (Fig. 1e and Extended Data Fig. 3c). Together, these data demonstrate that the PI3K-stimulated accumulation of newly synthesized CoA requires increased de novo synthesis and is not primarily due to effects on CoA deacylation, usage or degradation.

PI3K inhibition also decreased abundance of newly synthesized CoA and acyl-CoAs in SUM159, MDA-MB-468 and T47D human breast cancer cell lines grown with fetal bovine serum (FBS) and in mouse fibroblasts stimulated with IGF-1 (Extended Data Fig. 3d,e). Thus, PI3K regulates CoA synthesis in multiple mammalian cell types and species.

**Extended Data Fig. 4:**
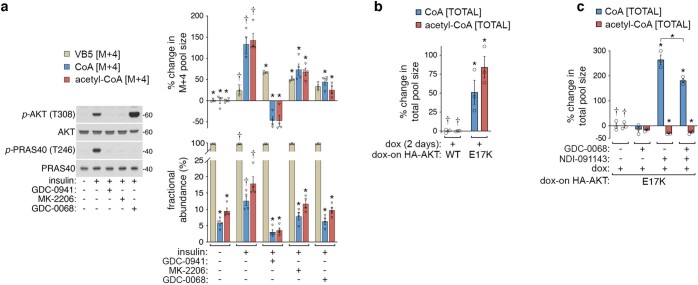
Data supporting Fig. 1. **a**, Insulin (100 nM), PI3K inhibitor (GDC-0941, 2 μΜ), and AKT inhibitor (MK-2206 or GDC-0068, 4 μΜ) treatments with ^13^C_3_^15^N_1_-VB5 labelling (3 h). Prior to treatments and labelling cells were serum/growth factor depleted (18 h) and pretreated with inhibitor (15 min). Protein immunoblots from one representative experiment probed with antibodies for total and phosphorylated (*p*) proteins (left); molecular weight markers in kD (right). Graphed metabolites measured by LC-MS/MS. Mean metabolite levels (bars) from four independent experiments (∇); SEM (error bars). Average for left-most treatment group set to 0% for each metabolite in % change graph. *significant difference from treatment marked (†), one-way ANOVA with Tukey’s test, (p<alpha 0.05). **b**, Total metabolite levels in MCF10A cells expressing doxycycline-inducible HA-tagged wild-type (WT) or constitutively active (E17K) AKT following doxycycline incubation (48 h) and serum/growth factor-deprivation (18 h). Replicate samples (○); mean (bars); SEM (error bars); n = 3 biological replicates. Average for left-most treatment group set to 0% for each metabolite. *significant difference from treatment marked (†), two-sided student’s t-test, (p<alpha 0.05). **c**, Total metabolite levels for experiment shown in main Fig. 1g. AKT inhibitor (GDC-0068, 2 μΜ) and ACLY inhibitor (NDI-091143, 15 μM) treatments with concurrent ^13^C_3_^15^N_1_-VB5 labelling (3 h) of MCF10A expressing doxycycline-inducible HA-tagged wild type (WT) or constitutively active (E17K) AKT. Cells were treated with doxycycline (200 ng ml^−1^; 48 h), serum/growth factor-deprived (18 h) and pre-treated (15 min) with inhibitor prior to labelling. Graphed metabolites measured by LC-MS/MS. Replicate samples (○); mean (bars); SEM (error bars); n = 3 biological replicates. Average for left-most treatment group set to 0% for each metabolite. *significant difference from treatment marked (†), one-way ANOVA with Tukey’s test, (p<alpha 0.05).

One of the primary effectors of PI3K in its regulation of metabolism is the protein kinase AKT^1,8^. Pharmacological inhibition of AKT significantly reduced insulin-stimulated CoA synthesis and increased VB5 accumulation, albeit to a lesser extent than PI3K inhibition, suggesting that regulation of CoA synthesis downstream of PI3K is multifaceted (Extended Data Fig. 4a). Under growth-factor-free conditions, cells inducibly expressing a cancer-derived constitutively active point mutant (E17K) of AKT exhibited increased AKT-dependent CoA synthesis relative to cells expressing wild-type (WT) AKT (Fig. 1f). This long-term, constitutive activation of AKT also increased the total pool sizes of CoA and acetyl-CoA (Extended Data Fig. 4b). Downstream of AKT, mechanistic target of rapamycin complex 1 (mTORC1) and its substrate ribosomal protein S6 kinase (S6K) are important metabolic regulators^1,15^. However, pharmacological inhibition of mTORC1 did not abolish AKT-stimulated CoA synthesis (Fig. 1f). These results indicate that AKT activation is sufficient to stimulate CoA synthesis and expand total CoA pools downstream of PI3K.

AKT phosphorylates and stimulates the activity of ATP citrate lyase (ACLY), the enzyme that produces acetyl-CoA from citrate and CoA^16,17^. To test whether this mechanism mediates the effects of AKT on CoA synthesis, AKT(E17K)-expressing cells were treated with a specific ACLY inhibitor^18^ before heavy VB5 labelling. ACLY inhibition reduced total acetyl-CoA pools as expected, but greatly increased both total and newly synthesized CoA pools without affecting AKT-dependent phosphorylation of ACLY (Fig. 1g and Extended Data Fig. 4c). This accumulation of free CoA is probably due to both decreased conversion to acetyl-CoA and decreased feedback inhibition of CoA synthesis by acetyl-CoA, which potently inhibits the pantothenate kinases^7,19^. Although total acetyl-CoA abundance decreased with ACLY inhibition, fractional labelling of acetyl-CoA increased, probably due to the activity of other acetyl-CoA-producing enzymes. Even with ACLY inhibition, CoA synthesis remained sensitive to AKT inhibition (Fig. 1g). These results demonstrate that AKT regulates CoA synthesis upstream of its established regulation of ACLY.

## PANK2 and PANK4 are AKT substrates

**Fig. 2:**
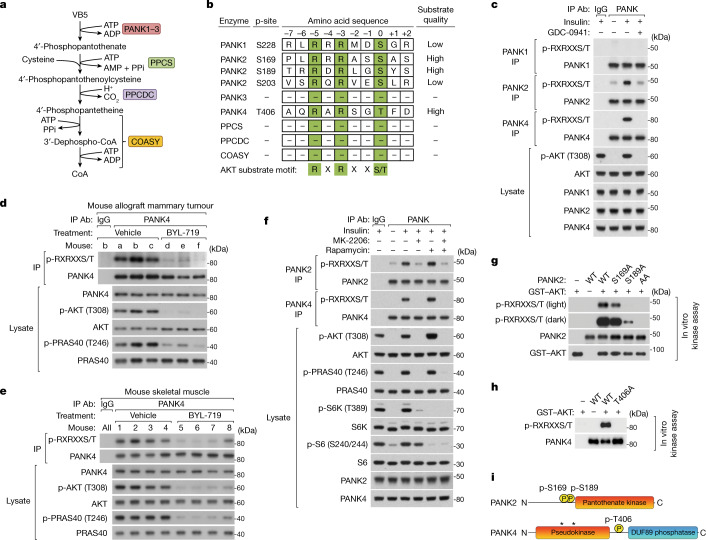
PANK2 and PANK4 are direct AKT substrates. **a**, CoA synthesis pathway enzymes. The full names and accession numbers are provided in Supplementary Table 1. **b**, Enzymes of the CoA synthesis pathway that are candidate AKT substrates. Amino acid residues that fall within low- to high-quality AKT substrate motifs according to the kinase–substrate prediction program Scansite, and that are reported to be phosphorylated in the phosphoproteomic database Phosphosite are listed. Numbering is based on the human sequence. **c**, Endogenous PANK1, PANK2 and PANK4 immunoprecipitation (IP) from MCF10A cells with insulin (100 nM) and PI3K inhibitor (GDC-0941, 2 μM) treatments (30 min), preceded by serum and growth factor deprivation (18 h) and inhibitor pretreatment (15 min). **d**, Endogenous PANK4 immunoprecipitation from orthotopic mammary allograft tumours in C57BL/6J treated with vehicle or PI3K inhibitor (BYL-719, 45 mg kg^−1^) daily for 10 days. **e**, Endogenous PANK4 immunoprecipitation from skeletal muscle (gastrocnemius) of C57BL/6J mice treated with PI3K inhibitor (BYL-719, 50 mg kg^−1^) for 1 h. **f**, Endogenous PANK2 and PANK4 immunoprecipitation from MCF10A cells treated with insulin (100 nM), AKT inhibitor (MK-2206, 2 μM), and mTORC1 inhibitor (rapamycin, 20 nM) with conditions otherwise as in **c**. **g**,**h**, In vitro AKT kinase assays. Untagged PANK2 (**g**) or PANK4 (**h**) (WT or alanine point mutants) were immunopurified from respective reconstituted knockout cells treated with PI3K and AKT inhibitors. PANK immunopurifications were incubated with purified GST–AKT (30 min). **i**, Diagram of the human PANK2 and PANK4 domains and AKT-targeted phosphorylation sites. The asterisks indicate the location of evolutionary mutations inactivating PANK4 kinase domain. For **c**–**h**, immunoblotting analysis probed for total and phosphorylated proteins including AKT phospho-substrate motifs; representative of two independent experiments. IgG, control IgG immunoprecipitation.

Next, we determined whether any enzymes of the CoA synthesis pathway^6^ (Fig. 2a) fit the criteria of an AKT substrate. Full AKT substrate consensus motifs (RXRXXS/T, in which R is arginine; X is any amino acid; and S/T is phosphorylated serine or threonine)^8,20^ were identified only on the pantothenate kinase family members PANK1, PANK2 and PANK4 (Fig. 2b). Of the four vertebrate PANK paralogues, PANK1 (α and β isoforms), PANK2 and PANK3 are catalytically active pantothenate kinases that produce 4′-phosphopantothenate in the first and rate-determining step of CoA synthesis^19,21–26^. In mammals, these PANKs localize to multiple compartments, including the cytosol (PANK1/3), nucleus (PANK1/2) and the intermembrane space of the mitochondria (PANK2)^6^. PANK2 has garnered the most attention as mutations in the *PANK2* gene cause a congenital disorder known as pantothenate-kinase-associated neurodegeneration (PKAN)^27^. The lesser-studied paralogue PANK4^26^, which is predicted to be cytosolic, is devoid of pantothenate kinase activity and is not known to regulate de novo CoA synthesis^9,28^.

**Extended Data Fig. 5:**
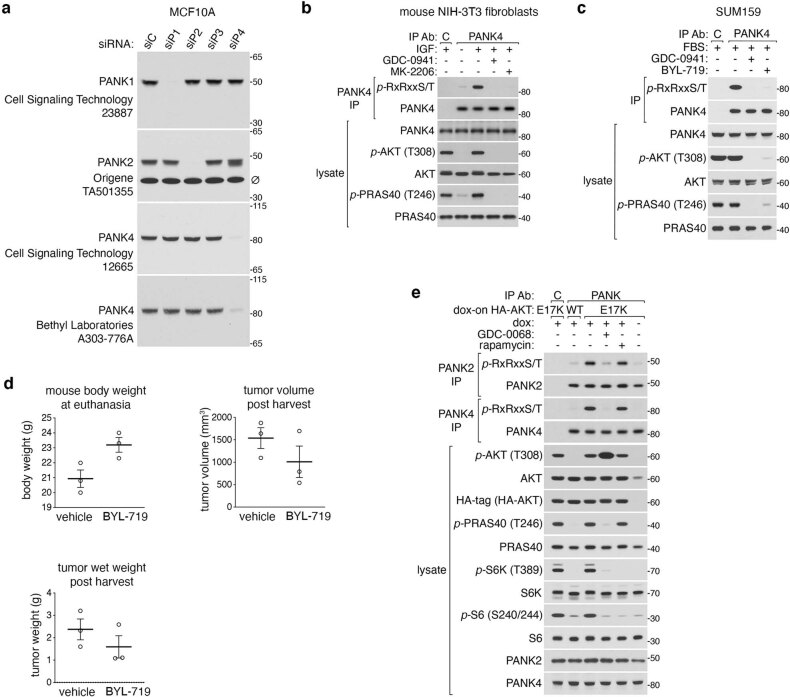
Data supporting Fig. 2. **a**, Validation of specificity for commercially available siRNAs and antibodies against endogenous PANK1/2/4. MCF10A cells were treated with a non-targeting control siRNA (siC) or siRNAs targeting individual PANK paralogs, PANK1–4 (siP1–4). Protein immunoblots of whole cell lysates were probed with the indicated PANK antibodies (manufacturer and catalogue numbers listed, left); molecular weight makers in kD (right); non-specific band (∅). Representative of two independent experiments. **b**, Immunopurifications (IPs) of endogenous PANK4 from growth factor-deprived NIH-3T3 mouse fibroblasts treated (30 min) with IGF-1 (50 ng ml^−1^), PI3K inhibitor (GDC-0941, 2 μM), and AKT inhibitor (MK-2206, 4 μM). Cells were incubated without growth factors but with 1% serum (18 h) and pretreated with inhibitors (15 min) prior to treatments. IPs and whole cell lysates were probed with a phospho-RXRXXS/T motif antibody and other indicated antibodies (left); molecular weight markers in kD (right). Representative of two independent experiments. **c**, IPs of endogenous PANK4 from SUM159 breast cancer cells grown in media with 10% fetal bovine serum (FBS) and treated (30 min) with two PI3K inhibitors (GDC-0941, 2 μM and BYL-719, 2 μM). IPs and whole cell lysates were probed with a phospho-RXRXXS/T motif antibody and other indicated antibodies (left); molecular weight markers in kD (right). Representative of two independent experiments. **d**, Body weight, tumour weight, and tumour volume corresponding to treatments of the mouse mammary allograft from main Fig. 2d. Replicate samples (○). Mean (horizontal line). SEM (error bars); n = 3 biological replicates. **e**, Endogenous PANK2 and PANK4 IPs from MCF10A cells expressing doxycycline-inducible HA-tagged wild-type (WT) or constitutively active (E17K) AKT, with AKT inhibitor (GDC-0068, 2 μM) or mTORC1 inhibitor (rapamycin, 20 nM) treatments (30 min) preceded by doxycycline incubation (48 h) and serum/growth factor-deprivation (18 h). IPs and whole cell lysates were probed with a phospho-RXRXXS/T motif antibody and other indicated antibodies (left); molecular weight markers in kD (right). Representative of two independent experiments.

Using an antibody that specifically recognizes phosphorylated AKT substrate motifs, we detected insulin-stimulated, PI3K-dependent phosphorylation of immunopurified endogenous PANK2 and PANK4, but not PANK1 (Fig. 2c; antibodies validated in Extended Data Fig. 5a). Available PANK4 antibodies yielded more robust immunopurifications than PANK2 antibodies, so we assessed PANK4 phosphorylation in additional settings. PI3K-dependent phosphorylation of PANK4 was also detected in mouse fibroblasts stimulated with IGF-1 and SUM159 cells grown with FBS (Extended Data Fig. 5b,c), mirroring effects on CoA synthesis in these cells (Extended Data Fig. 3d,e) and indicating conservation of this mechanism. Importantly, we detected PI3K-dependent phosphorylation of endogenous PANK4 in vivo. A ten-day PI3K inhibitor treatment of mice diminished PANK4 phosphorylation in *Pik3ca*^p.H1047R^-expressing mammary allograft tumours that exhibit PI3K-dependent growth^29^. A one-hour PI3K inhibitor treatment of non-tumour-bearing mice also decreased PANK4 phosphorylation in skeletal muscle, the tissue with the highest reported expression of PANK4^26^ (Fig. 2d,e and Extended Data Fig. 5d).

**Extended Data Fig. 6:**
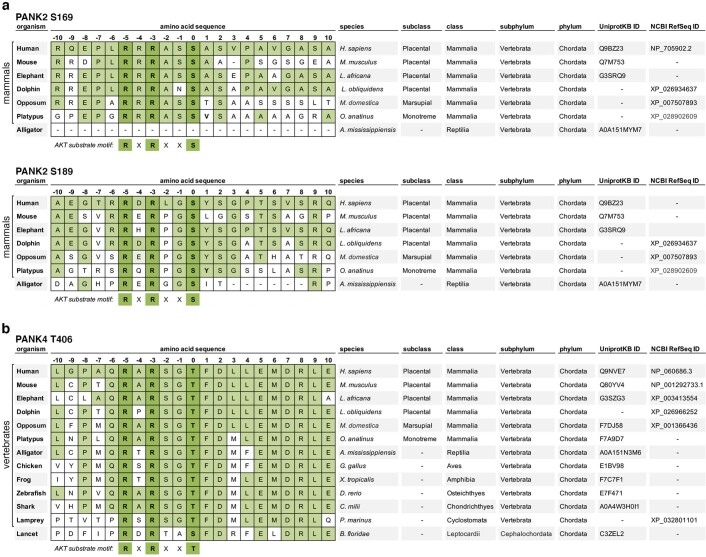
Data supporting Fig. 2. **a**, Protein sequence alignments of PANK2 orthologues from indicated species based on amino acids surrounding S169 and S189 of the human protein. **b**, Protein sequence alignments of PANK4 orthologues from indicated species based on amino acids surrounding T406 of the human protein.

PANK2 and PANK4 phosphorylation stimulated by insulin, IGF-1 or AKT(E17K) expression in the absence of growth factors was blocked after AKT inhibition but not after mTORC1 inhibition (Fig. 2f and Extended Data Fig. 5b,e). Purified AKT directly phosphorylated immunopurified wild-type PANK2 and PANK4 in vitro, and this phosphorylation was abrogated by mutating PANK2 Ser169 and Ser189 and PANK4 Thr406 to alanine (Fig. 2g–i). AKT substrate motifs on PANK2 are mostly conserved in mammals, whereas the motif on PANK4 is conserved across vertebrates (Extended Data Fig. 6a,b). Thus, in parallel with its effects on CoA synthesis, AKT phosphorylates PANK2 and PANK4 within well-conserved AKT substrate motifs.

## PANK4 suppresses CoA synthesis

**Fig. 3:**
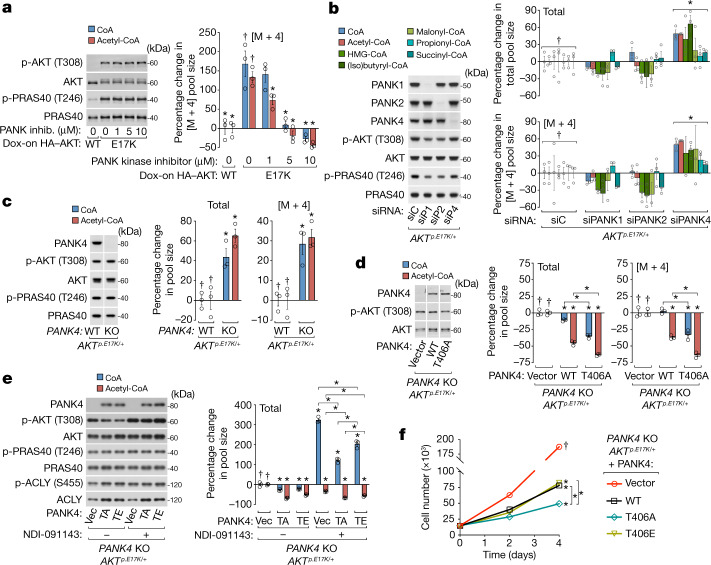
PANK4 suppresses CoA synthesis and phosphorylation of PANK4 Thr406 reduces this suppression. **a**, PANK kinase inhibitor (0–10 μM; inhib.) treatments with concurrent ^13^C_3_^15^N_1_-VB5 labelling (3 h) of MCF10A cells expressing doxycycline-inducible HA-tagged wild-type or constitutively active (E17K) AKT. Treatment and labelling were preceded by doxycycline incubation (48 h), serum and growth factor deprivation (18 h) and inhibitor pretreatment (15 min). **b**, Individual siRNA-mediated knockdowns of *PANK1*, *PANK2* and *PANK4* (siP1, siP2 and siP4, respectively) or non-targeting siRNA (siC) in MCF10A *AKT*^*p.E17K/+*^ cells with ^13^C_3_^15^N_1_-VB5 labelling (24 h) preceded by serum and growth factor deprivation (18 h). **c**, Wild-type *PANK4* and *PANK4*-KO *AKT*^*p.E17K/+*^ MCF10A cells with ^13^C_3_^15^N_1_-VB5 labelling (3 h) preceded by serum and growth factor deprivation (18 h). Divided blots are from same SDS–PAGE gel and image. **d**, *PANK4*-KO cells stably expressing vector (Vec) or untagged human PANK4 (WT or T406A) with conditions and labelling otherwise as described in **c**. Divided blots are from same SDS–PAGE gel and image. **e**, ACLY inhibitor (NDI-091143, 20 μM) treatment with concurrent ^13^C_3_^15^N_1_-VB5 labelling (3 h) of *PANK4*-KO cells stably expressing vector or untagged human PANK4 (T406A or T406E). Treatments and labelling were preceded by serum and growth factor deprivation (18 h) and inhibitor pretreatment (2 h). **f**, Two-dimensional (2D) proliferation of cell lines from **d** and **e** with growth factors. Statistical comparison was performed on day 4 data. For **a**–**e**, metabolites were measured using LC–MS/MS and normalized to protein; labelled metabolites (M + 4). For the graphs of percentage change, the mean value of the left-most treatment group was set to 0%. Immunoblotting analysis probed for total or phosphorylated proteins. For **a**–**f**, *n* = 3 biological replicates (circles). Data are mean ± s.e.m. Statistical analysis was performed using two-tailed Student’s *t*-tests (**c**), one-way ANOVA with Tukey test (**a** and **d**–**f**) and two-way ANOVA with Sidak test (**b**); asterisks (*) indicate significant differences compared with the treatment groups marked with daggers (†) or between treatments indicated with brackets (*P* < 0.05).

**Extended Data Fig. 7:**
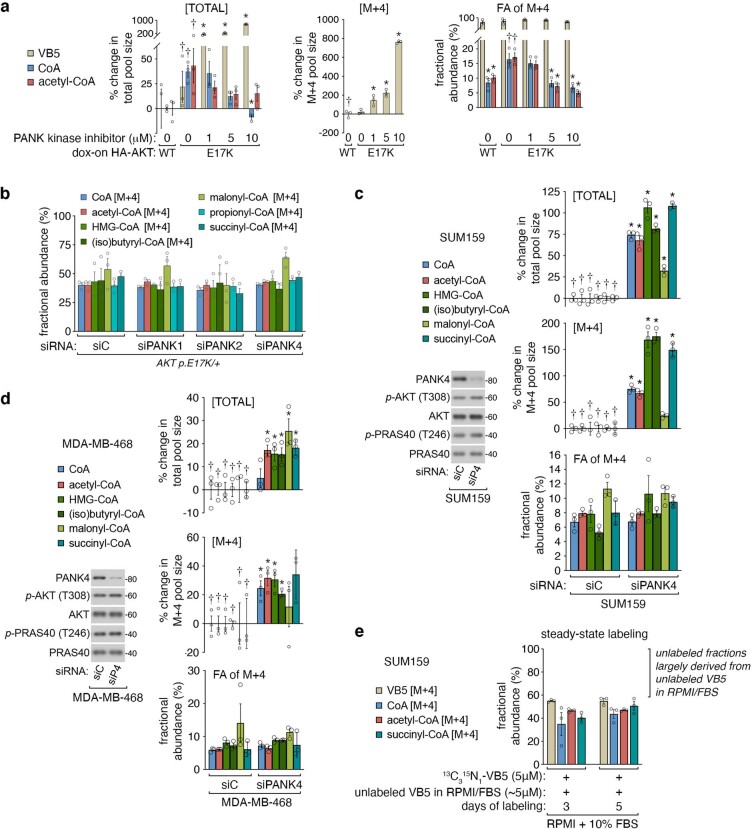
Data supporting Fig. 3. **a**, Total VB5, CoA, acetyl-CoA levels, and VB5 labelling data from main Fig. 3a. Graphed metabolites measured using LC-MS/MS. Replicate samples (○). Mean (bars). SEM (error bars); n = 3 biological replicates. *significant difference from treatment marked (†), one-way ANOVA with Tukey’s test, (p<alpha 0.05). **b**, Fractional abundance (M+4/total) of labelled CoA species in main Fig. 3b. Replicate samples (○). Mean (bars). SEM (error bars); n = 3 biological replicates. **c**, siRNA-mediated knockdown of PANK4 in SUM159 cells with ^13^C_3_^15^N_1_-VB5 labelling (3 hours) in the presence of 10% fetal bovine serum. Protein immunoblots probed with indicated antibodies (left); size markers in kD (right). Graphed metabolites measured by LC-MS/MS. Replicate samples (○). Mean (bars). SEM (error bars); n = 3 biological replicates. *significant difference from treatment marked (†), two-tailed student’s t-test, (p<alpha 0.05). **d**, siRNA-mediated knockdown of PANK4 in MDA-MB-468 cells with ^13^C_3_^15^N_1_-VB5 labelling (3 h). Cells were grown in media with dialysed fetal bovine serum (FBS). Protein immunoblots probed with indicated antibodies (left); size markers in kD (right). Graphed metabolites measured by LC-MS/MS. Replicate samples (○). Mean (bars). SEM (error bars); n = 3 biological replicates. *significant difference from treatment marked (†), two-tailed student’s t-test, (p<alpha 0.05). **e**, Steady state ^13^C_3_^15^N_1_-VB5 labelling (5 μM, 3–5 days) in SUM159 cells grown in RPMI and 10% FBS which contain ~5 μM unlabelled VB5. Graphed metabolites measured by LC-MS/MS and expressed as fractional abundance of labelled metabolites (M+4/total). Replicate samples (○). Mean (bars). SEM (error bars); n = 3 biological replicates.

**Extended Data Fig. 8:**
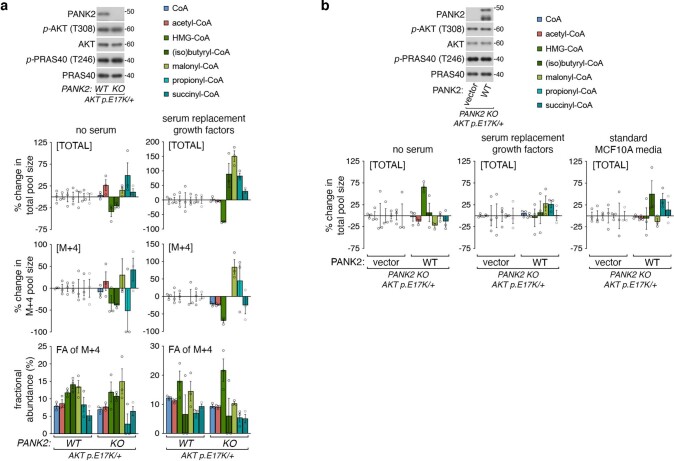
Data supporting Fig. 3. **a**, WT or PANK2 knockout *AKT p.E17K/+* MCF10A cells labelled with ^13^C_3_^15^N_1_-VB5 (3 h) in serum-free media with or without defined serum replacement and growth factors. Protein immunoblots probed with indicated antibodies (left); molecular weight markers in kD (right). Graphed metabolites measured using LC-MS/MS. Replicate samples (○). Mean (bars). SEM (error bars); n = 3 biological replicates. **b**, PANK2 knockout *AKT p.E17K/+* MCF10A cells stably expressing empty vector or WT PANK2 in serum-free media, media with defined serum replacement and growth factors, or standard MCF10A media. Protein immunoblots probed with indicated antibodies (left); molecular weight markers in kD (right). Graphed metabolites measured using LC-MS/MS. Replicate samples (○). Mean (bars). SEM (error bars); n = 3 biological replicates.

**Extended Data Fig. 9:**
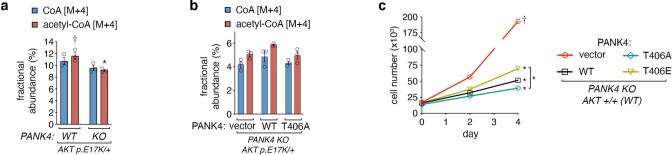
Data supporting Fig. 3. **a**, Fractional abundance (M+4/total) of labelled metabolites in main Fig. 3c. Replicate samples (○). Mean (bars). SEM (error bars); n = 3 biological replicates. *significant difference from treatment marked (†), two-tailed student’s t-test, (p<alpha 0.05). **b**, Fractional abundance (M+4/total) of labelled metabolites in main Fig. 3d. Replicate samples (○). Mean (bars). SEM (error bars); n = 3 biological replicates. *significant difference from treatment marked (†), two-tailed student’s t-test, (p<alpha 0.05). **c**, Two-dimensional proliferation assay with PANK4 KO *AKT+/+* MCF10A cells stably expressing empty vector or PANK4 (WT, T406A, or T406E) in serum-free media with serum replacement and growth factors over 4 days. Replicate samples (○). SEM (error bars); n = 3 biological replicates. *significant difference from treatment marked (†), one-way ANOVA with Tukey’s test, (p<alpha 0.05). Significance based on day 4 values. Representative of two independent experiments.

We next determined whether PANK2 and PANK4 mediate AKT-dependent regulation of CoA synthesis. First, using a specific inhibitor^30^, we established that blocking PANK kinase activity abolishes AKT-stimulated CoA synthesis. PANK kinase inhibition was also sufficient to cause a substantial accumulation of VB5 (Fig. 3a and Extended Data Fig. 7a). We next used short interfering RNA (siRNA)-mediated knockdowns (validated in Extended Data Fig. 5a) to individually deplete PANK1, PANK2 and PANK4 in *AKT*^*p.E17K/+*^ knock-in MCF10A cells. PANK1 or PANK2 depletion trended towards reducing CoA synthesis, but PANK4 depletion unexpectedly increased CoA synthesis (Fig. 3b and Extended Data Fig. 7b). Similar results were obtained in SUM159 and MDA-MB-468 cells cultured in FBS (Extended Data Fig. 7c,d). Steady-state labelling of SUM159 cells indicated that, even in the presence of FBS, VB5-initiated de novo synthesis was the major source of CoA (Extended Data Fig. 7e). To study these effects further, we generated *AKT*^*p.E17K/+*^ MCF10A cells with CRISPR-mediated knockouts (KOs) of *PANK2* and *PANK4*. In *PANK2-*KO cells and derivative lines stably reconstituted with PANK2, we were unable to detect consistent differences in CoA synthesis and abundance under various conditions, precluding further study of PANK2 in this context (Extended Data Fig. 8a,b). These results are probably due to compensation by PANK1/3, and are consistent with previous studies that similarly did not detect changes in CoA abundance in human *PANK2*-knockdown cells^31^ or adult *PANK2-*KO mouse tissues^32^. However, as with our *PANK4*-knockdown cells, *PANK4*-KO cells exhibited increased CoA synthesis (Fig. 3c and Extended Data Fig. 9a). Given that the cellular function of PANK4 is poorly understood^9^ and the AKT substrate motif on PANK4 is well conserved (Extended Data Fig. 6b), we focused on defining the regulation and function of PANK4 in CoA synthesis.

We stably reconstituted *PANK4-*KO cells with wild-type PANK4, phosphorylation-deficient PANK4 (T406A) or phosphomimetic PANK4 (T406E). CoA synthesis was reduced after re-expression of wild-type PANK4 and further reduced after re-expression of PANK4(T406A), indicating that this unphosphorylated mutant has an enhanced ability to suppress CoA synthesis (Fig. 3d and Extended Data Fig. 9b). To dissociate PANK4-mediated regulation of CoA synthesis from downstream CoA usage by ACLY and feedback inhibition of PANK1–3 by acetyl-CoA^7,19^, we inhibited ACLY in *PANK4*-KO cells expressing PANK4(T406A) or PANK4(T406E). The accumulation of total CoA observed with ACLY inhibition was reduced by PANK4(T406A), while the suppressive effect of PANK4(T406E) was significantly impaired (Fig. 3e). Consistent with these effects on CoA synthesis, proliferation was reduced in both *AKT*^*+/+*^ and *AKT*^*p.E17K/+*^ cells expressing PANK4, and PANK4(T406A) further reduced proliferation relative to PANK4(T406E). The proliferation rate of cells expressing wild-type PANK4 varied relative to the Thr406 mutants (Fig. 3f and Extended Data Fig. 9c). Together, these data demonstrate that PANK4 suppresses CoA synthesis and AKT-mediated phosphorylation of Thr406 attenuates this function of PANK4 with the net effect of promoting CoA synthesis and cell proliferation.

## PANK4 functions as a phosphatase

**Fig. 4:**
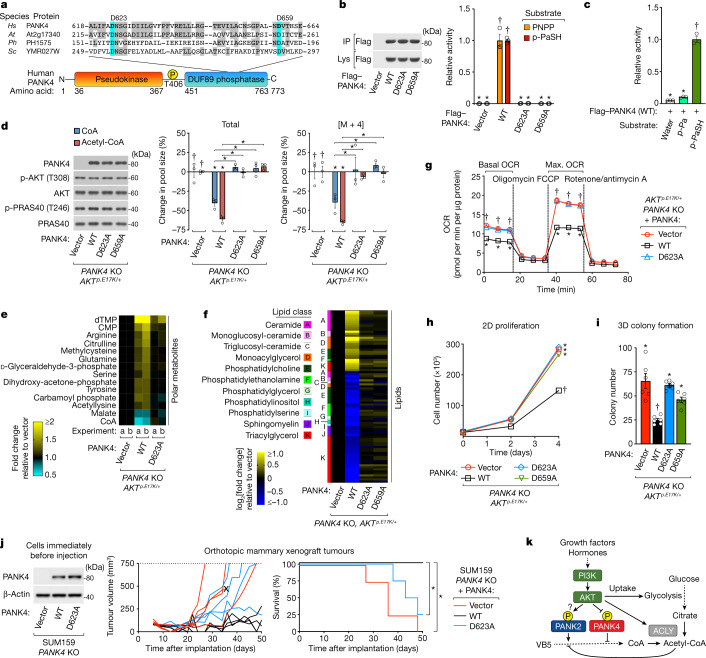
PANK4 functions as a metabolite phosphatase. **a**, Amino acid alignment of PANK4 with conserved catalytic aspartates (blue, bold) and other conserved residues (grey) of previously characterized DUF89 domains (non-PANK4 orthologues). *At*, *Arabidopsis thaliana*; *Hs*, *Homo sapiens*; *Ph*, *Pyrococcus horikoshii*; *Sc*, *Saccharomyces cerevisiae*. **b**, PANK4 phosphatase assay. Flag-tag immunopurifications from cells expressing vector or Flag–PANK4 (WT, D623A or D659A). Substrates: para-nitrophenyl phosphate (PNPP) and 4′-phosphopantetheine (*p*-PaSH). The wild-type mean was set to 1. **c**, PANK4 phosphatase assay. Flag–PANK4 as in **b**. Substrates: 4′-phosphopantothenate (*p*-Pa) and 4′-phosphopantetheine. The 4′-phosphopantetheine mean was set to 1. **d**, *PANK4*-KO *AKT*^*p.E17K/*+^ MCF10A cells stably expressing vector or untagged PANK4 (WT, D623A or D659A). ^13^C_3_^15^N_1_-VB5 labelling (3 h) was performed with serum replacement and growth factors. Metabolites were measured using LC–MS/MS and normalized to protein. Labelled metabolites (mass + 4). Vector mean set to 0%. **e**, Unlabelled polar metabolomics using cells and conditions in **d**.Two independent experiments, ‘a’ and ‘b, were analysed (Methods). **f**, Unlabelled lipidomics using the cells and conditions as described in **d**. The analysis incorporates three independent experiments (Methods). **g**, Seahorse oxygen-consumption assay using the cells in **d** without serum or growth factors. OCR, oxygen consumption rate. **h**, 2D proliferation with the cells and conditions as described in **d**. Representative of three independent experiments. **i**, Three-dimensional (3D) soft agar colony formation using the cells in **d** with serum and growth factors. **j**, Orthotopic mammary xenograft tumours using SUM159 *PANK4-*KO cells with stable expression of vector or untagged PANK4 (WT or D623A) in nude mice. Individual tumour growth curves (left). Kaplan–Meier survival curves using tumour volume (750 mm^3^) or ulceration (X) end points (right). **k**, Model of PI3K-dependent CoA synthesis regulation. For **b**, **d** and **j**, immunoblotting analysis probed for total and phosphorylated proteins. For **b**–**d** and **g**–**i**, *n* = 3 (**b**–**d**, **g** and **h**), *n* = 4 (**j**) or *n* = 6 (**i**) biological replicates (circles). Data are mean ± s.e.m. For **b**–**d** and **g**–**j**, statistical analysis was performed using one-way ANOVA with Tukey test; asterisks (*) indicate significant differences compared with the treatment groups marked with daggers (†) or between treatments indicated with brackets (*P* < 0.05). Source data

**Extended Data Fig. 10:**
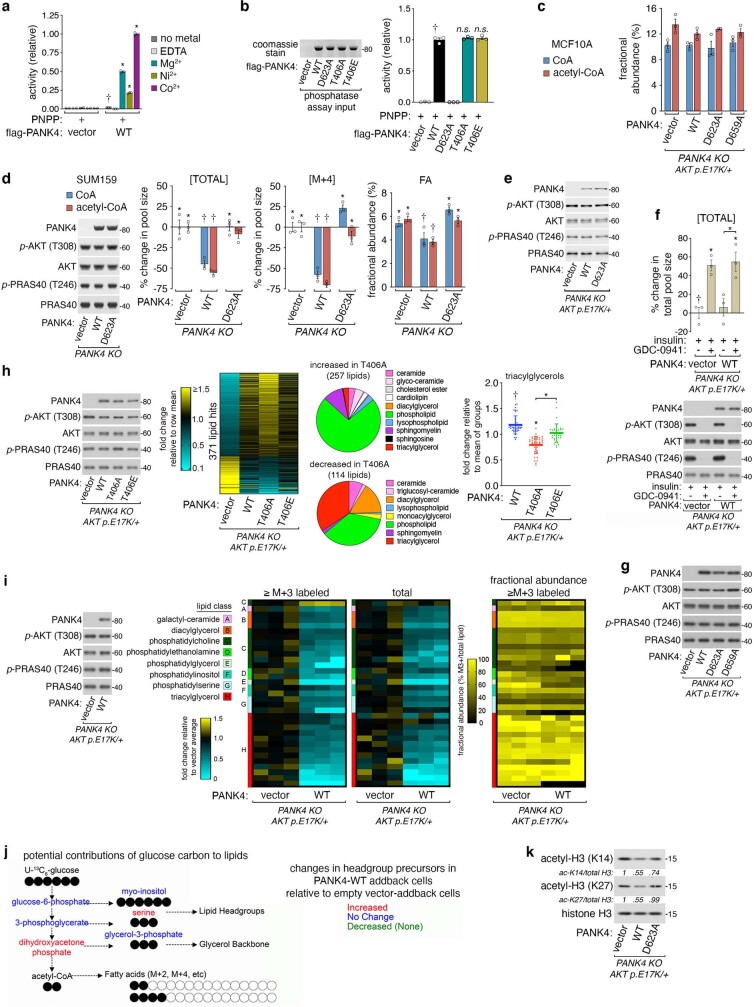
Data supporting Fig. 4. **a**, Divalent metal cation dependence of *in vitro* PANK4 phosphatase activity. Flag-tag immunopurifications from HEK-293T cells expressing empty vector or full-length wild-type flag-PANK4 were incubated (30 min, 30 °C) with no metal, the chelator EDTA, or indicated metals and the substrate para-nitrophenyl phosphate (PNPP). Chromogenic reaction products measured by spectrophotometry. Relative activity with Co^2+^ reaction set to 1. Replicate samples (○). Mean (bars). SEM (error bars); n = 3 biological replicates. *significant difference from treatment marked (†), one-way ANOVA with Tukey’s test, (p<alpha 0.05). **b**, *In vitro* PANK4 phosphatase assay with PANK4 phospho-mutants. Flag-tag immunopurifications from HEK-293T cells expressing empty vector or full-length flag-PANK4 (WT, T406A, or T406E) were incubated (30 min, 30 °C) with Co^2+^ and the substrate PNPP. Chromogenic reaction products measured by spectrophotometry. Relative activity with WT reaction set to 1. Replicate samples (○). Mean (bars). SEM (error bars); n = 3 biological replicates. *significant difference from treatment marked (†), one-way ANOVA with Tukey’s test, (p<alpha 0.05). **c**, Fractional abundance (M+4/total) of labelled metabolites in main Fig. 4d. Replicate samples (○). Mean (bars). SEM (error bars); n = 3 biological replicates. **d**, SUM159 PANK4 KO cells stably expressing empty vector, PANK4-WT, or PANK4-D623A with ^13^C_3_^15^N_1_-VB5 labelling (3 h), in the presence of 10% fetal bovine serum. Protein immunoblots probed with indicated antibodies (left); molecular weight markers in kD (right). Graphed metabolites measured by LC-MS/MS. Replicate samples (○). Mean (bars). SEM (error bars); n = 3 biological replicates. *significant difference from treatment marked (†), one-way ANOVA with Tukey’s test, (p<alpha 0.05). **e**, Protein immunoblot associated with main Fig. 4e. Probed with indicated antibodies (left); molecular weight markers in kD (right). **f**, Insulin (100 nM) and PI3K inhibitor (GDC-0941, 2 µM) treatment (3 h) in PANK4 KO *AKT p.E17K/+* MCF10A cells stably expressing empty vector or PANK4-WT. Cells were pretreated with inhibitor (15 min) prior to insulin stimulation. Total VB5 levels measured by LC-MS/MS. Replicate samples (○). Mean (bars). SEM (error bars); n = 3 biological replicates. *significant difference from treatment marked (†), one-way ANOVA with Tukey’s test, (p<alpha 0.05). **g**, Protein immunoblot associated with main Fig. 4f. Probed with indicated antibodies (left); molecular weight markers in kD (right). **h**, Mass spectrometry-based lipidomics analysis of PANK4 KO *AKT p.E17K/+* MCF10A cells stably expressing empty vector or PANK4 (WT, T406A, or T406E) grown in serum-free media with serum replacement and growth factors. Protein immunoblots probed with indicated antibodies (left); molecular weight markers in kD (right). Significantly altered lipids (n = 3 biological replicates, one-way ANOVA, false discovery rate < 0.1, fold change > 25%) that correlated with CoA levels between cell lines (Patternhunter, Metaboanalyst 4.0, false discovery rate < 0.1) are represented by heatmap as fold change relative to the row mean for each respective lipid species. Lipid classes altered by PANK4 T406 phosphorylation status are represented in pie charts. Relative levels of triacylglycerols in PANK4-expressing cell lines are graphed separately as fold change relative to the mean of the three PANK4-expressing samples for each given triacylglycerol. Replicate samples (○). Mean (bars). SD (error bars); n = 39 triacylglycerol species. *significant difference from treatment marked (†), one-way ANOVA with Tukey’s test, (p<alpha 0.05). **i**, ^13^C_6_-glucose labelling (16 h) of PANK4 KO *AKT p.E17K/+* MCF10A cells stably expressing empty vector or PANK4-WT, grown in serum-free media with serum replacement and growth factors, followed by mass spectrometry-based lipidomics analysis. Protein immunoblots probed with indicated antibodies (left); molecular weight markers in kD (right). Since M+3 and above lipid isotopologues were enriched in labelled over unlabelled samples, the lipids for which the total signal for M+3 and above isotopologues was significantly different between vector and PANK4-WT cell lines (n = 3 biological replicates, two-sided student’s t-test, false discovery rate < 0.1, fold change > 20%) were represented by heatmap as fold change relative to the average of vector control samples. Total levels and fractional abundance ((sum of ≥M+3)/total) of significantly altered labelled lipid species are also shown by heatmap. Lipid classes colour- and letter-coded (A-H). **j**, Diagram of labelled ^13^C_6_-glucose tracing into metabolites that generate fatty acids, glycerol backbones, and phospholipid headgroups. Polar metabolites were measured by mass spectrometry from PANK4 KO *AKT p.E17K/+* MCF10A cells expressing empty vector, PANK4-WT or PANK4-D623A, and grown in serum-free media with serum replacement and growth factors. Metabolites are colour-coded by significant increase (red), no change (blue), and decrease (green) in PANK4-WT-expressing cells relative to empty-vector-expressing cells. **k**, Immunoblots of acid-extracted histones from PANK4 KO *AKT p.E17K/+* MCF10A cells stably expressing empty vector, PANK4-WT, or PANK4-D623A, and grown in serum-free media with serum replacement and growth factors. Probed with indicated antibodies (left); molecular weight markers (right). Band intensities were quantified using ImageJ and acetyl-histone abundance was normalized to total histone abundance. Relative normalized acetyl-histone quantities are shown below each acetyl-histone band with the vector group set to 1. Representative of two independent experiments. Source data

Finally, we sought to determine the molecular mechanism through which PANK4 suppresses CoA synthesis. Evolutionary mutations have rendered the PANK4 kinase domain inactive in amniotes^9,28^. However, PANK4 possesses a second, minimally characterized domain belonging to the DUF89 family of metal-dependent metabolite phosphatases^33^ (Fig. 4a). In vitro, the isolated DUF89 domain of human PANK4 can dephosphorylate the third CoA synthesis intermediate 4′-phosphopantetheine and its oxidized derivatives^33^. However, in the few studies that have reported phenotypes associated with PANK4, an enzymatic activity has not been implicated^34,35^, and the specific cellular function of PANK4 remains to be established^9^. Nonetheless, PANK4 is highly conserved with orthologues containing both a predicted pseudokinase and phosphatase domain existing in animals, plants and fungi. Using immunopurified full-length human PANK4 and a standard small-molecule substrate, we confirmed that PANK4 exhibits divalent metal cation-dependent phosphatase activity in vitro^33^ (Extended Data Fig. 10a). Two conserved aspartate residues are essential for the phosphatase activity of previously characterized DUF89 domains^33^. Amino acid alignments predicted that Asp623 and Asp659 are essential for PANK4 phosphatase activity (Fig. 4a) and, indeed, phosphatase activity was abolished after mutation of either of these residues (Fig. 4b). In the presence of excess substrate, the activity of PANK4 was considerably higher towards 4′-phosphopantetheine (the third CoA synthesis intermediate) than towards 4′-phosphopantothenate (the first CoA synthesis intermediate) (Fig. 4c). We were unable to detect differences in the phosphatase activity of PANK4(T406) mutants in the presence of excess substrate (Extended Data Fig. 10b).

Stable re-expression of wild-type and phosphatase-dead mutants (D623A and D659A) of PANK4 in both MCF10A and SUM159 *PANK4*-KO cell lines revealed that the ability of PANK4 to inhibit CoA synthesis is completely dependent on its phosphatase activity (Fig. 4d and Extended Data Fig. 10c,d). Out of 226 polar metabolites measured, CoA exhibited the greatest consistent PANK4 phosphatase-dependent decrease in abundance (Fig 4e and Extended Data Fig. 10e). Accumulation of VB5 in response to PI3K inhibition did not require PANK4, consistent with inhibition of PANK kinase activity being sufficient for this effect (Extended Data Figs. 7a and 10f). To assess the extended metabolic consequences of the PANK4-dependent reduction in CoA abundance, we measured the effects on several CoA-dependent cellular processes. MS-based lipidomics analyses showed that PANK4 significantly alters the cellular lipid profile in a manner that is dependent on its phosphatase activity and phosphorylation state (Fig. 4f and Extended Data Fig. 10g,h). PANK4 reduced ^13^C_6_-glucose labelling of a subset of lipids without reducing glycerol backbone and headgroup precursor abundance (Extended Data Fig. 10i,j), suggesting that CoA-dependent fatty acid synthesis and/or lipid assembly was impaired. Furthermore, PANK4-expressing cells exhibited a phosphatase-activity-dependent decrease in oxygen consumption, indicating impaired mitochondrial respiration (Fig. 4g), and a reduction in histone acetylation, which is dependent on nuclear acetyl-CoA^5^ (Extended Data Fig. 10k). Finally, we found that the ability of PANK4 to suppress cell proliferation in a two-dimensional assay, colony formation in a three-dimensional soft agar assay and tumorigenesis in an orthotopic mammary xenograft mouse model (Fig. 4h–j) was completely dependent on its phosphatase activity.

## Discussion

Our study demonstrates that, in conjunction with metabolite-mediated feedback, PI3K–AKT signalling regulates flux through the de novo CoA synthesis pathway (Fig. 4k). Our results also reveal a specific cellular function for the highly conserved PANK4, which we propose directly limits the rate of CoA synthesis through its metabolite phosphatase activity against 4′-phosphopantetheine and possibly other related substrates. PANK4 is uniquely positioned to regulate CoA synthesis initiated not only from VB5 but also 4′-phosphopantetheine, which can be imported from extracellular sources^36^. PANK4 may also process damaged 4′-phosphopantetheine derivatives as previously proposed^33^. Beyond regulating the rate of CoA synthesis, PANK4 may serve to shunt its product into different compartments or uses that are not yet well understood. The regulatory mechanisms described here grant hormone and growth factor signalling fundamental control over CoA and acetyl-CoA-dependent metabolism and proliferation. Future studies could assess the therapeutic potential of inhibiting PANK4 phosphatase activity to increase CoA synthesis in patients with pantothenate-kinase-associated neurodegeneration or selectively inhibiting CoA synthesis in PI3K-dependent cancers.

## Methods

### Cell lines and culture conditions

#### Cell lines

The following commercially available cell lines were used: MCF10A (ATCC, CRL-10317); MCF10A with *PIK3CA*^*p.H1047R/+*^ knockin and matched *PIK3CA*^+/+^ control (PerkinElmer/Horizon Discovery, HD 101-011); MCF10A with *AKT1*^*E17K/+*^ (Perkin Elmer/Horizon Discovery, HD 101-007); SUM159 (Asterand Bioscience/BioIVT, SUM159PT); MDA-MB-468 (ATCC, HTB-132); T47D (ATCC, HTB-133); NIH-3T3 mouse embryonic fibroblasts (ATCC, CRL-1658); HEK293T (ATCC, CRL-11268). The MCF10A cell lines stably expressing doxycycline-inducible AKT2-E17K from the pTRIPZ vector were previously described^37^.

#### Maintenance culture conditions

All MCF10A-derived cell lines were maintained in standard MCF10A growth medium without antibiotics (DMEM/F12 medium (Wisent Bioproducts, 319-075-CL), 5% horse serum (Gemini Bio, 100508), 10 µg ml^−1^ insulin (Thermo Fisher Scientific/Gibco, A11382II), 0.5 mg ml^−1^ hydrocortisone (Sigma-Aldrich, H4001), 20 ng ml^−1^ EGF (R&D Systems, 236-EG-01M) and 100 ng ml^−1^ cholera toxin (List Biological Laboratories, 100B)). MCF10A stably expressing doxycycline-inducible HA-AKT2-WT and HA-AKT2-E17K (pTRIPZ constructs) were maintained in 0.5 μg ml^−1^ puromycin. NIH-3T3 mouse fibroblasts were grown in DMEM (Wisent Bioproducts, 319-005-CL) with 10% heat-inactivated FBS (Thermo Fisher Scientific, 10438026). SUM159, MDA-MB-468 and T47D cell lines were maintained in RPMI 1640 (Wisent Bioproducts, 350-000-CL) with 10% heat-inactivated FBS. Cell lines stably expressing PANK2 or PANK4 variant constructs in pLenti-Ubc-IRES-Neo (pLUIN) were maintained in 250 µg ml^−1^ G418 (Geneticin; neomycin analogue) (Invivogen, ant-gn-1). All of the cell lines were grown at 37 °C under 5% CO_2_ and high humidity, and were typically trypsinized and split every 2 days, keeping cell densities subconfluent. Cells were confirmed to be negative for mycoplasma contamination using the MycoAlert Detection Kit (Lonza, LT07-218).

#### Induction of HA–AKT2 with doxycycline

For experiments involving MCF10A stably expressing doxycycline-inducible HA–AKT2(WT) or HA–AKT2(E17K), cells were plated in standard MCF10A growth medium containing 200 ng ml^−1^ doxycycline (Sigma-Aldrich, D3447) for 30 h and then serum/growth factor deprived in medium containing 200 ng ml^−1^ doxycycline for 18 h for a total of 48 h. Doxycycline was not included in the medium on the day that cells were treated and collected.

### RNA interference using siRNAs

#### MCF10A siRNA knockdowns

Stocks of siRNA were prepared by resuspending siRNAs in 1× siRNA buffer (5× siRNA buffer, (PerkinElmer/Horizon Discovery, B-002000-UB-100), diluted to 1× in sterile molecular-grade RNase-free water (PerkinElmer/Horizon Discovery, B-003000-WB-100)) for a stock concentration of 20 μM. After addition of buffer, stocks were vortexed for 30 min at room temperature, aliquoted into screwcap vials with gaskets, snap-frozen in liquid nitrogen and stored at −80 °C. Each aliquot was freeze–thawed no more than five times. For each target, three different validated siRNAs were pooled at equal concentrations such that the final total concentration in culture medium was 20 nM. For siRNA cell culture treatments in 6 cm dishes, siRNAs were diluted in 250 μl of prewarmed OptiMEM (Thermo Fisher Scientific/Gibco, 31985062) in tube A so that the final concentration on cells would be 20 nM total. At the same time, Lipofectamine RNAiMAX transfection reagent (Thermo Fisher Scientific/Invitrogen, 13778075) was diluted in 250 μl prewarmed OptiMEM in tube B so that the final concentration on cells would be 2.5 μl lipofectamine per ml medium in plate. These tubes were mixed by flicking (vortexing was avoided). Tube A was thoroughly mixed with tube B, incubated at room temperature for 15 min and added dropwise to cells. Cells were incubated with the siRNA/transfection reagent overnight and the medium was completely replaced with fresh growth medium the next day. About 30 h after transfection, the cells were washed with serum/growth-factor-free medium and deprived of serum/growth factors for 18 h (in custom DMEM as described above). These cells were then used in metabolite profiling experiments as described below. See Supplementary Table 1 (siRNA) for the siRNA sequences.

#### Breast cancer cell line siRNA knockdowns

Cells were seeded in 15 cm plates to reach 70% confluency the next day. Cells were transfected with siRNA as they were seeded, with 20 nM final siRNA mixture and 2.5 μl Lipofectamine per ml final volume in the 15 cm plate. After 24 h, cells were again trypsinized and seeded in 6 cm plates to reach 70% confluency the next day. Cells were seeded in RPMI 1640 medium supplemented with either 5% FBS or 5% dialysed FBS (Life Technologies, 26400-044). Then, 16–20 h later, 3 µM ^13^C_3_^15^N_1_-VB5 was spiked into the medium and tracing experiments were performed as described below.

### CRISPR–Cas9 knockouts

Snapgene v.5.1 was used for all cloning methodology and processing sequencing data.

#### *PANK2* knockout

A single guide RNA (sgRNA) targeting exon 2 of human *PANK2* (Supplementary Table 1 (sdRNA)) was inserted into TLCV2, a pLentiCRISPRv2-based vector modified for doxycycline-inducible expression of Cas9-2A–eGFP, and constitutive expression of the sgRNA and the puromycin resistance marker (Addgene plasmid, 87360, created by the laboratory of A. Karpf^38^). The guide targeting sequence was identified using E-CRISP^39^ (http://www.e-crisp.org/). Guide oligos were synthesized by Integrated DNA Technologies and inserted into TLCV2 as previously described for the pLentiCRISPRv2 vector^40,41^. Plasmids were transformed into NEB Stable competent high-efficiency *Escherichia coli* (New England Biolabs, C3040H). Clones were sequenced to confirm the insertion. *AKT1*^*E17K/+*^ MCF10A cells were infected with lentivirus containing *PANK2* sgRNA-TLCV2 constructs and selected with 1 µg ml^−1^ puromycin until control cells infected with lentivirus lacking TLCV2 were completely dead. To induce Cas9 expression, cells were maintained in doxycycline for 5 days at 250 ng ml^−1^, followed by 12 days at 1 µg ml^−1^. To prepare live cells for GFP-based fluorescence-activated cell sorting (FACS), cells were trypsinized, resuspended in FACS medium (DMEM without phenol red, glucose, glutamine or pyruvate (Thermo Fisher Scientific/Gibco, A1443001), supplemented with 1× penicillin–streptomycin (Thermo Fisher Scientific, 15140163), 5 µg ml^−1^ plasmocin (InvivoGen, ant-mpt-1), 10 mM HEPES (Thermo Fisher Scientific/Gibco, 15630080), 2% horse serum (Gemini Bio, 100508), 20 mM glucose (Thermo Fisher Scientific, 15023021), 1 mM pyruvate (Sigma-Aldrich, P5280)) and passed through a 40 μm strainer (Thermo Fisher Scientific, 22–363–547) to remove cell clumps. GFP^+^ cells with the top 25% highest fluorescence intensity (but excluding cells with the highest 5% intensity) were sorted. Cells grown without doxycycline (GFP negative) were used as a negative control for gating. Cells were collected in catch medium (DMEM/F12, 1× penicillin–streptomycin, 5 µg ml^−1^ plasmocin, 10 mM HEPES, 10% horse serum, 1 mM glutamine and 1 mM pyruvate). Cells were subsequently plated in standard MCF10A growth medium but with 10% horse serum, 10 mM HEPES, 1× penicillin–streptomycin and 5 µg ml^−1^ plasmocin at limiting dilution (0.5 cells per 100 µl) in 100 µl into 96-well plates with the goal of plating a single cell per well. Single-cell clones were grown into clonal populations and screened for PANK2 expression using western blotting. See Supplementary Table 1 (sgRNA) for the sgRNA sequences.

#### *PANK4* knockout

CRISPR–Cas9-mediated knockouts of *PANK4* were created using paired sgRNAs in conjunction with a Cas9 D10A nickase mutant that reduces the probability of off-target double-stranded DNA breaks. Transient transfections of cells were used to introduce the vectors expressing sgRNAs and Cas9 to ensure that the final clones did not express these and could be used to eventually create knockout cell lines stably re-expressing PANK4 with unaltered expression constructs. Paired sgRNAs targeting exon 2 of human *PANK4* (Supplementary Table 1 (sgRNA)) were separately inserted into pSpCas9n(BB)-2A-GFP (PX461) (Addgene plasmid, 48140, created by the laboratory of F. Zhang^41^). The two paired guide sequences were identified using E-CRISP^39^. Guide oligos were synthesized by Integrated DNA Technologies and inserted into pSpCas9n(BB)-2A-GFP as previously described^41^. The two vectors (containing one gRNA sequence each) were transiently co-transfected into *AKT1*^*E17K/+*^ MCF10A, *AKT1*^*+/+*^ MCF10A or SUM159 cells using Lipofectamine 2000 (Thermo Fisher Scientific/Invitrogen, 11668027). Then, 2 days after transfection, GFP^+^ cells were sorted, plated, grown into single-cell clonal populations and screened for PANK4 protein expression as described above for *PANK2*-KO cells. See Supplementary Table 1 (sgRNA) for the sgRNA sequences.

### Site-directed mutagenesis of phosphorylation sites

Site-directed mutagenesis of phosphorylated residues on PANK2 and PANK4 was performed using primers designed with NEBasechanger (New England Bioloabs), the Q5 Site-Directed Mutagenesis Kit (New England Biolabs, E0552S) and NEB Stable competent high-efficiency *E. coli* (New England Biolabs, C3040H).

### Flag-tagged PANK4 expression constructs (pcDNA3.1)

N-terminally Flag-tagged PANK4 (Flag-PANK4) constructs in pcDNA3.1 consisted of the flag-tag sequence (DYKDDDDK), followed by a glycine-serine linker (GS), followed by the sequence of full-length human PANK4 lacking its N-terminal methionine. PANK4 variants included the mutants D623A, D659A, T406A and T406E. Empty pcDNA3.1 was used as a control for transfections. These constructs were transiently transfected into HEK293T cells to produce Flag–PANK4 for in vitro phosphatase assays (see below).

### Construction of lentiviral protein expression constructs (pLUIN)

#### pLUIN cloning

Untagged human *PANK2* and *PANK4* sequences (accession numbers listed below) were inserted into a pLenti-Ubc-IRES-Neo (pLUIN) lentiviral vector (a gift from S. McBrayer and W. Kaelin^42^) using the In-Fusion HD Cloning System (Takara/Clontech, 639645). pLUIN consists of a ubiquitin C (*Ubc*) promotor driving expression of the protein of interest and an internal ribosome entry site (IRES) sequence driving expression of a G418/neomycin-resistance cassette. Except for the neomycin-resistance cassette, pLUIN is the same as the pLenti-Ubc-IRES-hygro construct described previously^42^. See Supplementary Table 1 (InFusion primers and Sequencing primers) for the sequences.

### Lentivirus production

Lentivirus was generated by transient transfection of the plasmid of interest into HEK293T cells. Cells were plated at 9 × 10^6^ cells per 10 cm plate and transfected with 6.3 μg of the protein-expression plasmid of interest, 11.1 µg psPAX2 packaging plasmid (Addgene plasmid, 12260, created by the laboratory of D. Trono), 0.6 μg pCMV-VSV-G envelope protein plasmid (Addgene plasmid 8454, created by the laboratory of B. Weinberg^43^) and 3 μl of polyethylenamine (Millipore-Sigma, 408727) per µg of DNA transfected. After 48 h, the medium was collected, run through a 0.45 µm filter (Thermo Fisher Scientific, 09-740-106) and stored in aliquots at −80 °C.

### Generation of cell lines with stable expression of PANK2 or PANK4

Cells were infected with lentivirus 24 h after plating at 50% density in medium containing 5 μg ml^−1^ polybrene (Sigma-Aldrich, 107689). Cells were subsequently selected in either 1 μg ml^−1^ puromycin (TLCV2) or 500 µg ml^−1^ G418 (Geneticin; pLUIN).

### Heavy-isotope-labelled metabolite labelling and treatments of cultured cells

Cells (600,000–700,000) were plated in 6 cm dishes in standard growth medium such that cells were approximately 75% confluent after 24 h. Three replicate plates for metabolomics and two replicate plates for protein lysates were plated, treated and collected in parallel.

#### ^13^C_3_^15^N_1_-vitamin B5 continuous labelling

VB5-free DMEM was custom formulated based on standard DMEM without pyruvate (Thermo Fisher Scientific/Gibco, 11965) but also without glucose, glutamine and VB5 (custom production by US Biological Life Sciences). Custom DMEM without supplements was used for washes, custom DMEM supplemented with 5 mM glucose, 1 mM glutamine and 3 μM unlabelled VB5 was used for serum/growth factor deprivations, and custom DMEM supplemented with 5 mM glucose, 1 mM glutamine and 3 μM ^13^C_3_^15^N_1_-VB5 was used for tracing. The day after plating (~30 h), cells were washed and then incubated in serum/growth-factor-deprivation medium for 18 h. Fresh serum/growth-factor-deprivation medium was placed on cells for an additional 2 h before washing and incubating cells in tracing medium with ^13^C_3_^15^N_1_-VB5 for 3 h (unless the time point is noted otherwise). Medium changes and treatments were staggered 15 min apart between treatment groups to achieve consistent treatment and collection times.

#### ^13^C_3_^15^N_1_-VB5 pulse-chase labelling

The pulse: serum/growth-factor-deprived cells were first incubated for 4 h with custom DMEM supplemented with 5 mM glucose, 1 mM glutamine and 3 μM ^13^C_3_^15^N_1_-VB5 (the pulse). Cells were then were washed and incubated for 1 h in custom DMEM supplemented with 5 mM glucose and 1 mM glutamine but lacking VB5 to deplete intracellular pools of unincorporated ^13^C_3_^15^N_1_-VB5. The chase: finally, cells were washed in custom DMEM and incubated for 3 h in custom DMEM supplemented with 5 mM glucose and 1 mM glutamine, and either with 3 μM ^13^C_3_^15^N_1_-VB5 or without VB5, and either with 100 nM insulin or vehicle.

#### ^13^C_3_^15^N_1_-cysteine labelling

For heavy-isotope-labelled ^13^C_3_^15^N_1_-cysteine tracing, treatment medium was prepared from DMEM formulated with 25 mM glucose but without glutamine, methionine, cysteine and cystine (Thermo Fisher Scientific/Gibco, 21013024) that was supplemented with 4 mM glutamine and 100 μM ^13^C_3_^15^N_1_-cysteine (Cambridge Isotope Laboratories, CNLM-3871-H). The plating and treatments were the same as for ^13^C_3_^15^N_1_-VB5 tracing described above except for the medium and supplements used.

### Growth factor and inhibitor treatments

#### Insulin/growth factors

For signalling experiments, serum/growth-factor-deprived cells (18 h) were treated with insulin or growth factors for the indicated time periods. Final concentrations and stocks were as follows: insulin (Thermo Fisher Scientific/Gibco, A11382II), final concentration, 100 nM; stock concentration, 100 μM (dissolved in 0.1 N HCl then diluted in water to final concentration); IGF-1 (Thermo Fisher Scientific, 291-G1-01M), final concentration, 50 ng ml^−1^; stock, 100 µg ml^−1^ (in 0.1% (w/v) BSA/PBS); and EGF (R&D Systems, 236-EG-01M), final concentration, 50 ng ml^−1^; stock, 100 µg ml^−1^ (in 0.1% (w/v) BSA/PBS). Stocks were prepared under sterile conditions, aliquoted and stored at −80 °C. Initiation of growth factor stimulation and isotopic tracing as described above were typically concurrent unless otherwise described.

#### PI3K and AKT kinase inhibitors

The small-molecule kinase inhibitors used included the PI3K catalytic inhibitors GDC-0941 (Cayman Chemical, 11600)^44^ and GDC-0032 (Selleck Chemicals, S7103)^45^ and BYL-719 (Active Biochem, A-1214)^46^, the AKT catalytic inhibitor GDC-0068 (Selleck Chemicals, S2808)^47^, and the AKT allosteric inhibitor MK-2206 (Cayman Chemical, 1032350-13-2)^48^. Note that AKT catalytic inhibitors are known to increase AKT phosphorylation on threonine 308 and serine 473 by stabilizing a conformation in which these phosphorylated residues are inaccessible to phosphatases while, at the same time, fully inhibiting AKT kinase activity and phosphorylation of downstream substrates^49^. Inhibitor stocks were dissolved in DMSO at 10 mM under sterile conditions, aliquoted and stored at −80 °C. Aliquots were freeze–thawed no more than five times. Insulin/growth factor treatments were preceded by 15 min of pretreatment with DMSO (Thermo Fisher Scientific, BP231-100) or a small-molecule inhibitor dissolved in DMSO. The inhibitors or DMSO were also present in the medium during labelling.

#### ACLY inhibitor

The specific ATP citrate lyase (ACLY) inhibitor NDI-091143 was previously described^18^. Stocks were dissolved in DMSO at 20 mM under sterile conditions, aliquoted and stored at −80 °C. Working concentrations were 15–20 μM.

#### PANK inhibitor

The pantothenate kinase inhibitor (Millipore-Sigma, 537983) is equivalent to ‘compound 7’ described in ref. ^30^. PANK inhibitor stocks were dissolved in DMSO at 10 mM under sterile conditions, aliquoted and stored at −80 °C. Aliquots were freeze–thawed no more than five times. For pantothenate kinase inhibitor treatments, after serum/growth factor deprivation (18 h) as described above, cells were changed into custom VB5-free DMEM supplemented with 5 mM glucose, 1 mM glutamine and no VB5 for 2 h before treatment. Cells were pretreated with the indicated amounts of inhibitor for 15 min and then changed into custom DMEM supplemented with 5 mM glucose, 1 mM glutamine and 100 nM ^13^C_3_^15^N_1_-VB5 plus inhibitor for 3 h.

### Metabolomics and heavy-isotope-labelled metabolite tracing

#### Metabolite extractions from cultured cells

The volumes described here are for 6 cm cell culture dishes unless otherwise noted. Each treatment group within an experiment consisted of five replicate dishes grown, treated and collected in parallel with three dishes for metabolite extraction and two dishes for protein lysates.

#### 80% methanol/water extraction

For unlabelled polar metabolomics analyses, cells were lysed on dry ice in 80% HPLC-grade methanol/20% HPLC-grade water with no ammonium acetate and processed for metabolomics as described below.

#### 80% methanol/water with 1 mM ammonium acetate extraction

For analysis of polar metabolites and acetyl-CoA, the addition of ammonium acetate was found to improve the acetyl-CoA signal in 80% methanol extracts. Cells were washed briefly with 4 ml ice-cold 1× PBS to remove extracellular metabolites and cellular debris. Metabolites (including VB5, CoA and acetyl-CoA) were extracted in 1.7 ml of 4 °C 80% (v/v) HPLC-grade methanol (Sigma-Aldrich, 34860)/20% HPLC-grade water (Sigma-Aldrich, 270733) with 1 mM ammonium acetate (Sigma-Aldrich, 431311) added immediately before use. Extracts were scraped into 2 ml tubes and centrifuged at 21,000*g* for 10 min at 4 °C to sediment protein and other insoluble material. Supernatants were transferred to 50 ml conical tubes for drying.

#### 10% trichloroacetic acid extraction

For analysis of CoA, acetyl-CoA and other short-chain acyl-CoAs (but not polar metabolites), cells were washed briefly with 4 ml 4 °C 1× PBS to remove extracellular metabolites and cellular debris. Metabolites (including CoA and short-chain acyl-CoAs) were extracted in 1.7 ml of freshly prepared in 4 °C 10% (w/v) trichloroacetic acid (Sigma-Aldrich, T6399) in HPLC-grade water. Extracts were scraped into 2 ml tubes and centrifuged at 21,000*g* for 10 min at 4 °C to sediment protein and other insoluble material. Supernatants were then passed over an Oasis HLB reversed-phase sorbent column (pre-activated with methanol and pre-equilibrated with water) (Waters, WAT094226), washed with HPLC-grade water and eluted with 2 × 750 µl of HPLC-grade methanol with 25 mM ammonium acetate via gravity flow at room temperature (columns were prepared and washes/elutions carried out according to manufacturer’s instructions). Eluates were collected in 50 ml conical tubes for drying. Adapted from previously described methods^12,50^.

#### Sample drying and storage

All samples were dried in 50 ml conical tubes under nitrogen gas at room temperature (typically 2–3 h) using an Organomation 24-position N-EVAP nitrogen evaporator, and dried pellets were stored at −80 °C (samples were always dried down prior to storage).

#### LC–MS/MS metabolomics analysis

On the day of sample analysis, dried metabolites in 50 ml tubes (from 6 cm dishes) were resuspended in 30 μl HPLC-grade water (Sigma-Aldrich), transferred to 1.5 ml tubes, centrifuged at 21,000*g* for 2 min at 4 °C and the supernatant was transferred to a fresh 1.5 ml tube. For each sample, half was submitted immediately for MS analysis while the remaining sample was snap-frozen in liquid nitrogen and stored at −80 °C as a backup. For each sample, 5 μl was injected and analysed using a hybrid 5500 or 6500 QTRAP triple quadrupole mass spectrometer (AB/SCIEX) coupled to a Prominence UFLC HPLC system (Shimadzu). The HPLC system used hydrophilic interaction chromatography (HILIC) and a 4.6 mm inner diameter × 10 cm Amide XBridge column (Waters) at 400 μl min^−1^. Gradients were run starting from 85% buffer B (HPLC-grade acetonitrile) to 42% B from 0–5 min; 42% B to 0% B from 5–16 min; 0% B was held from 16–24 min; 0% B to 85% B from 24–25 min; 85% B was held for 7 min to re-equilibrate the column. Buffer A comprised 20 mM ammonium hydroxide/20 mM ammonium acetate (pH 9.0) in 95:5 water:acetonitrile. Metabolites delivered to the mass spectrometer were analysed using selected reaction monitoring (SRM) performed with positive/negative ion polarity switching. Electrospray ionization (ESI) source voltage was +4,950 V in positive ion mode and −4,500 V in negative ion mode. The dwell time was 3 ms per SRM transition and the total cycle time was 1.39 s. Approximately 10–14 data points were acquired per detected metabolite. Peak areas from the total ion current for each metabolite were integrated using MultiQuant v.3.0 (AB/SCIEX). Metabolite total ion counts for a given transition were normalized to protein content of matched lysates for each treatment group, and treatment replicates were scaled around their replicate group means to normalize for run order effects between replicate groups^51^. For the unlabelled, targeted metabolomics screen, 283 endogenous water-soluble metabolites were targeted, some in both positive and negative ion mode, for a total of 304 SRM transitions^13,52^. A significant signal is not detected for all metabolites in every experiment. For heavy-isotope-labelled metabolite tracing, SRMs were adapted from previous studies^12,50,52^ with optimization of collision energies and the most reliable Q3 product ion if needed. Key SRMs were validated in-house using purified metabolites and/or extracts from cells that had or had not been exposed to labelled metabolites as controls. See Supplementary Table 1 (MS-MS transition) for more information.

#### Analysis of unlabelled polar metabolites altered between *PANK4-*KO cell lines with stable re-expression of vector, wild-type or *PANK4*^D623A^

MCF10A *AKT1*^*p.E17K/+*^, *PANK4*-KO reconstituted with empty vector (vector), wild-type *PANK4* or *PANK4*^*D623A*^ cells were grown for 48 h in serum-free custom VB5-free DMEM supplemented with 10% KnockOut Serum Replacement (Thermo Fisher Scientific/Gibco, 10828028), 10 µg ml^−1^ insulin, 0.5 mg ml^−1^ hydrocortisone, 20 ng ml^−1^ EGF, 100 ng ml^−1^ cholera toxin, 10 mM glucose, 2 mM glutamine and in the absence of VB5. After 24 h, cells were washed for 1 h in this same medium. After 48 h, cells were seeded into 6 cm plates in the same medium but containing 1 µM VB5. Three replicate plates were seeded for metabolite collection, two for protein collection and three plates that contained medium but no cells as blank controls. Metabolites were collected in 80% (v/v) methanol/water as above at both 48 h and 72 h after plating. For statistical analysis, metabolites were filtered out if the maximum intensity for all treatment group averages was either less than 1.5× the intensity of the average of three blank samples, or was less than 10,000. Metabolite intensities were normalized to relative protein concentrations. Metabolites that were significantly altered in a PANK4-phosphatase-activity-dependent manner were identified by correlation with CoA levels between cell lines (PatternHunter, MetaboAnalyst v.4.0, 2–1–2 for vector–PANK4(WT)–PANK4(D623A), false-discovery rate < 0.1). Of these hits, metabolites altered in the same direction at both time points were analysed using one-way ANOVA (false-discovery rate < 0.1, MetaboAnalyst 4.0). Significantly altered metabolites were visualized by heat map as the fold change relative to vector for each respective time point.

### Unlabelled lipidomics analysis

#### Cell culture conditions and collection of samples for lipidomics

In each experiment, four cell lines were compared with five replicate sample plates per cell line in parallel (three plates for collecting lipids and two plates for collecting protein lysates). The cell lines included MCF10A *AKT1*^*p.E17K/+*^ and MCF10A *AKT1*^*p.E17K*^ *PANK4-*KO cells stably reconstituted with empty vector or full-length untagged wild-type *PANK4*, *PANK4*^*D623A*^, *PANK4*^*D659A*^, *PANK4*^*T406A*^ or *PANK4*^*T406E*^. For comparison of vector, wild-type-, D623A- and D659A-expressing cell lines, cells were seeded in 6 cm plates such that they were around 50% confluent the next day, in serum-free custom VB5-free DMEM supplemented with 10% KnockOut Serum Replacement (Thermo Fisher Scientific/Gibco, 10828028), 10 µg ml^−1^ insulin, 0.5 mg ml^−1^ hydrocortisone, 20 ng ml^−1^ EGF, 100 ng ml^−1^ cholera toxin, 10 mM glucose, 2 mM glutamine and 3 μM VB5. For comparison of *PANK4-*KO cell lines expressing vector, wild-type *PANK4*, *PANK4*^*T406A*^ or *PANK4*^*T406E*^ cells were grown for 48 h in the above-described medium in the absence of VB5. After 24 h, cells were washed for 1 h in this same medium. After 48 h, cells were seeded into 6 cm plates in the same medium but containing 1 µM VB5. The three replicate plates for lipidomics and two replicate plates for protein lysates were seeded, treated and collected in parallel. Three plates with medium but without cells served as blanks. Plates were changed into fresh medium 24 h after seeding and lipids were extracted 48 h after seeding. For the collection of protein lysates, cells were lysed in NP40 lysis buffer as described in the ‘Immunoblotting (western blotting)’ section. The following lipid collection procedure was adapted from previous methods^53^. For extraction of lipids, plates were placed on wet ice and quickly washed with 4 °C 1× PBS. Then, 1.5 ml of 80% (v/v) HPLC-grade methanol in HPLC grade water at 4 °C was added to the dishes and cells were scraped into prechilled 15 ml glass vials (Cole-Parmer, EW-34534-10) on wet ice. Once all samples were collected, all of the remaining steps were performed at room temperature. For each sample, 4 ml of HPLC-grade MTBE (methyl tert-butyl ether; Sigma Aldrich, 34875) was added to each glass vial with a glass pipette (Cole-Parmer, EW-2555-3-16), and all of the vials were vortexed for 1 min before rocking for 15 min. To induce phase separation, 0.7 ml of HPLC-grade water was added to each vial and the vials were vortexed for 1 min before centrifugation at 1,000*g* for 10 min. The final ratio for the MTBE:methanol:water mixture was 10:3:2.5 and the final volume was 6.2 ml. Then, 3.5 ml of the upper (organic) layer from each vial was transferred to a fresh glass vial and dried under nitrogen gas. Dried metabolites were stored at −80 °C until the day of analysis. Immediately before LC–MS/MS analysis, dried samples were resuspended in 35 μl of 1:1 HPLC-grade isopropanol:methanol and centrifuged briefly to collect the liquid in the bottom of the glass tube. The samples were then transferred to a 1.7 ml polypropylene microcentrifuge tube, and centrifuged at 21,000*g* for 2 min. For each sample, half of the supernatant was transferred to a glass autosampler vial for LC–MS/MS analysis, and the other half was stored in a second glass autosampler vial at −80 °C as a backup.

#### MS analysis for unlabelled lipidomics

For each resuspended sample, 5 μl was injected into an Agilent 1100-Thermo QExactive Plus-Thermo LipidSearch lipidomics platform. A reverse-phase Cadenza 150 mm × 2 mm C_18_ column with 3 μm particle size (Imtakt) heated to 40 °C at 240 μl min^−1^ was used with a 1100 quaternary pump HPLC with room temperature autosampler (Agilent). Lipids were eluted over a 22 min gradient from 32% B buffer (90% isopropyl alcohol (IPA)/10% acetonitrile (ACN)/10 mM ammonium formate/0.1 formic acid) to 97% B buffer. A buffer consisted of 59.9% ACN/40% water/10 mM ammonium formate/0.1% formic acid. Lipids were analysed using a high-resolution hybrid QExactive Plus Orbitrap mass spectrometer (Thermo Fisher Scientific) in DDA mode (Top 8) using positive/negative ion polarity switching. DDA data were acquired from *m*/*z* 225 to 1,450 in MS1 mode and the resolution was set to 70,000 for MS1 and 35,000 for MS2. MS1 and MS2 target values were set to 5 × 10^5^ and 1 × 10^6^, respectively.

#### Raw lipidomics data analysis

Raw lipidomics data were analysed for both identification and relative quantification using LipidSearch v.4.1.30 software (Thermo Fisher Scientific) using an internal database of more than 20 main lipid classes and more than 80 subclasses^53^.

#### Analysis of lipids altered between *PANK4-*KO cell lines with stable re-expression of vector, wild-type *PANK4*, *PANK4*^*D623A*^ or *PANK4*^*D659A*^

Three independent replicate experiments were performed with four cell lines compared in each experiment and three replicate plates per cell line for collecting lipids. Lipid species with any cellular sample values lower than the average of the blanks were filtered out. Remaining lipid species were normalized to the relative protein concentration of the respective treatment group. For each independent experiment, lipid species with differential abundance between cell lines were identified by one-way ANOVA analysis (MetaboAnalyst v.4.0, false-discovery rate < 0.1). For each lipid that passed this criterion in two out of three independent experiments, the fold change in that lipid between vector-expressing cells and the other three cell lines was calculated, and the values from each independent experiment were averaged. In the analysis, replicates with ‘N/A’ readings were treated as ‘no value’. Using an absolute fold change cut-off (vector-expressing cells versus *PANK4*-WT expressing cells) of 25%, relative changes were tested for correlation to CoA levels in these cell lines (Fig. 4c) (PatternHunter, MetaboAnalyst v.4.0; *k*-means clustering by sample; pattern: 2–1–2–2 for cell lines empty vector–*PANK* WT–*PANK4*^*D623A*^–*PANK4*^*D659A*^; false-discovery rate < 0.1)^54^. Significantly correlated hits were visualized by heat map as average log_2_-transformed fold change relative to vector-expressing cells.

#### Analysis of lipids altered between *PANK4*-KO cell lines with stable re-expression of vector, wild-type *PANK4*, *PANK4*^*T406A*^ or *PANK4*^*T406E*^

Three replicates between the four cell lines were analysed as above. After analysis using one-way ANOVA, significantly altered lipids (false-discovery rate < 0.1, fold change > 25%) were tested for correlation to CoA levels as described above (PatternHunter, MetaboAnalyst 4.0; pattern: 3–2–1–2 for cell lines empty vector–*PANK*-WT–*PANK4*^*T406A*^–*PANK4*^*T406E*^; false-discovery rate < 0.1). Significantly correlated lipids with even-chain fatty acids were visualized by heat map as the average fold change relative to the row mean of each lipid.

### ^13^C tracing from ^13^C_6_-glucose into lipids lipidomics analysis

#### Cell culture conditions, labelling and collection of samples for labelled lipidomics

Cells were grown for 48 h in custom DMEM in the presence of 10% Knockout Serum Replacement, MCF10A medium growth factors, 10 mM glucose, 2 mM glutamine and in the absence of VB5. Three medium-only samples were plated at the same time as blanks, and one set of samples was grown in unlabelled glucose as a control. After 24 h, cells were washed for 1 h in this same medium. After 48 h, cells were seeded into 6 cm plates in the same medium but containing 1 µM VB5. After 48 h in the presence of VB5, cells were washed in 2 ml of custom medium, and the medium was changed into the same medium but containing 10 mM unlabelled glucose or 10 mM ^13^C_6_-glucose (Cambridge Isotope Laboratories, CLM-1396). After 16 h of glucose labelling, lipid extracts were collected as described above. At the time of submission, a quality control sample dilution curve was included with pooled samples diluted at 1×, 0.3× and 0.1× in 50% isopropanol:50% methanol.

#### Mass spectrometry for ^13^C-labelled lipidomics

The non-polar lipid samples were resuspended in 35 μl of 1:1 HPLC-grade isopropanol:methanol before LC–MS/MS analysis, 5 μl was injected. A Cadenza 150 mm × 2 mm 3 μm C_18_ column (Imtakt) heated to 37 °C at 200 μl min^−1^ was used with a 1200 quaternary pump HPLC with room temperature autosampler (Agilent). Lipids were eluted over a 25 min. Gradient from 32% B buffer (90% IPA/10% ACN/10 mM ammonium formate/0.1 formic acid) to 97% B. A buffer consisted of 59.9% ACN/40% water/10 mM ammonium formate/0.1% formic acid. Lipids were analysed using a high-resolution hybrid QExactive HF Orbitrap mass spectrometer (Thermo Fisher Scientific) in DDA mode (top 8) using positive/negative-ion polarity switching. DDA data were acquired from *m*/*z* 225–1,450 in MS1 mode and the resolution was set to 70,000 for MS1 and 35,000 for MS2. MS1 and MS2 target values were set to 5 × 10^5^ and 1 × 10^6^, respectively. Lipidomic data were analysed for both identification and relative quantification using Elements v.2.0 (Proteome Software) using the National Institute of Standards and Technology (NIST) v.17 and Human Metabolome Database (HMDB) v.4.0 small-molecule spectral databases. The Elements software was also used to trace the incorporation of ^13^C isotopologues into identified lipids as described previously^55^.

#### Statistical analysis of labelled lipidomics data

Lipid intensities were normalized to the treatment group protein content. Features were filtered out that had minimum values across experiment samples below the average of three blank medium-only samples. Next, features measured within a linear range were selected for by a correlation coefficient of *r* ≥ 0.9 between the measured lipid intensities and the expected intensities in the quality control sample dilution curve (1×, 0.3×, 0.1×). Overall, lipid isotopologues of *M* + 3 and above were enriched in labelled samples relative to unlabelled samples, so features that met this criterion were analysed by *t*-test between labelled vector and *PANK4*-WT samples. Lipid species with even-chain fatty acids that were significantly different between treatment groups (false discovery rate < 0.1, fold change > 20%) were visualized by heat map as the fold change relative to the average of the vector samples.

### Seahorse XF Cell Mito Stress Test

Cells were grown for 48 h in custom DMEM in the presence of 10% Knockout Serum Replacement, MCF10A medium growth factors, 10 mM glucose, 2 mM glutamine and in the absence of VB5. After 24 h, cells were washed for 1 h in this same medium. After 48 h, cells were passaged into the same medium, but containing 1 µM VB5. After 48 h in the presence of VB5, cells were seeded at 7,500 cells per well in an Agilent XF 96-well plate (Agilent, 101085-004). The Seahorse XF Cell Mito Stress Test was performed on a Seahorse XFe96 Analyzer according to the manufacturer’s instructions, and cells were treated with 1 µM oligomycin, 1 µM FCCP and 500 nM rotenone/antimycin. Oxygen consumption values were normalized to cellular protein content as measured by DC assay.

### Histone extraction

Cells were grown for 48 h in custom DMEM in the presence of 10% Knockout Serum Replacement, MCF10A medium growth factors, 10 mM glucose, 2 mM glutamine and in the absence of VB5. After 24 h, cells were washed for 1 h in this same medium. After 48 h, cells were seeded into 6 cm plates in the same medium but containing 1 µM VB5. Two sets of plates were seeded in parallel, one to be lysed in hypotonic lysis buffer for acid extraction, and one to be lysed in NP40 lysis buffer as described above. After 48 h in the presence of VB5, the medium was changed. Then, 16 h later, cells were put on wet ice and the medium was aspirated. Cells were washed in 4 °C 1× PBS and then lysed in hypotonic lysis buffer (0.05% NP40, 10 mM HEPES pH 7.4, 10 mM KCl) with protease inhibitor cocktail and scraped into 1.5 ml tubes. Lysates were rocked at 4 °C for 20 min and subsequently centrifuged at 16,000*g* for 10 min. Supernatant was saved for protein concentration assay and western blot analysis. Pellets were resuspended in 0.2 N HCl and rocked at 4 °C for 20 min and then centrifuged at 16,000*g* for 10 min. Supernatant was transferred to a fresh tube and neutralized with 1 M Tris-HCl pH 8.0. Acid extracted lysates were normalized to protein levels from non-acid extracted protein lysates. Lysates were also normalized based on total histone H3 levels in an initial immunoblot.

### Radioactive ^14^C-VB5 labelling of cells

For ^14^C-VB5 labelling experiments, cells were plated to around 70% density in medium with full serum, with three replicates for radioactive labelling measurements, one plate as a radioactivity blank and two plates for protein lysates. All of the plates were treated in parallel as described for heavy-isotope-labelled metabolite tracing experiments except for the use of 100 nCi ml^−1^ ^14^C-VB5 (specific activity, 50–60 mCi mmol^−1^; American Radiolabeled Chemicals, ARC 0653). Plates for protein lysates were collected after 4 h. After 4 h of incubation with ^14^C-VB5, radiolabelled cells were washed with custom DMEM and then chased for 1 h with custom DMEM containing 5 mM glucose, 1 mM glutamine and no pantothenate. The purpose of this 1 h incubation was to remove free radiolabelled VB5 that had not been incorporated into CoA. Cells were washed with room temperature 1× PBS, collected in 1 ml Solvable Aqueous Based Solubilizer (0.4 M sodium hydroxide in water, plus three specialized surfactants; PerkinElmer, 6NE9100) and incubated at 60 °C for 1 h. Cell extracts were transferred to glass scintillation vials and mixed with 10 ml Ultima Gold Scintillation Fluid (PerkinElmer, 6013321), before measuring disintegrations per minute of ^14^C on a scintillation counter (Beckman LS 6500). All medium changes and treatments were staggered 15 min apart between treatment arms of a given experiment to achieve consistent treatment and collection times.

### Immunoblotting (western blotting)

Cells were washed briefly with 4 °C 1× PBS (Boston BioProducts, BM-220) and collected on wet ice in 4 °C NP40 lysis buffer (1× stock stored at 4 °C: 40 mM HEPES, pH 7.5 (Thermo Fisher Scientific/Gibco, 15630080); 120 mM NaCl (Sigma-Aldrich, S7653); 1 mM EDTA, pH 8.0 (Sigma-Aldrich, E6758); 1% IGEPAL CA-630 (NP40) (Sigma-Aldrich, I3021); 10 mM sodium pyrophosphate tetrabasic (Sigma-Aldrich, S6422); 10 mM β-glycerophosphate (Sigma-Aldrich, G9422S); 50 mM sodium fluoride (Thermo Fisher Scientific, S25547); 5% (v/v) glycerol (Thermo Fisher Scientific, G33–500)) with 1:100 protease inhibitor cocktail (104 mM AEBSF, 80 μM aprotinin, 4 mM bestatin, 1.4 mM E-64, 2 mM leupeptin and 1.5 mM pepstatin A) (Sigma-Aldrich, P8340)) added on the day of use. The plates were scraped into 1.5 ml tubes and centrifuged at 15,000*g* for 10 min at 4 °C. The supernatants were transferred to new tubes and protein content was measured by DC protein assay (based on Lowry assay; Bio-Rad, 5000116). Lysates were normalized for protein concentration and Laemmli sample buffer was added for a final 1× concentration (1×: 50 mM Tris base, pH 6.8 (Thermo Fisher Scientific, BP152); 2% (w/v) SDS (Thermo Fisher Scientific, NC0755841); 5% (v/v) β-mercaptoethanol (Sigma-Aldrich, M3148); 5% (v/v) glycerol (Thermo Fisher Scientific, G33–500); 0.01% (w/v) bromophenol blue; (6× stock aliquots stored at −20 °C and freeze–thawed no more than three times)). Western blot samples were run by SDS–PAGE on 10% Tris-Glycine gels in Tris-glycine running buffer (25 mM Tris Base; 0.1% (w/v) SDS; 250 mM glycine) or 4–12% Bis-Tris gradient gels (Thermo Fisher Scientific, NP0329BOX) in MOPS running buffer (Thermo Fisher Scientific, NP000102). Proteins were transferred to PVDF membranes (Millipore-Sigma, IPVH00010) at a constant 300 mA for 90 min at 4 °C in Tris-glycine transfer buffer (10× Transfer (Electro) Blotting Buffer (Boston BioProducts, BP-190) diluted to 1× (25 mM Tris-base, 192 mM glycine, pH 8.5) in water with 10% methanol (v/v)). Membranes were briefly washed in 1× TBST buffer (20× Tris-buffered saline with Tween-20 (Boston BioProducts, IBB-870) diluted to 1× (50 mM Tris, 150 mM NaCl, 0.05% Tween-20, pH 7.6) in water) and blocked in 5% (w/v) non-fat dry milk (Thermo Fisher Scientific, NC9022655) in TBST for 1 h at room temperature. Membranes were briefly washed in TBST and incubated in primary antibody diluted in 5% (w/v) fatty-acid-free bovine serum albumin (BSA) (Boston BioProducts, P-753) in TBST with 0.01% (w/v) sodium azide (Thermo Fisher Scientific) and rocking at 4 °C overnight. Membranes were washed three times for 10 min in TBST at room temperature and incubated in horseradish peroxidase (HRP)-conjugated secondary antibody diluted in 5% (w/v) milk in TBST for 1 h, with rocking at room temperature. Membranes were washed three times for 10 min in TBST at room temperature and incubated in Clarity Western ECL Substrate (Bio-Rad, 1705061) or Clarity MAX Western ECL Substrate (Bio-Rad, 1705062) for 2–5 min. The chemiluminescence signal was detected using film (Thomas Scientific, F-BX810) or a Bio-Rad ChemiDoc Imaging System (Bio-Rad, 17001401).

See Supplementary Table 1 (immunoblot antibodies) for further information regarding antibodies and Supplementary Fig. 1 for raw immunoblot film scans.

### Immunoprecipitation analysis of PANK1/2/4

All steps were carried out on wet ice or at 4 °C. Cells in 10 cm dishes were lysed in 700 μl of NP40 lysis buffer on ice and scraped into 1.5 ml tubes. Lysates were centrifuged at 15,000*g* for 10 min at 4 °C, the supernatants were transferred to a new tube, centrifuged a second time at 15,000*g* for 5 min at 4 °C and the cleared supernatants were transferred to a new tube. Protein concentrations were normalized and the samples were brought up to the same volume (at least 600 µl) in NP40 lysis buffer. A total of 100 μl of lysate was saved for immunoblotting. The remaining lysate (at least 500 μl) was incubated for 2 h with 1 μg of immunoprecipitation antibody, rocking at 4 °C. Immunoprecipitation antibodies were bound to either protein A/G agarose beads (Thermo Fisher Scientific, 20421) or protein G Dynabeads (Thermo Fisher Scientific, 10004D). Protein A/G agarose beads were washed twice with bead wash buffer (40 mM HEPES, pH 7.5 and 120 mM NaCl), centrifuged at no more than 2,500*g* for 1 min and resuspended in a 1:1 slurry in bead wash buffer. Protein G Dynabeads were similarly washed and resuspended by pelleting on a DynaMag-2 Magnet (Thermo Fisher Scientific, 12321-D). Then, 20 µl of resuspended beads were added to each immunoprecipitation sample for an additional 2 h with rocking at 4 °C. Three washes were performed with 500 µl NP40 lysis buffer and beads were centrifuged at 2,500*g* or pelleted using a magnet, followed by removal of all of the supernatant above the settled beads and resuspension in 1× sample buffer. The samples were heated at 95 °C for 5 min and stored at −80 °C. See Supplementary Table 1 (IP antibodies) for further information.

### In vitro AKT kinase assay

For in vitro AKT kinase assays, immunoprecipitations using lysates from 10 cm dishes were performed as described above until the final resuspension step. After three washes in NP40 lysis buffer, two further washes were performed in 500 µl kinase reaction buffer (20 mM HEPES, pH 7.5 (Thermo Fisher Scientific/Gibco, 15630080), 10 mM MgCl_2_ (Sigma-Aldrich), 0.5 mM EGTA (Sigma-Aldrich)). All of the supernatant was removed and the beads were resuspended in 20 µl of kinase reaction buffer +100 µM ATP (Sigma-Aldrich) and 1 mM DTT (Thermo Fisher Scientific) with or without 250 ng of purified GST–AKT1 (Sigma-Aldrich). The samples were incubated for 30 min at 32 °C with periodic agitation and put on ice to stop the reaction. A portion of each sample supernatant was saved and sample buffer was added to 1× final concentration to each supernatant. The beads were resuspended in 1× Laemmli sample buffer. Samples were heated at 95 °C for 5 min and stored at −80 °C. For immunoblotting, approximately one-quarter of each immunoprecipitation sample was loaded per SDS–PAGE gel.

### Synthesis of 4′-phosphopantothenate

4′-Phosphopantothenate was prepared by D.B. in the laboratory of J.L.M. See Supplementary Fig. 2 for a diagram of reaction steps for 4′-phosphopantothenate synthesis.

#### General

Unless otherwise noted, all reagents were obtained from commercial suppliers and used without further purification. Yields of all reactions refer to the purified products. Silica chromatography was carried out in the indicated solvent system using prepacked silica gel cartridges for use on the Teledyne ISCO Purification System. ^1^H and ^31^P NMR spectra were acquired on the Bruker 500 MHz instrument operating at 500 MHz for ^1^H, 126 MHz for ^13^C and 202 MHz for ^31^P. ^31^P chemical shifts are reported relative to triphenylphosphine oxide at δ 24.7 as an external standard.

#### Synthesis

Phospho-d-pantothenate was synthesized in three steps from commercially available sodium d-pantothenate as previously described with minor modifications^56^.

#### d-Pantothenate benzyl ester

Sodium d-pantothenate (0.500 g, 2.07 mmol) was suspended in dimethylformamide (7.5 ml) and benzyl bromide (0.246 ml, 2.07 mmol) was added. The reaction mixture stirred for 18 h at 70 °C. Solvent was removed under reduced pressure and the resultant residue was dissolved in ethyl acetate (50 ml) and washed with brine (three times with 10 ml). The organic layer was dried over anhydrous sodium sulfate, filtered and condensed under reduced pressure. The crude material was purified by silica flash chromatography (3:1 to 1:0 ethyl acetate:hexanes) to yield d-pantothenate benzyl ester as a colourless oil (0.510 g, 80% yield).

#### 4-Phospho-d-pantothenate tribenzyl ester

Dibenzyl phosphite (1.09 ml, 4.95 mmol) was dissolved in toluene (6.6 ml) and *N*-chlorosuccinimide (0.661 g, 4.95 mmol) was added. The reaction mixture was stirred at 22 °C for 16 h and then filtered into a dried flask. In a separate dried flask, d-pantothenate benzyl ester (0.51 g, 1.65 mmol) was azeotropically dried with anhydrous pyridine (2 × 5 ml) before being dissolved in anhydrous pyridine (7.4 ml) and cooled to −40 °C (dry ice/acetonitrile). The dibenzylchlorophosphate solution was added dropwise to the d-pantothenate benzyl ester solution at −40 °C. After 2 h at −40 °C, the reaction mixture was maintained at −20 °C for 16 h, after which it was warmed to 22 °C and quenched with water (3 ml). Volatiles were removed under reduced pressure and the resulting residue was dissolved in ethyl acetate (25 ml) and washed sequentially with 1 M H_2_SO_4_ (twice with 5 ml) and saturated NaHCO_3_ (twice with 5 ml). The organic layer was dried over anhydrous sodium sulfate, filtered, and condensed under reduced pressure. Purification by silica flash chromatography (2:1 to 1:0 ethyl acetate:hexanes) yielded 4-phospho-d-pantothenate tribenzyl ester as a colourless oil (0.617 g, 66% yield). ^1^H NMR (500 MHz, DMSO) δ 7.86 (t, *J* = 5.9 Hz, 1H), 7.42–7.29 (m, 14H), 5.70 (d, *J* = 5.7 Hz, 1H), 5.07 (s, 2H), 5.03 (s, 2H), 5.02 (s, 2H), 3.91 (dd, *J* = 9.3, 4.5 Hz, 1H), 3.79 (dd, *J* = 9.4, 4.4 Hz, 1H), 3.68 (d, *J* = 5.7 Hz, 1H), 3.40 (dq, *J* = 13.2, 6.7 Hz, 1H), 3.28 (dt, *J* = 12.9, 6.5 Hz, 1H), 2.54 (p, *J* = 6.9 Hz, 2H), 0.84 (s, 3H), 0.81 (s, 3H). ^13^C NMR (126 MHz, DMSO) δ 172.04, 171.27, 136.18, 136.12, 136.05, 128.46, 128.42, 128.31, 128.01, 127.98, 127.77, 73.84, 72.96 (d, *J* = 6.1 Hz), 68.40 (d, *J* = 5.5 Hz), 65.57, 38.51 (d, *J* = 8.1 Hz), 34.28, 33.77, 20.39, 19.68. ^31^P NMR (202 MHz, DMSO) δ −0.42.

#### 4-Phospho-d-pantothenic acid

4-Phospho-d-pantothenate tribenzyl ester (0.200 g, 0.35 mmol) was dissolved in methanol (2 ml). The flask was flushed with N_2_ and then Pd/C (10%, 10 mg) was added. The reaction mixture was sparged with H_2_ for 5 min then stirred vigorously under H_2_ (1 atm) for 2 h. The reaction mixture was filtered through a 0.45 µm PTFE filter to remove the catalyst and the filtrate was condensed under reduced pressure. The crude product was purified by reverse-phase flash chromatography (C_18_, 0 to 50% acetonitrile in water) to yield 4-phospho-d-pantothenic acid as an amorphous solid (0.065 g, 62% yield) after lyophilization. ^1^H NMR (500 MHz, D_2_O) δ 4.04 (s, 1H), 3.83 (dd, *J* = 9.6, 4.0 Hz, 1H), 3.63 (dd, *J* = 9.7, 4.9 Hz, 1H), 3.52 (td, *J* = 6.5, 3.2 Hz, 2H), 2.64 (t, *J* = 6.5 Hz, 2H), 0.98 (s, 3H), 0.91 (s, 3H). ^13^C NMR (126 MHz, D_2_O) δ 176.13, 174.83, 74.40, 71.37, 52.31, 38.26 (d, *J* = 7.8 Hz), 34.73, 33.51, 20.57, 18.54. ^31^P NMR (202 MHz, D_2_O) δ 0.24. ESI calculated for C_9_H_17_NO_8_P^−^ [M – H]^−^ 298.1, found 298.1.

### Synthesis of 4′-phosphopantetheine

4′-Phosphopantetheine was prepared by T.T.Z. in the laboratory of J.L.M. 4′-Phosphopantetheine was prepared from pantetheine through enzymatic phosphorylation using *Staphylococcus aureus* pantothenate kinase and ATP using an adaptation of a previously described protocol^57^. Pantetheine was freshly reduced by sodium borohydride before the enzymatic reaction^58^. The 4′-phosphopantetheine product was purified by reverse-phase HPLC to yield a solid with NMR and LC–MS spectra that were identical to previous reports^36,59^.

### In vitro PANK4 phosphatase assay

Phosphatase assays were adapted from previous methods^33^. Note that an isolated DUF89 phosphatase domain of PANK4 (C-terminally His-tagged) was used in ref. ^33^, whereas in this study we used full-length PANK4 (N-terminally Flag-tagged). HEK293T cells were transiently transfected with pcDNA3.1 constructs containing empty vector or Flag-tagged PANK4 for 48 h and lysed as described in the ‘Immunoprecipitation analysis of PANK1/2/4’ section. Anti-flag M2 agarose beads (Millipore-Sigma, A2220) were added to lysates for 4 h with rocking at 4 °C to immunopurify Flag–PANK4. The beads were washed twice in 4 °C 1% NP40 lysis buffer with protease inhibitors but without phosphatase inhibitors, and twice in 4 °C phosphatase reaction buffer (50 mM Tris-HCL pH 7.5, 100 mM NaCl, 0.5 mM Co^2+^). Excess buffer was removed from above the settled beads before reaction buffer with substrate was added (see below). Immunoprecipitations were run on SDS–PAGE gels alongside BSA standards and gels were Coomassie stained, imaged and quantified using ImageJ to determine Flag–PANK4 protein concentrations.

#### Reaction using PNPP substrate

Flag-tag immunopurifications on M2 agarose beads were incubated with 20 μl of 50 mM para-nitrophenyl phosphate (PNPP) (New England Biolabs, P0757L) in reaction buffer at 30 °C for 30 min with regular mixing of beads and solution. The reaction was stopped by mixing 10 μl of reaction supernatant with 90 μl of 1 N NaOH. Absorbance at 405 nm was measured on a spectrophotometer.

#### Reaction using phosphopantothenate or phosphopantetheine substrate

Flag-tag immunopurifications on M2 agarose beads were incubated with 20 μl of water as a control or 2 mM 4′-phosphopantothenate or 4′-phosphopantetheine in reaction buffer at 30 °C for 5 min. Inorganic phosphate released during the reaction was quantified using a malachite green phosphate detection kit (R&D Systems, DY996). Absorbance at 620 nm was measured on a spectrophotometer.

### 2D proliferation assay

Cells were cultured for 24 h in serum-free custom VB5-free DMEM supplemented with 10% KnockOut Serum Replacement (Thermo Fisher Scientific/Gibco, 10828028), 10 µg ml^−1^ insulin, 0.5 mg ml^−1^ hydrocortisone, 20 ng ml^−1^ EGF, 100 ng ml^−1^ cholera toxin, 10 mM glucose, 2 mM glutamine and without VB5, before being seeded in the same medium at 7,500 cells per well in 24-well format. Serum, which contains variable concentrations of VB5, was replaced in the medium with KnockOut Serum Replacement, which does not contain VB5, to allow for precise control of VB5 concentrations. The next day (24 to 30 h later), cells were switched to custom DMEM supplemented with nutrients and growth factors as described above, plus 1 μM VB5. Cells were incubated for 4 days with a complete medium change on day 2. On days 0, 2 and 4, cells were fixed through addition of trichloroacetic acid directly to the medium to a final concentration of 8.33% (w/v). Total protein staining was performed by a sulforhodamine B assay^60^. Raw absorbance at 510 nm was converted to cell number using a standard curve created using MCF10A cells.

### 3D soft agar colony-formation assay

A 1.25% agar (Thermo Fisher Scientific, BP1423) stock was diluted to 0.625% in standard MCF10A growth medium (see the formula above in the ‘Cell lines and culture conditions’ section), and 2 ml per well on six-well plate was plated as the bottom agar layer. Cells were quickly seeded at 30,000 per well in six-well format, in 2.5 ml of 0.28% agar diluted in standard MCF10A growth medium. After two weeks, cells were stained with 0.01% crystal violet (Sigma-Aldrich, C6158) overnight and imaged on the Bio-Rad ChemiDoc Imaging System (Bio-Rad, 17001401). Colonies were counted manually using ImageJ.

### Mouse experiments

All animal experiments carried out at Beth Israel Deaconess Medical Center (BIDMC) were approved by and performed in accordance with the guidelines of the BIDMC Institutional Animal Care and Use Committee (IACUC) (animal protocol, 102–2015). Mice were maintained in a barrier facility with ventilated cage racks and an automatic watering system. Animal holding rooms were set to maintain a temperature of 70–72°F with 40–60% humidity, and rooms are maintained on a 12 h–12 h day–night cycle from 07:00 to 19:00. ES272 allograft tumour studies were approved by and performed in accordance with the guidelines of the Weill Cornell Medicine IACUC (animal protocol 2013-0116).

#### ES272 orthotopic mammary allograft tumour mouse model treated with BYL-719

The orthotopic allograft mammary tumour model experiment was performed by B.D.H. at Weill Cornell Medicine. The mouse mammary cancer cell line ES272^29^, which is also known as FR-1^61^, was previously derived from a mammary tumour arising in a transgenic mouse conditionally expressing mammary-restricted *Pik3ca*^*p.H1047R*^ (C57BL/6J background)^61^. The growth of ES272 allograft tumours has been shown to be sensitive to PI3K inhibitor treatments^29^. ES272 cells were cultured in DMEM plus 10% FBS before injection into mice. To generate orthotopic allograft mammary tumours, 1 × 10^6^ ES272 cells were suspended in serum-free medium, mixed 1:1 with Matrigel and injected into the fourth mammary gland of 8–10 week-old immunocompetent C57BL/6J mice. Once tumours reached a diameter of 0.65 cm, the mice were treated with 45 mg kg^−1^ of BYL-719 (Alpelisib) or vehicle (0.5% methylcellulose, 0.5% Tween-80) through oral gavage once per day for 10 days. Then 3 h after the final treatment, the mice were euthanized by CO_2_ asphyxiation followed by cervical dislocation. Tumours were then quickly excised, snap-frozen in liquid nitrogen and stored at −80 °C.

#### BYL-719 1 h treatment and muscle collection

This mouse experiment was performed at BIDMC in collaboration with the laboratory of J.G.C. BYL-719 (Alpelisib) (Chem Express, HM-206_014-20120614) was dissolved/suspended in vehicle (0.5% methylcellulose, 0.5% Tween-80) at 5 mg ml^−1^ using a bath sonicator and stored at 4 °C overnight before use. C57BL/6J male mice (Jackson Laboratories, 000664; aged 11–12 weeks) were fed ad libitum with standard chow and then administered 50 mg kg^−1^ BYL-719 or vehicle through oral gavage. Then, 1 h after treatment, the mice were anaesthetized with avertin and gastrocnemius muscle was collected, immediately snap-frozen in liquid nitrogen and stored at −80 °C.

#### PANK4 immunoprecipitation from tumours and muscle tissue

Snap-frozen mouse xenograft tumours and skeletal muscle were stored at −80 °C and then kept on dry ice until immediately before processing. Lysates were kept on wet ice. Samples were processed in prechilled (4 °C) NP40 lysis buffer with its standard complement of phosphatase inhibitors (see above) plus 1:50 HALT protease inhibitor cocktail (Thermo Fischer Scientific, 78429). The samples were homogenized in a gentleMACS Dissociator (Miltenyibiotec, 130-096-235) in gentleMACS M tubes (Miltenyibiotec, 130-096-335) on the protein M tube setting. Lysates were cleared by centrifuging the M tubes at 3,000*g* for 5 min, followed by subsequent centrifugation in 1.5 or 2 ml tubes at 20,000*g* for 10 min each until the lysates were cleared of insoluble debris. Lysate aliquots were diluted 1:5 or 1:10 before determining protein concentration by DC assay. All immunoprecipitation steps were performed as above, except that tissue lysates were incubated with antibody and beads in NP40 lysis buffer with 1:50 HALT protease inhibitor cocktail.

#### SUM159 orthotopic mammary xenograft tumour model

This mouse experiment was performed at BIDMC in collaboration with the laboratory of J.G.C. SUM159 cells were maintained in RPMI medium with 10% FBS. Cells tested negative for mycoplasma before injection. On the day of injection, 80% confluent cells were trypsinized, washed and counted. A total of 5 × 10^6^ cells per mouse were resuspended in 100 μl serum-free RPMI medium and placed onto wet ice. Cell suspensions were mixed at a 1:1 (v/v) ratio with Matrigel (Corning, 356230) and orthotopically injected into the fourth mammary fat pad of 6–8 week-old NCr nude female mice (Taconic Biosciences, NCRNU-F). Tumours were measured with callipers three times per week (length and width) for 50 days and tumour volumes were calculated as (width × width × length)/2. Mice were euthanized when the tumours reached either of two end points: a 750 mm^3^ volume or ulceration. The remaining mice were euthanized at the end of the study. Ten mice were injected per treatment group (one injection per mouse) and the maximum number tumours that progressed to an end point in any single group was four (in the *PANK4* knockout + empty vector group). Thus, the top four largest tumours in each treatment group were used for the analysis.

### Protein and gene accession numbers

The following human sequences were used for siRNA and gRNA design, protein sequence alignments, and expression in experiments: PANK1, PANK2, PANK3, PANK4. Phosphopantothenoylcysteine synthetase (PPCS), phosphopantothenoylcysteine decarboxylase (PPCDC), and coenzyme A synthase (COASY). A list of the accession codes is provided in Supplementary Table 1 (Human sequence references).

### Alignments of DUF89 domain catalytic aspartates

For amino acid alignments between PANK4 and other DUF89-domain-containing proteins, the species, proteins and corresponding sequence identifiers are as follows: *Homo sapiens* PANK4 (UniProt: Q9NVE7)*, Arabidopsis thaliana* At2g17340 (UniProt: Q949P3)*, Pyrococcus horikoshii* PH1575 (UniProt: O59272) and *Saccharomyces cerevisiae* YMR027W (UniProt: Q04371). The catalytic aspartates in the non-PANK4 proteins were identified previously^33^. Sequences were aligned using Clustal Omega.

### Statistical analyses

Experiments with two treatment groups were analysed using two-tailed Student’s *t*-tests (*α* = 0.05). Experiments with three or more treatment groups were analysed using one-way ANOVA with a post hoc Tukey test (*α* = 0.05). Experiments with three or more treatment groups and multiple metabolites compared as a group were analysed using two-way ANOVA with a post hoc Sidak test (*α* = 0.05). Data were processed with Excel v.16.46 (Microsoft), MetaboAnalyst v.4.0 and ImageJ v.1.50i (W. Rasband, NIH). Statistical analyses were performed within PRISM 8 graphing software (GraphPad). Biological replicates are individual plates of cells that were plated, treated, collected and analysed in parallel in a single experiment. Experiment replicates are independent experiments with average values of triplicates from each experiment graphed.

### Reporting summary

Further information on research design is available in the Nature Research Reporting Summary linked to this paper.

## Online content

Any methods, additional references, Nature Research reporting summaries, source data, extended data, supplementary information, acknowledgements, peer review information; details of author contributions and competing interests; and statements of data and code availability are available at 10.1038/s41586-022-04984-8.

## Supplementary information

Supplementary Fig. 1 Raw western blot film scans from all main and extended data figures.

Reporting Summary

Supplementary Fig. 2 Diagram of steps for 4′-phosphopantothenate synthesis.

Supplementary Table 1 Information regarding siRNA sequences, sgRNA sequences, cloning and sequencing primer sequences, human reference gene sequences, tandem MS transitions and antibodies.

## Data Availability

All data are available in the the Article and its Supplementary Information. PerkinElmer/Horizon Discovery cell lines and Addgene plasmids are under materials transfer agreements. Source data are provided with this paper.

## Source data

Source Data Extended Data Fig. 10
Source Data Fig. 4
