# Supplementary material for: PI3K drives the de novo synthesis of coenzyme A from vitamin B5
Source: Nature. 2022 Jul 27;608(7921):192–8. doi: 10.1038/s41586-022-04984-8 (PMC9352595; doi:10.1038/s41586-022-04984-8)

nature portfolio

https://doi.org/10.1038/s41586-022-04984-8

Supplementary information

PI3K drives the de novo synthesis of coenzyme A from vitamin B5

In the format provided by the authors and unedited

Raw immunoblots

Note: Blots for corresponding phosphorylated and total proteins from the same samples were run on separate gels

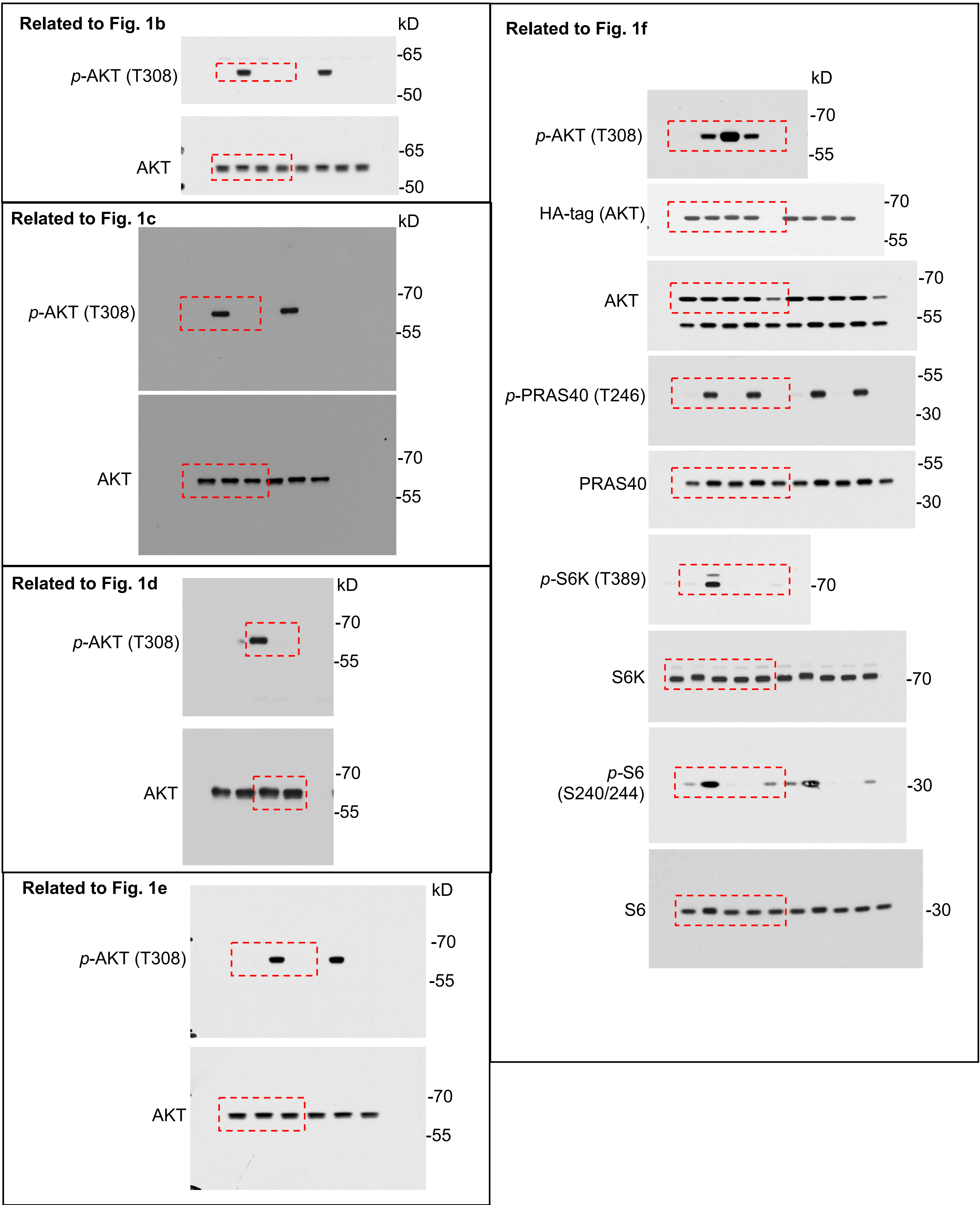

Related to Fig. 1g

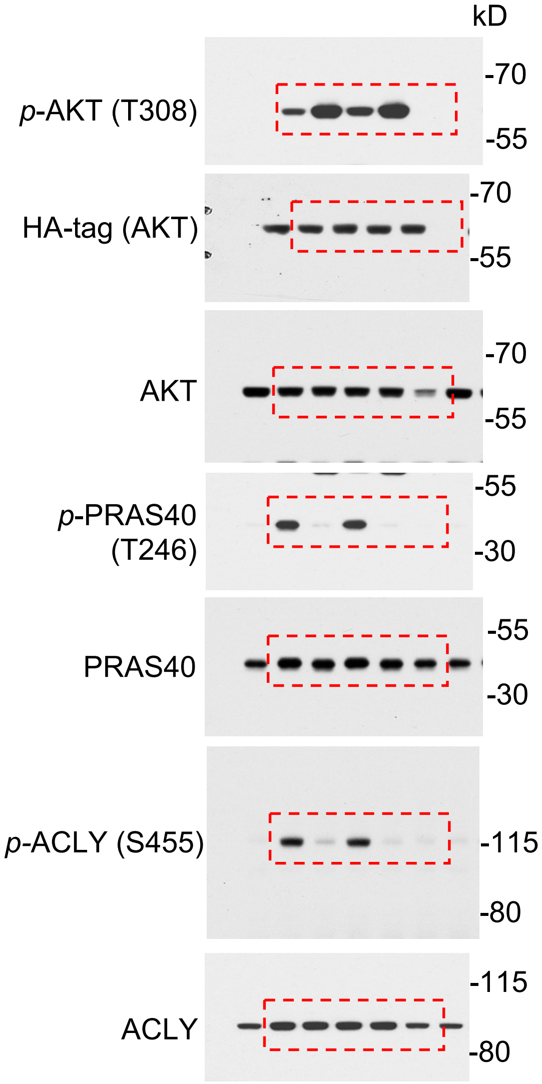

Related to Fig. 2c

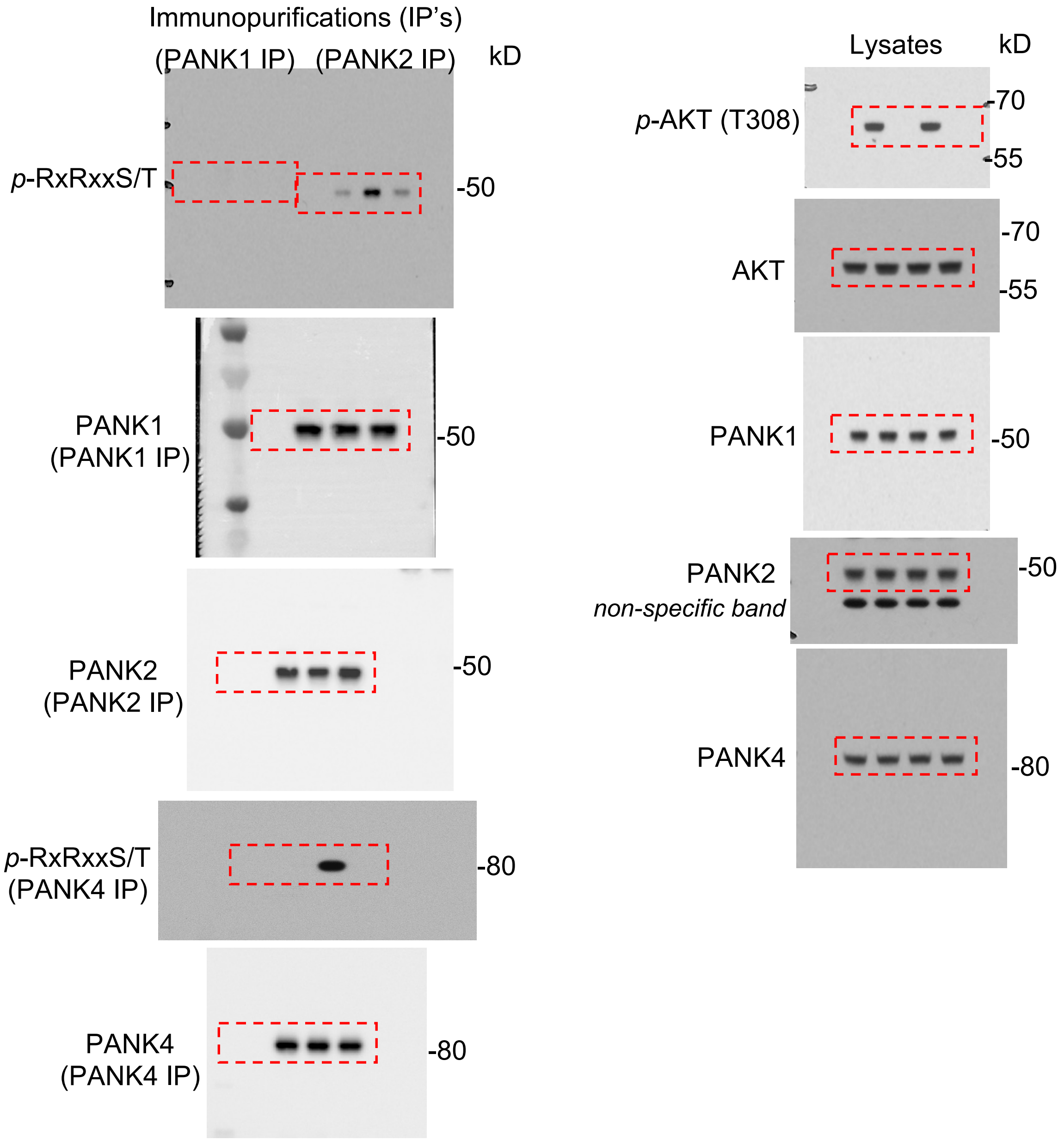

Related to Fig. 2d

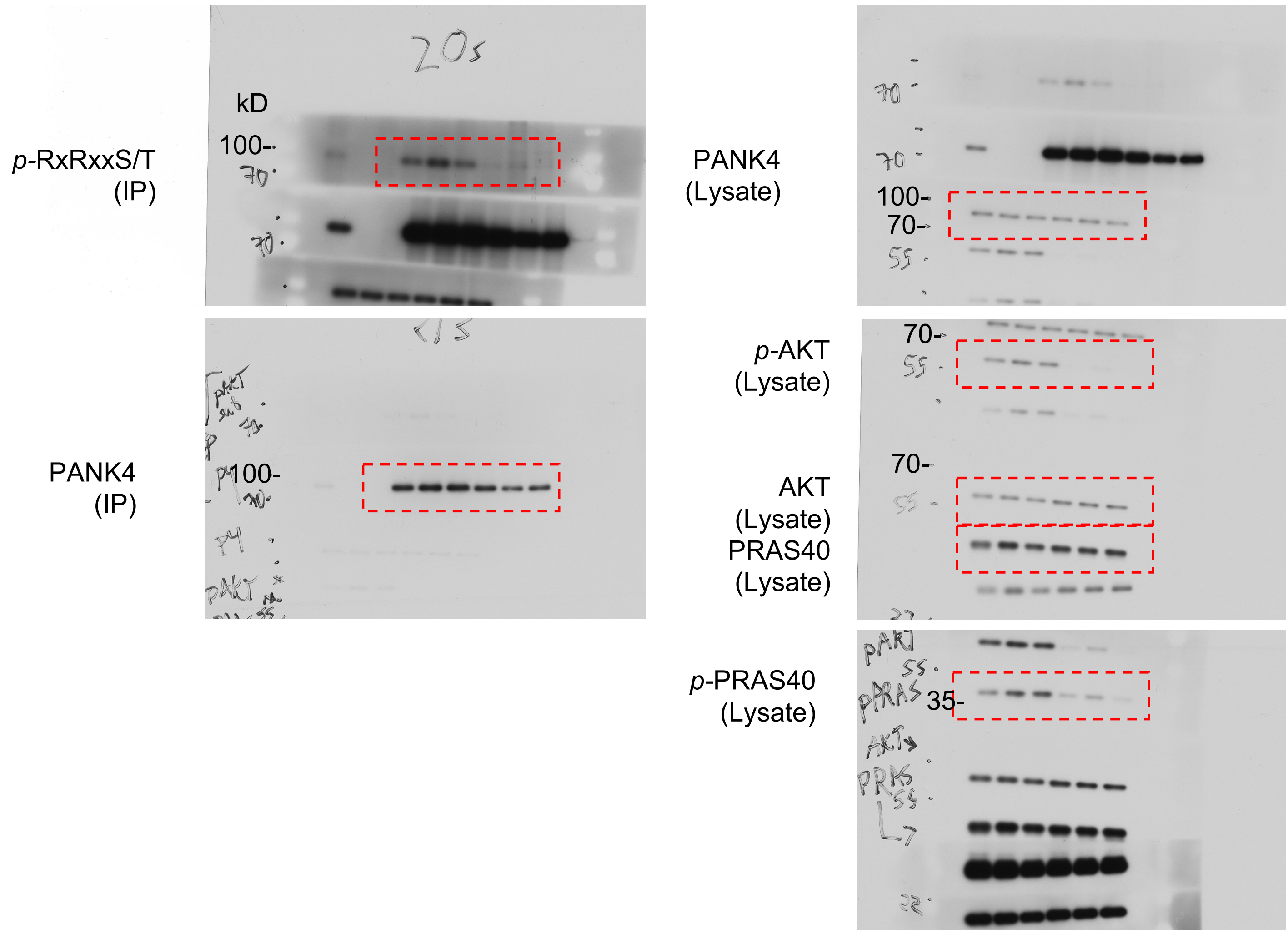

Related to Fig. 2e

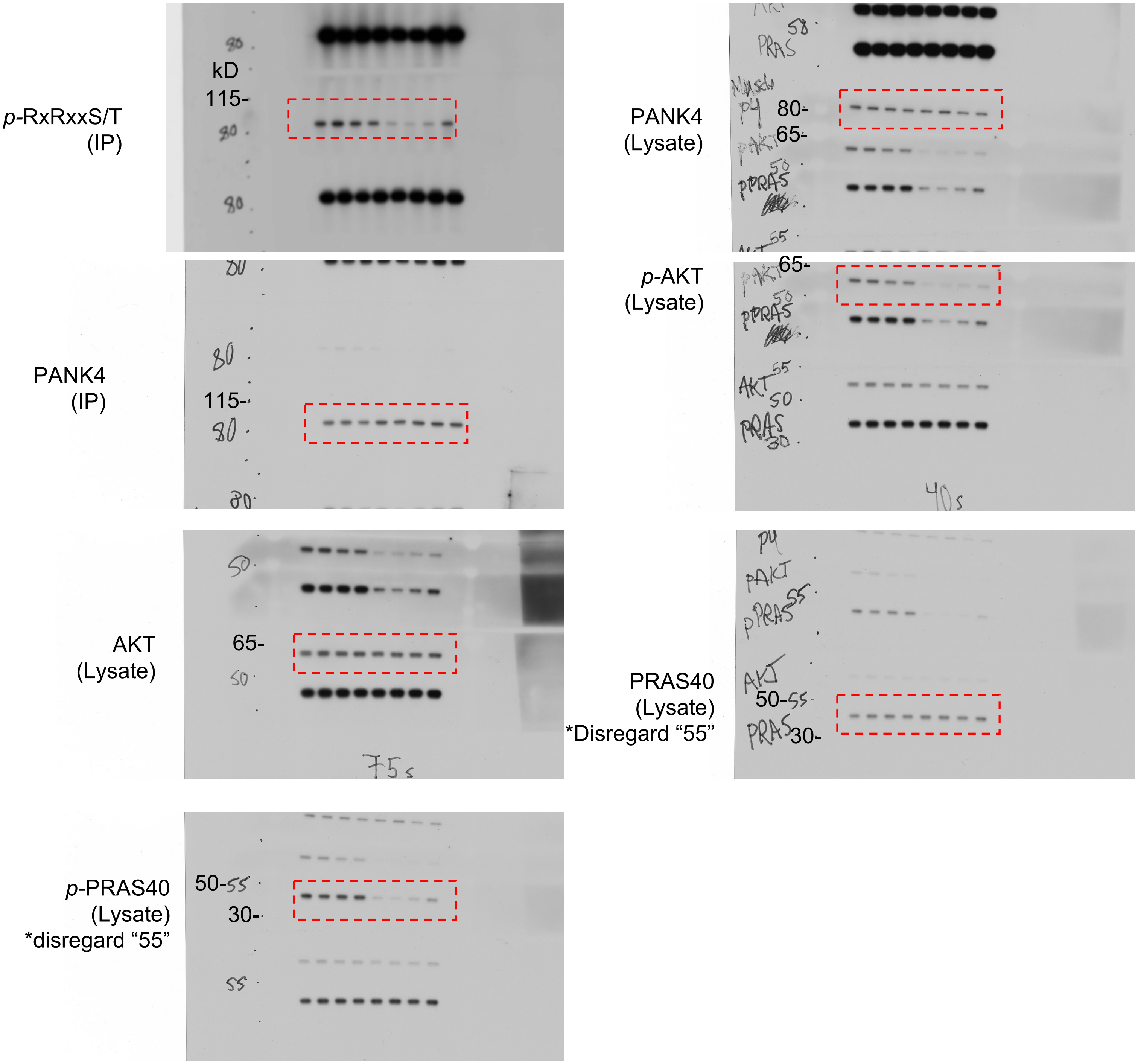

Related to Fig. 2f

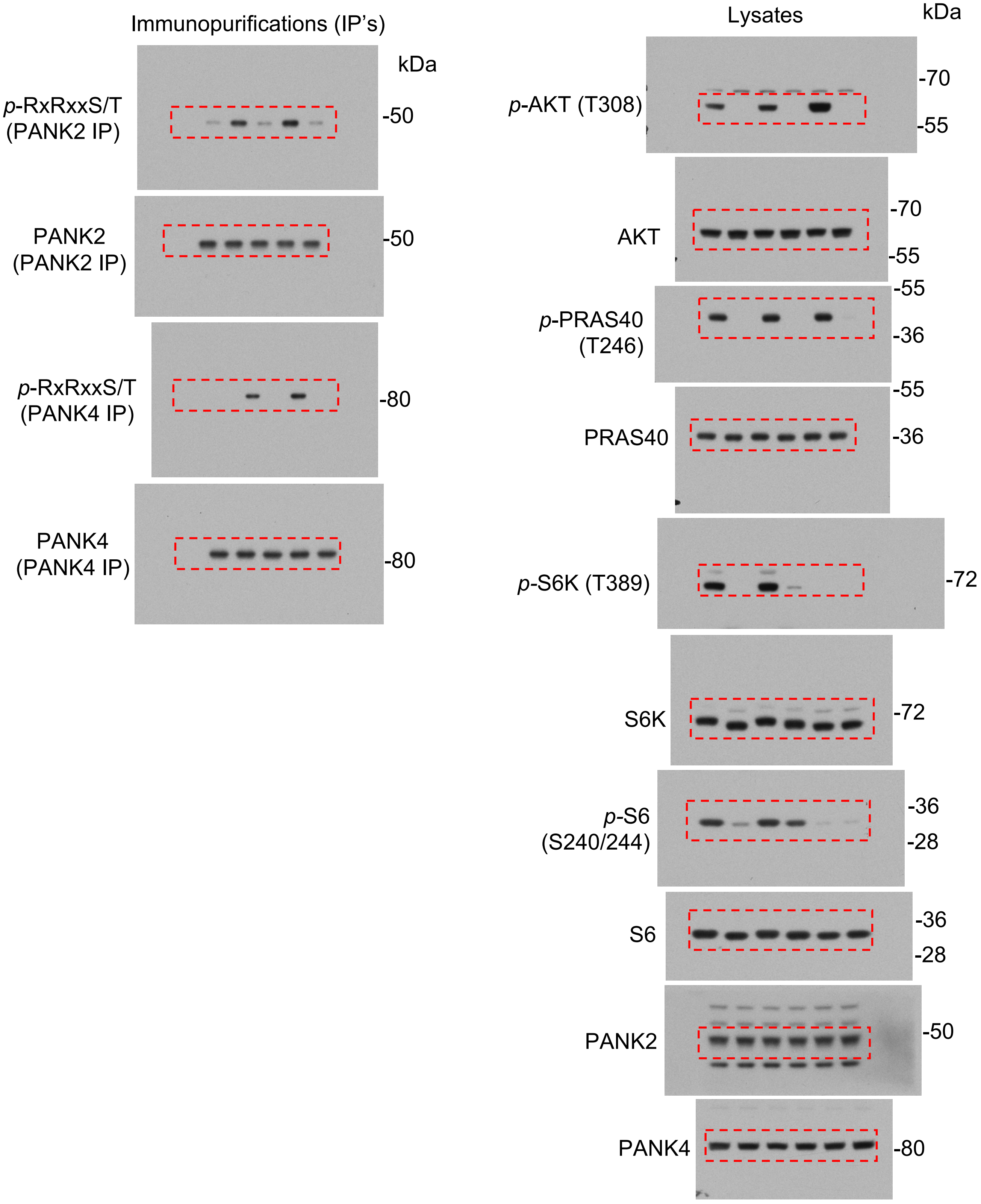

Related to Fig. 2g

p-RxRxxS/T
Light Exposure
PANK2 Mature Band
Expected at 48 kD

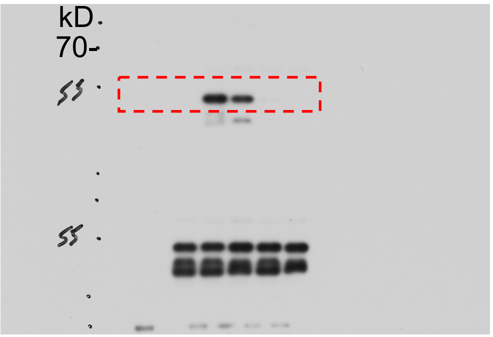

PANK2
PANK2 Mature Band
Expected at 48 kD

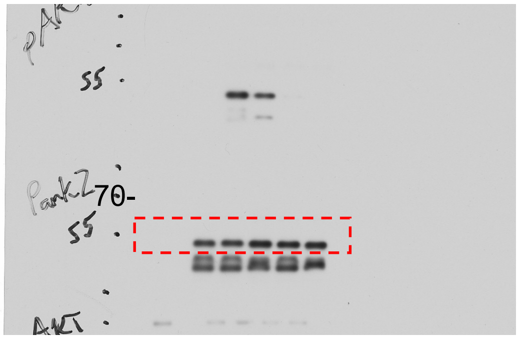

p-RxRxxS/T
Dark Exposure
PANK2 Mature Band
Expected at 48 kD

AKT
GST-AKT
Expected at 80Kd

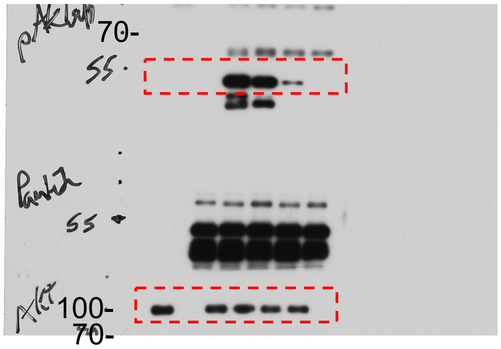

Related to Fig. 2h

p-RxRxxS/T

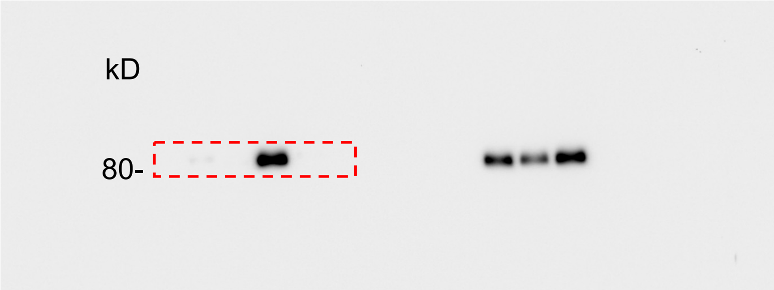

PANK4

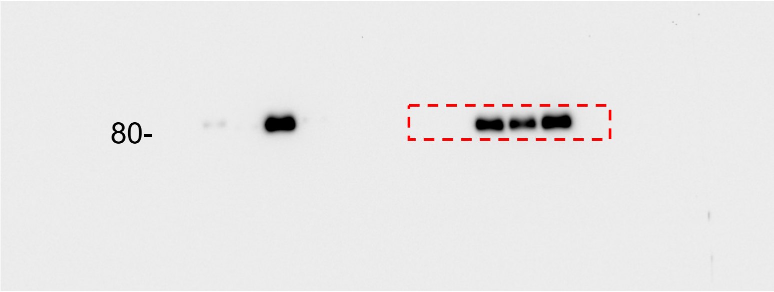

Related to Fig. 3a

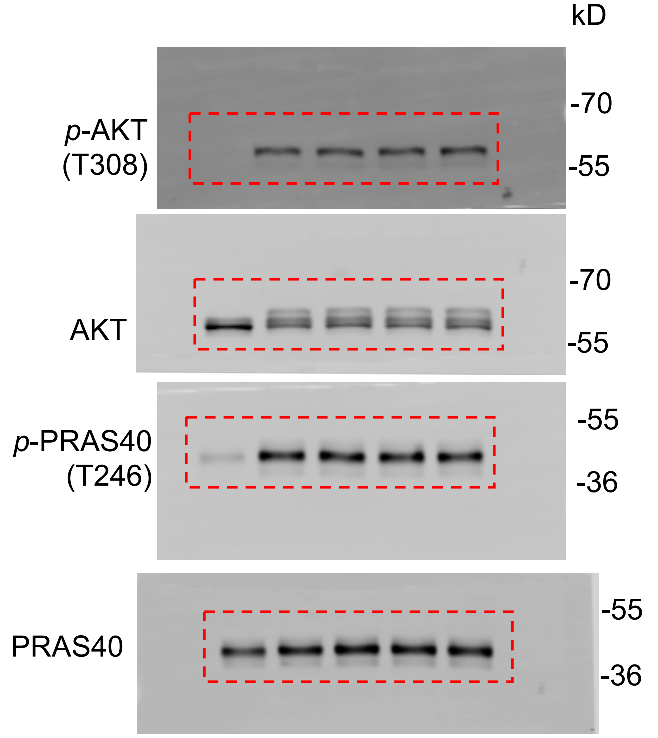

Related to Fig. 3b

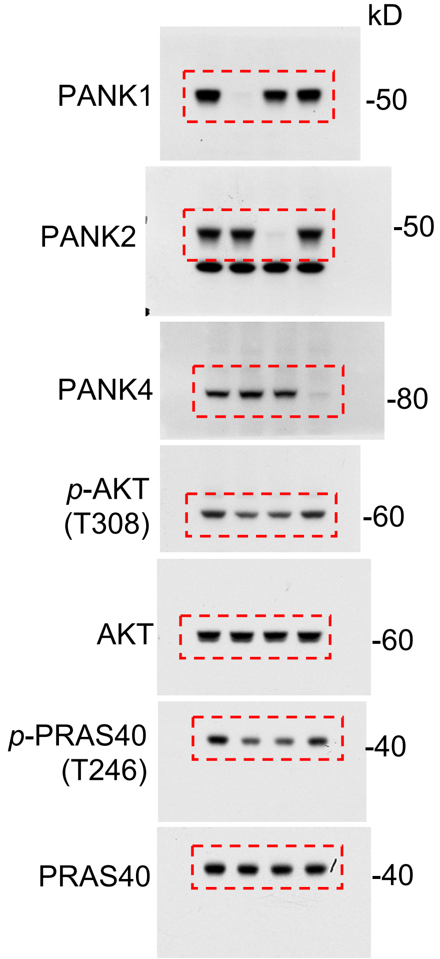

Related to Fig. 3c

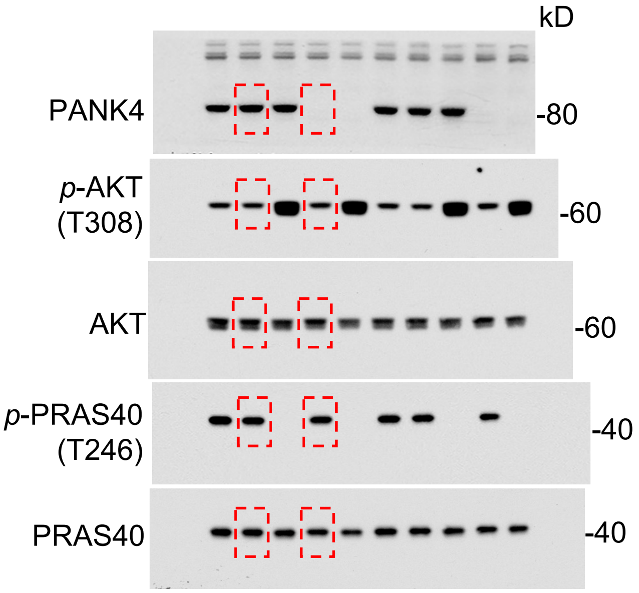

Related to Fig. 3d

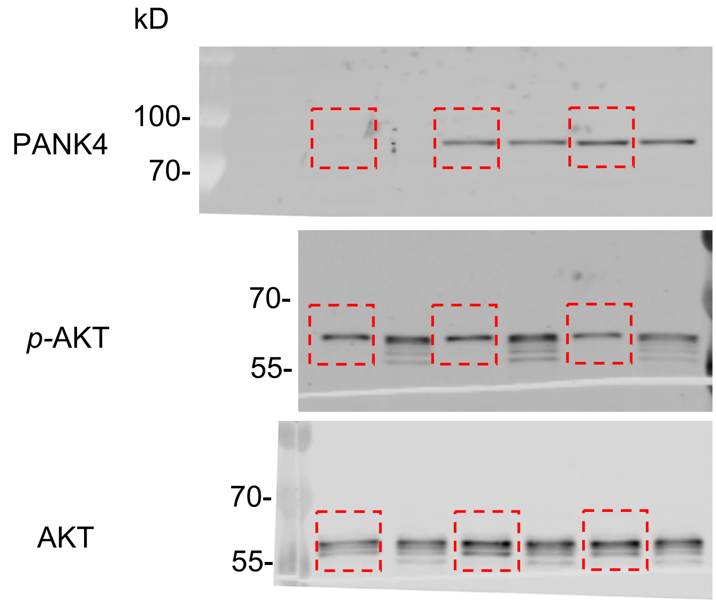

Bands from same SDS-PAGE gel and image.

Related to Fig. 3e

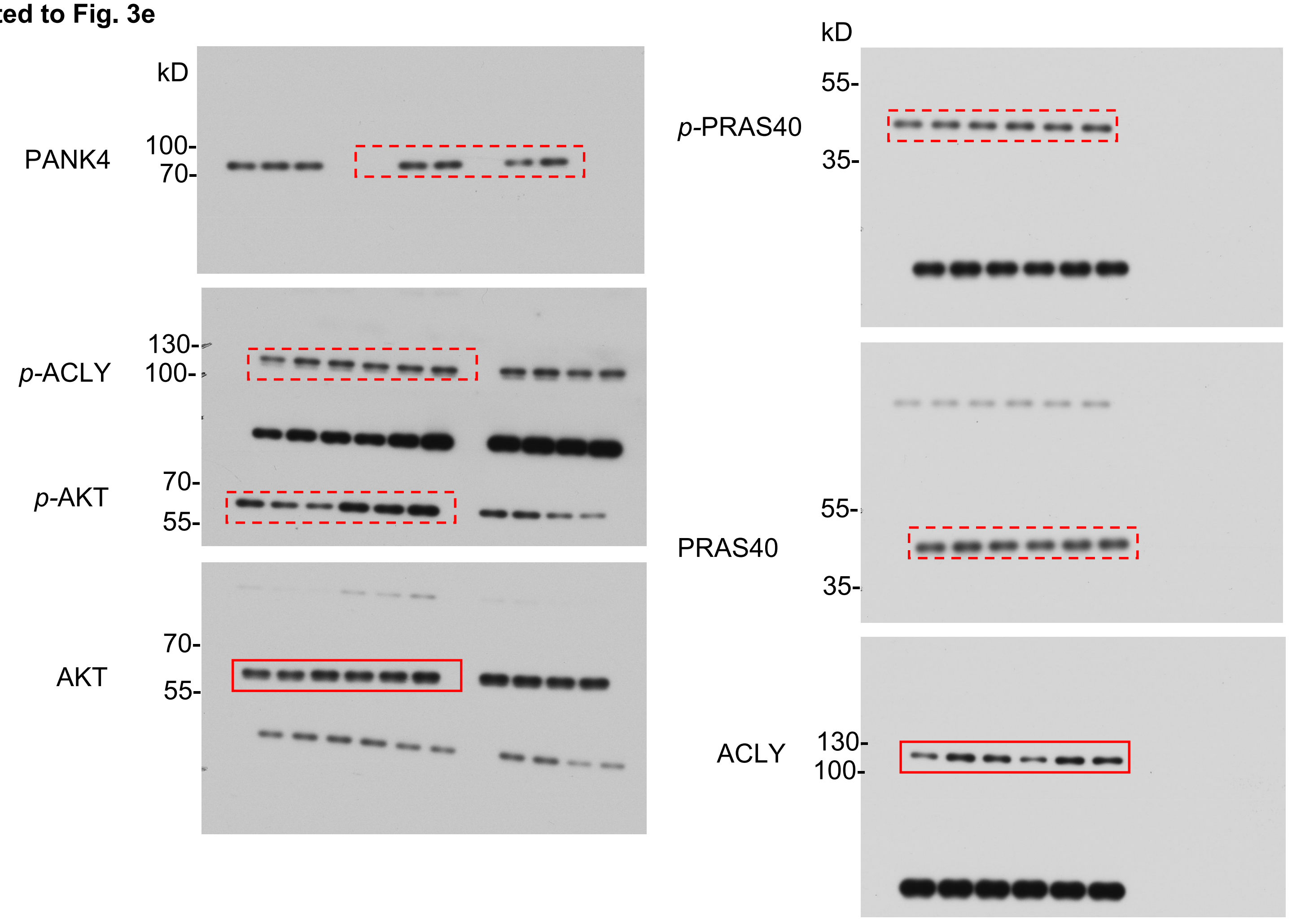

Related to Fig. 4b

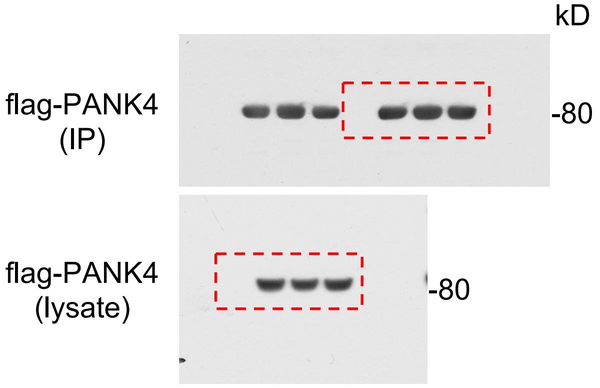

Related to Fig. 4d

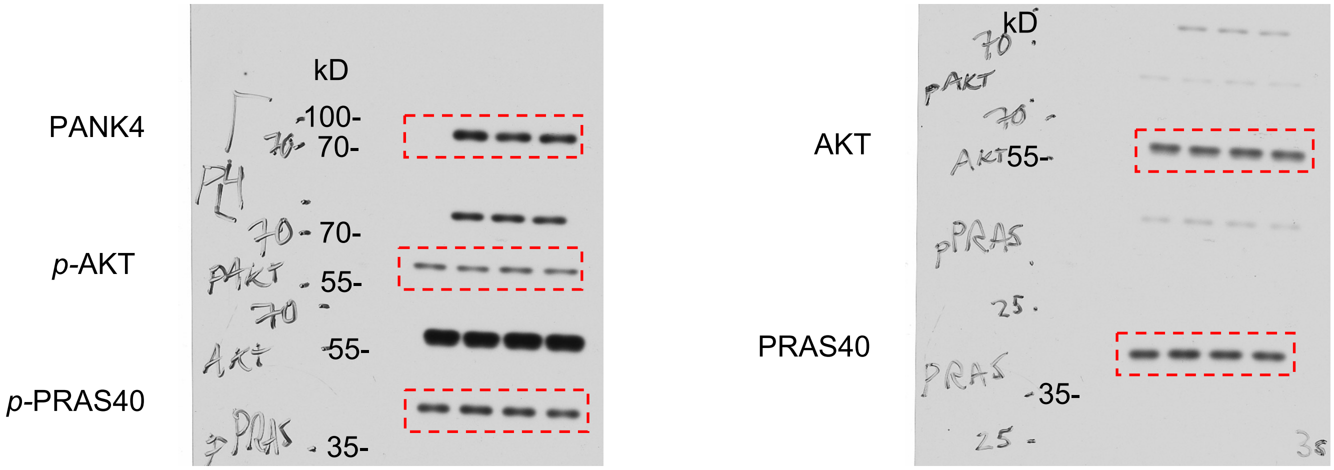

Related to Fig. 4j

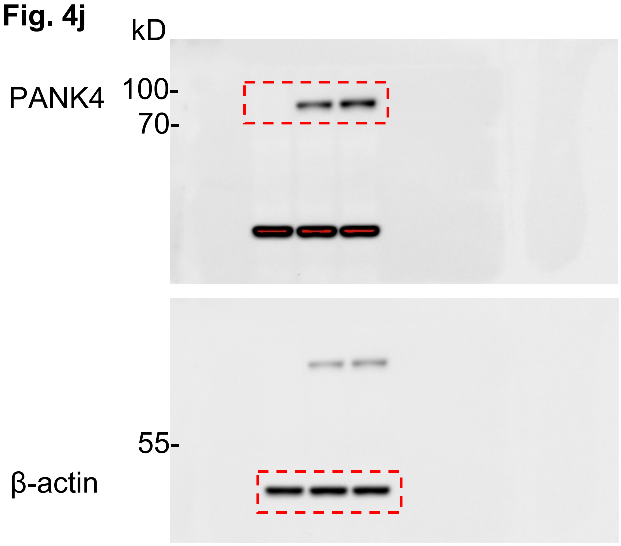

Related to Extended Data Fig. 1e

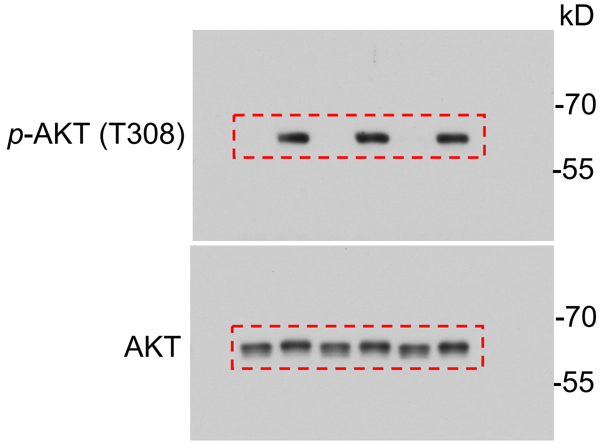

Related to Extended Data Fig. 2d

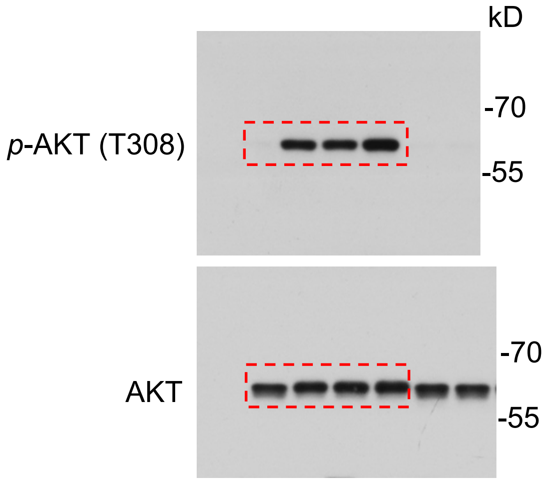

Related to Extended Data Fig. 2e

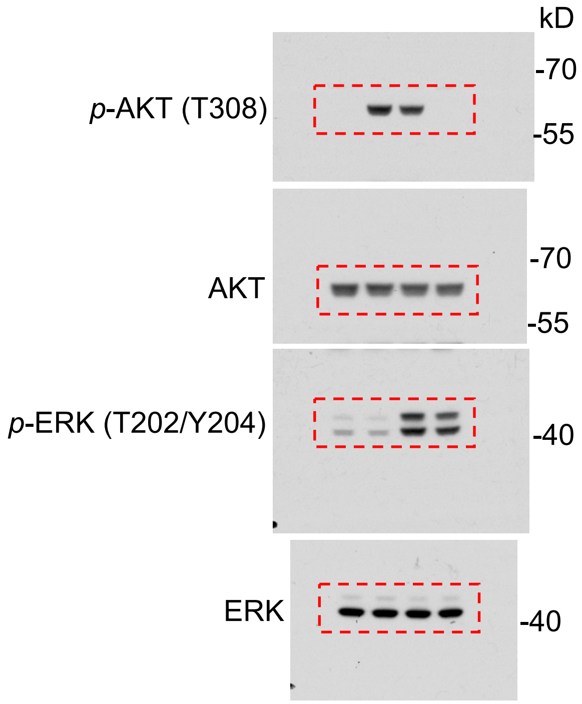

Related to Extended Data Fig. 3a

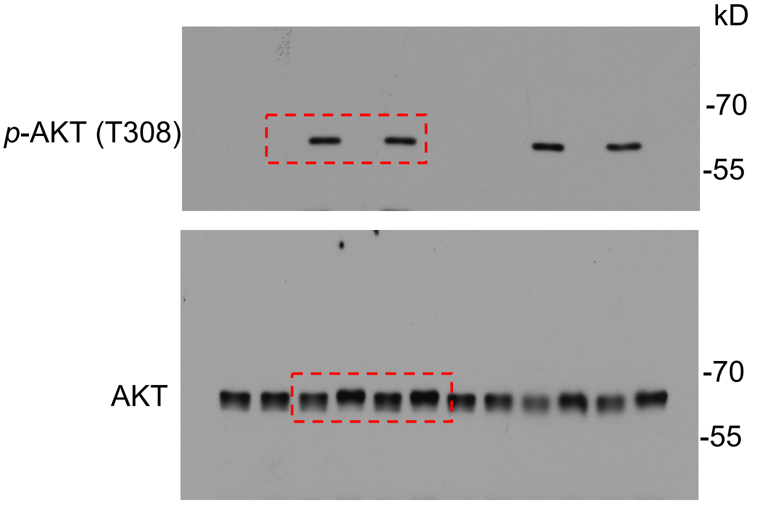

Related to Extended Data Fig. 3d

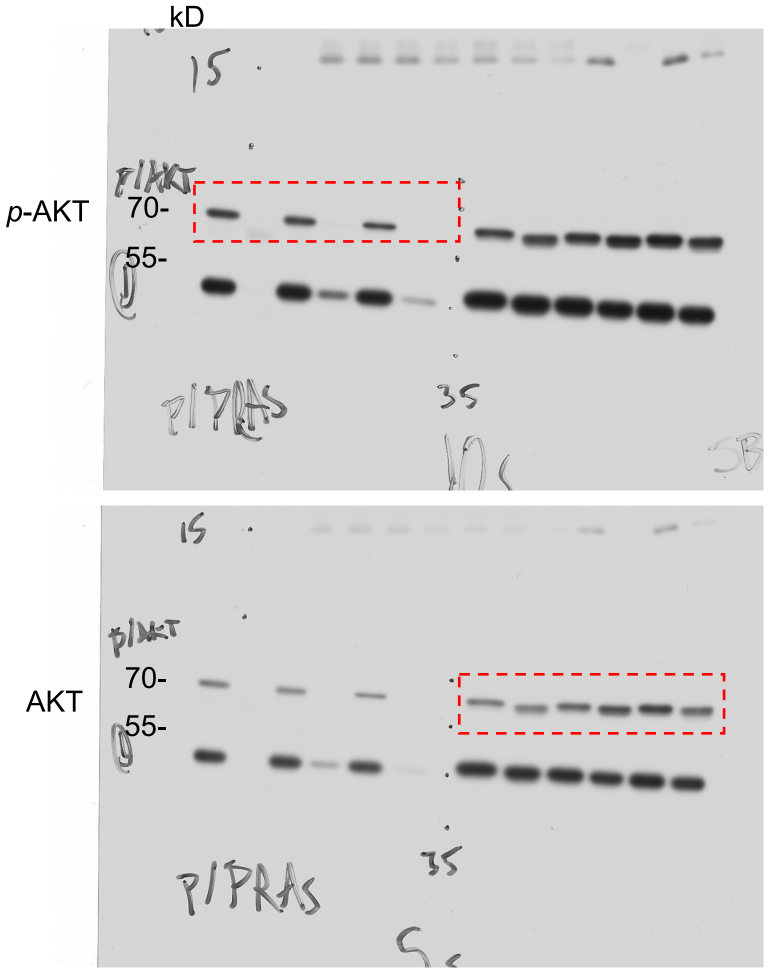

Related to Extended Data Fig. 3e

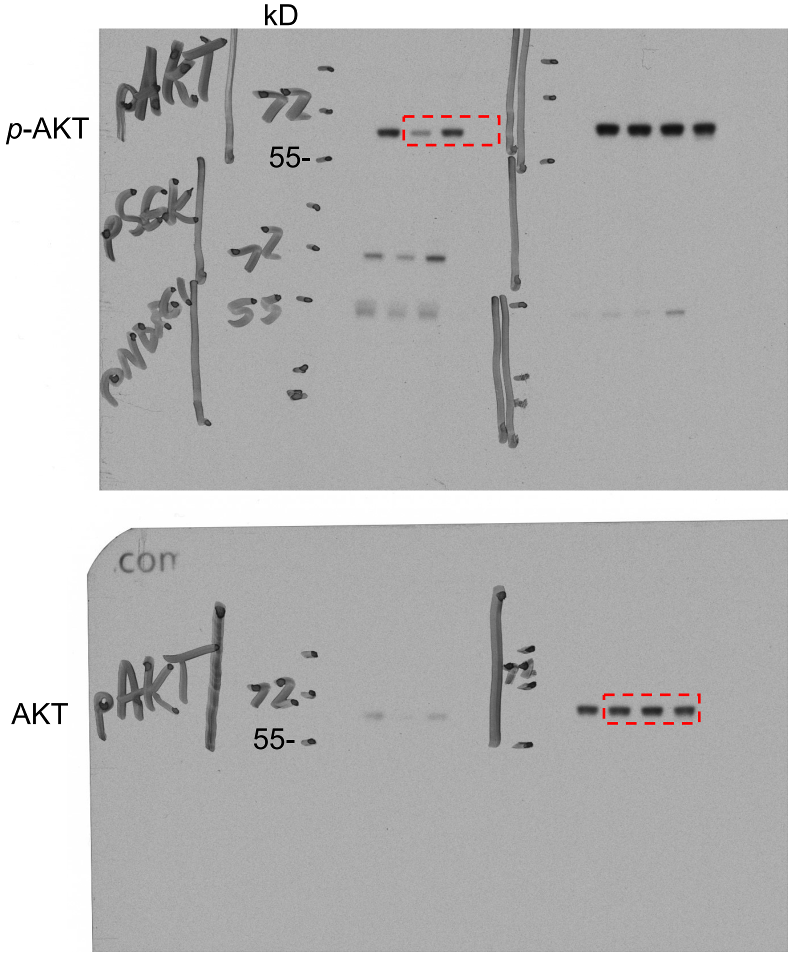

Related to Extended Data Fig. 4a

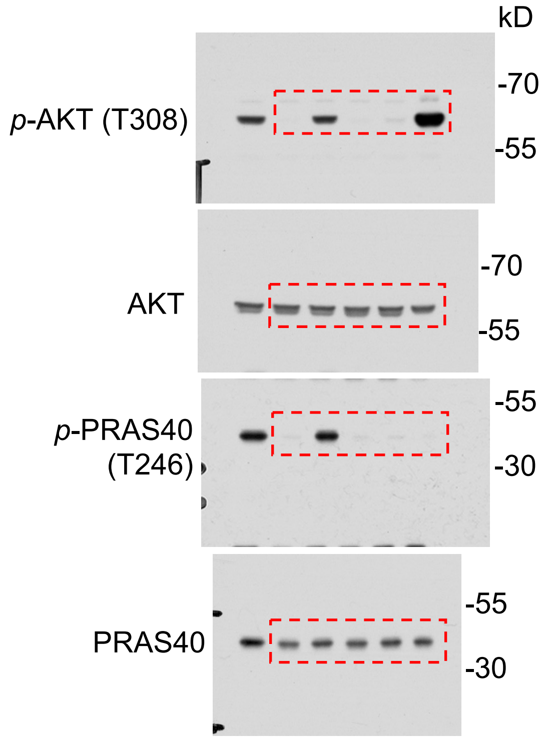

Related to Extended Data Fig. 5a

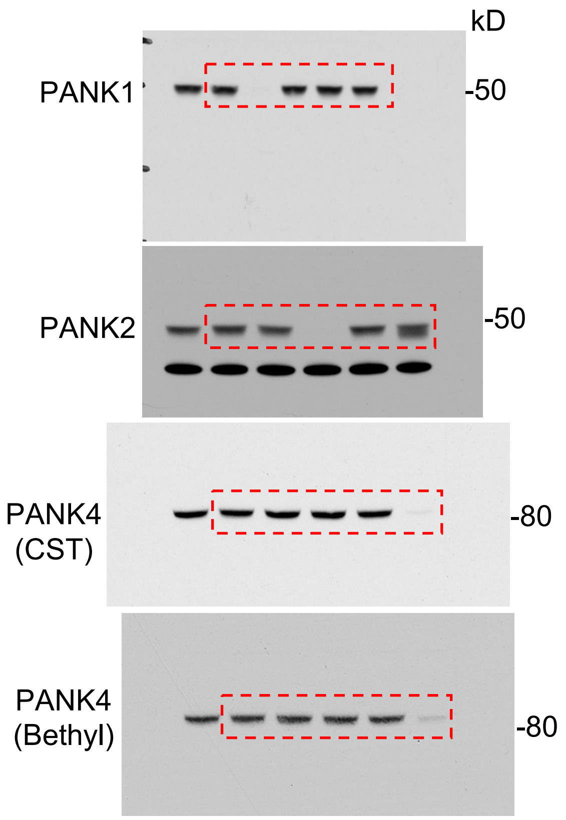

Related to Extended Data Fig. 5b

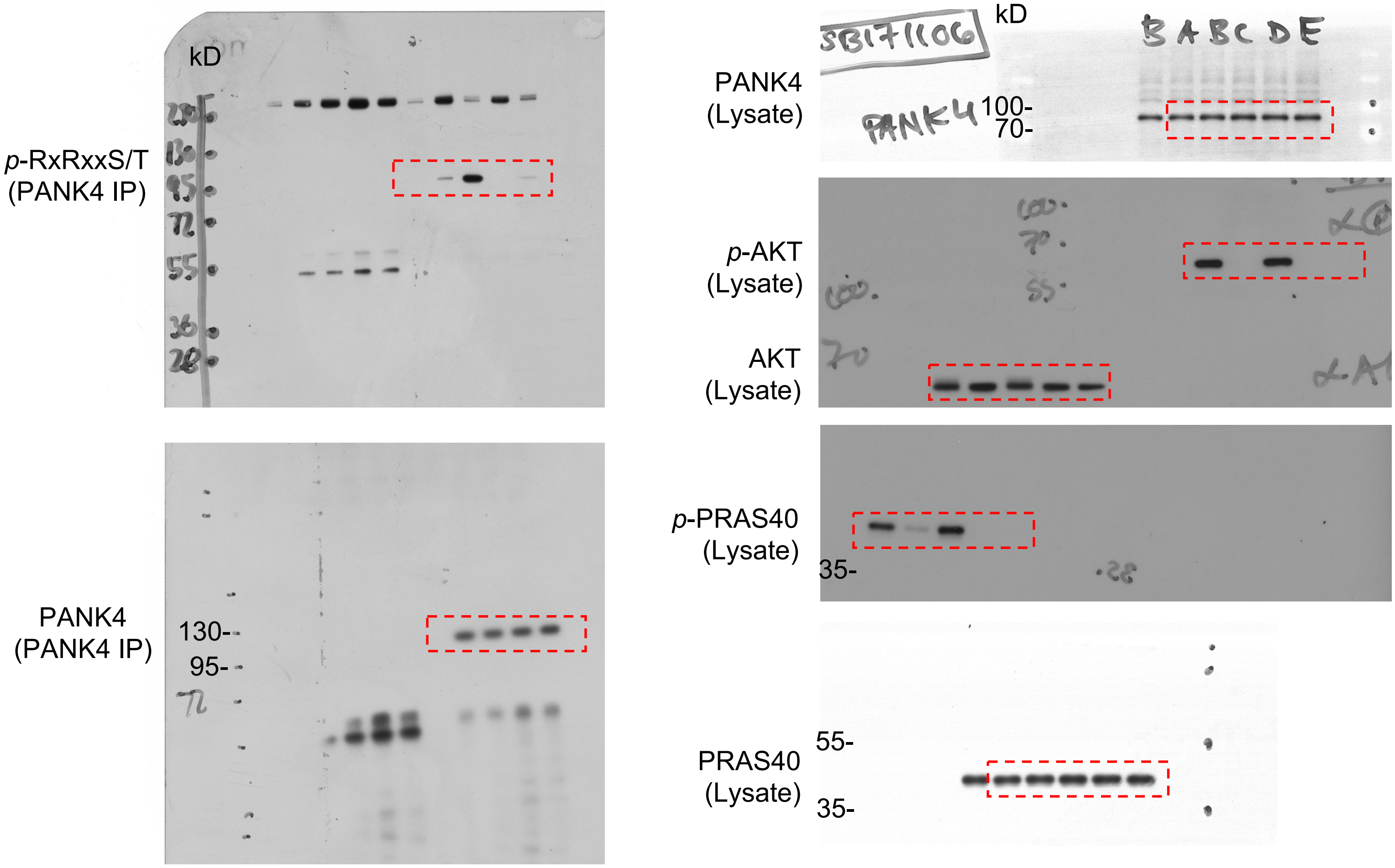

Related to Extended Data Fig. 5c

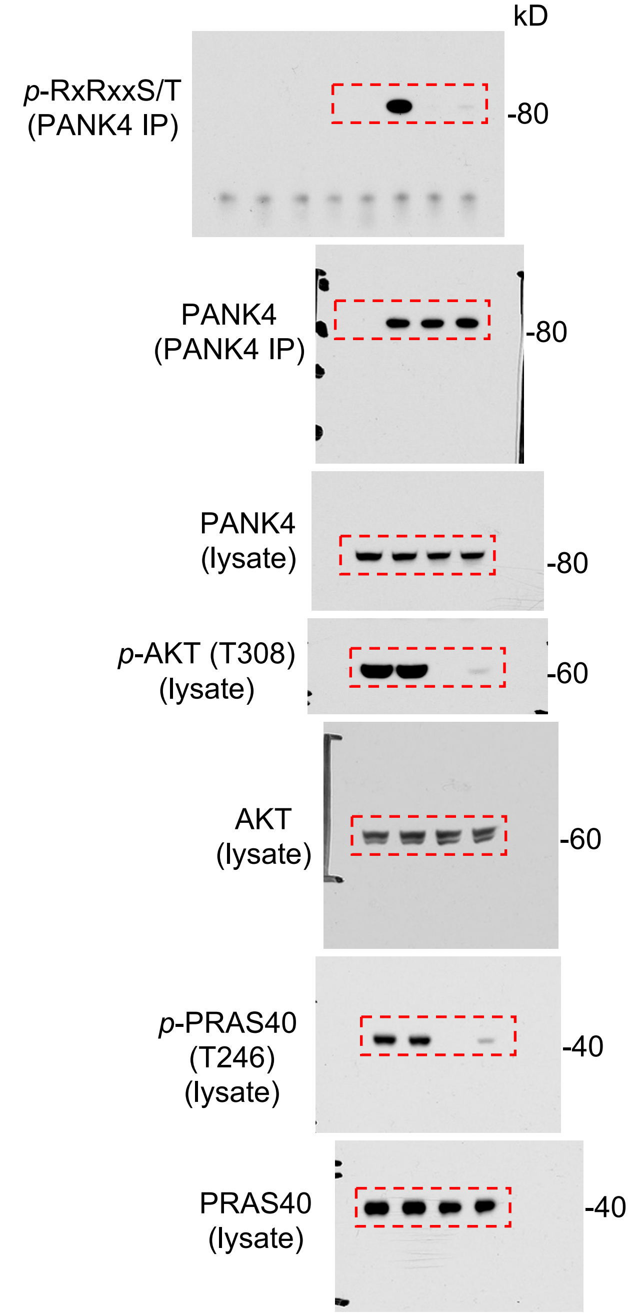

Related to Extended Data Fig. 5e continued

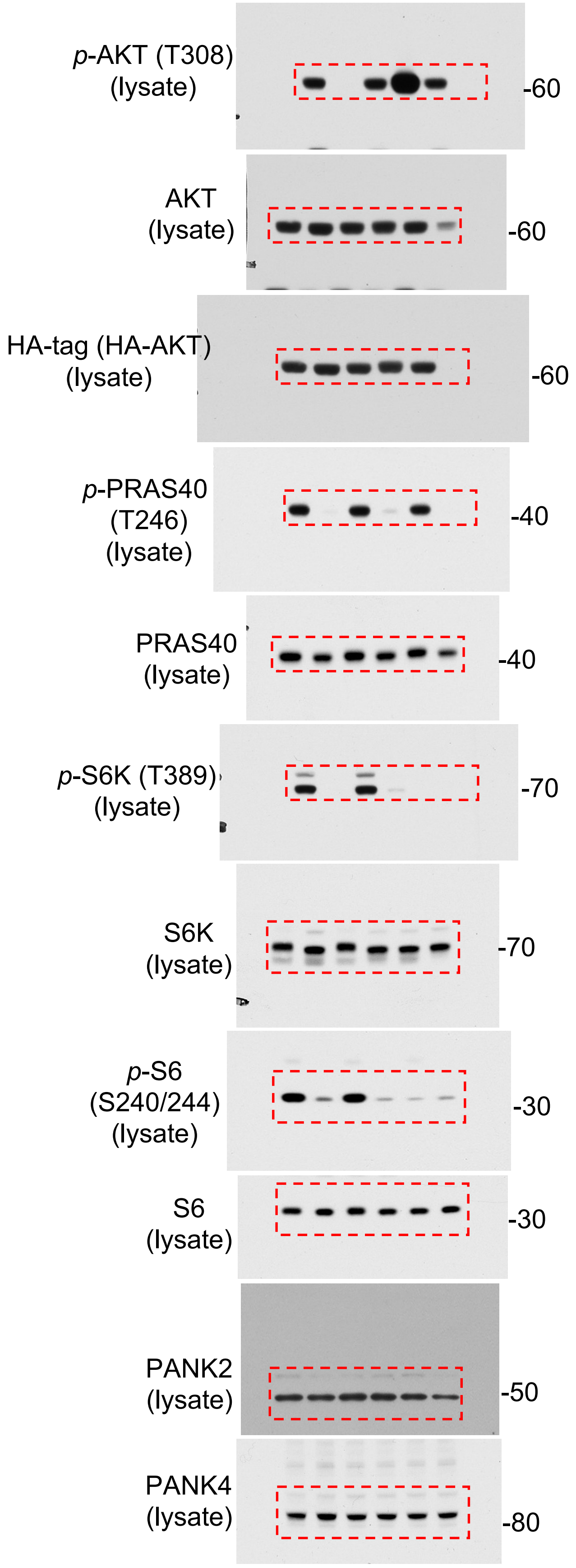

Related to Extended Data Fig. 5e

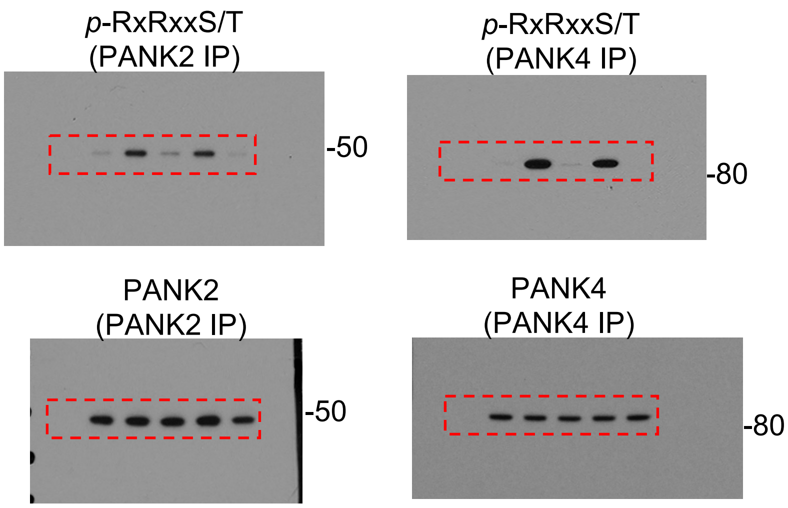

Related to Extended Data Fig. 7c

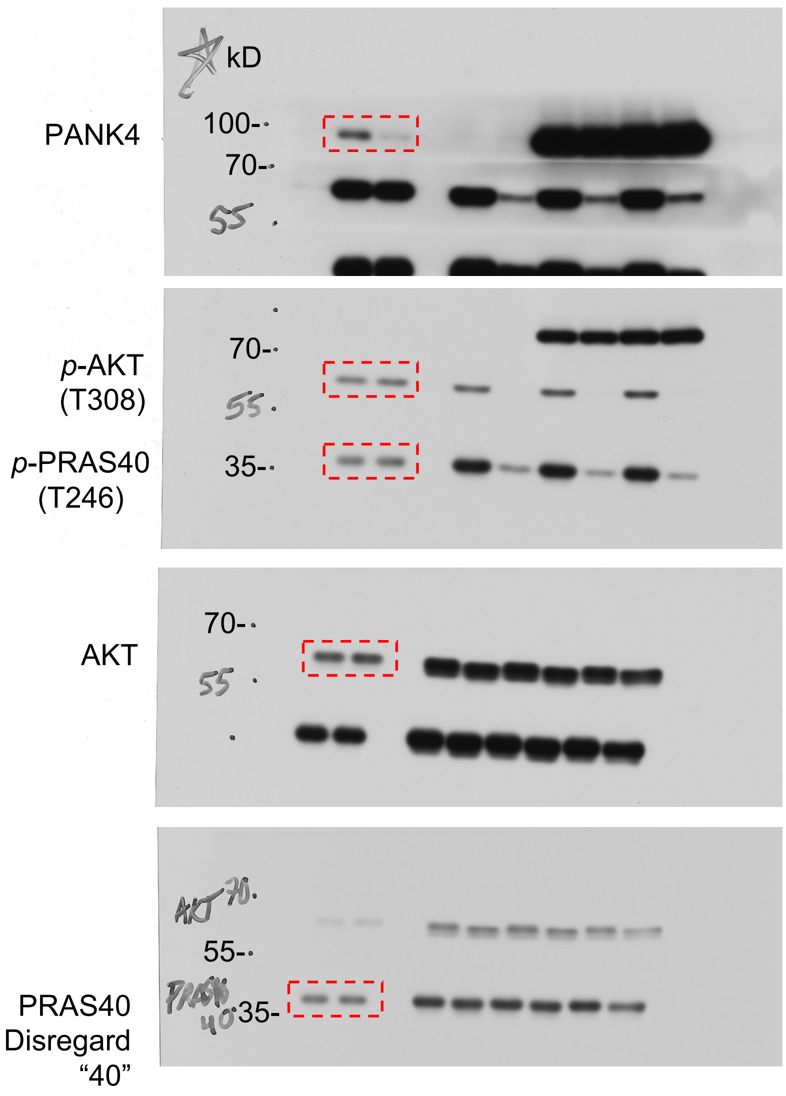

Related to Extended Data Fig. 7d

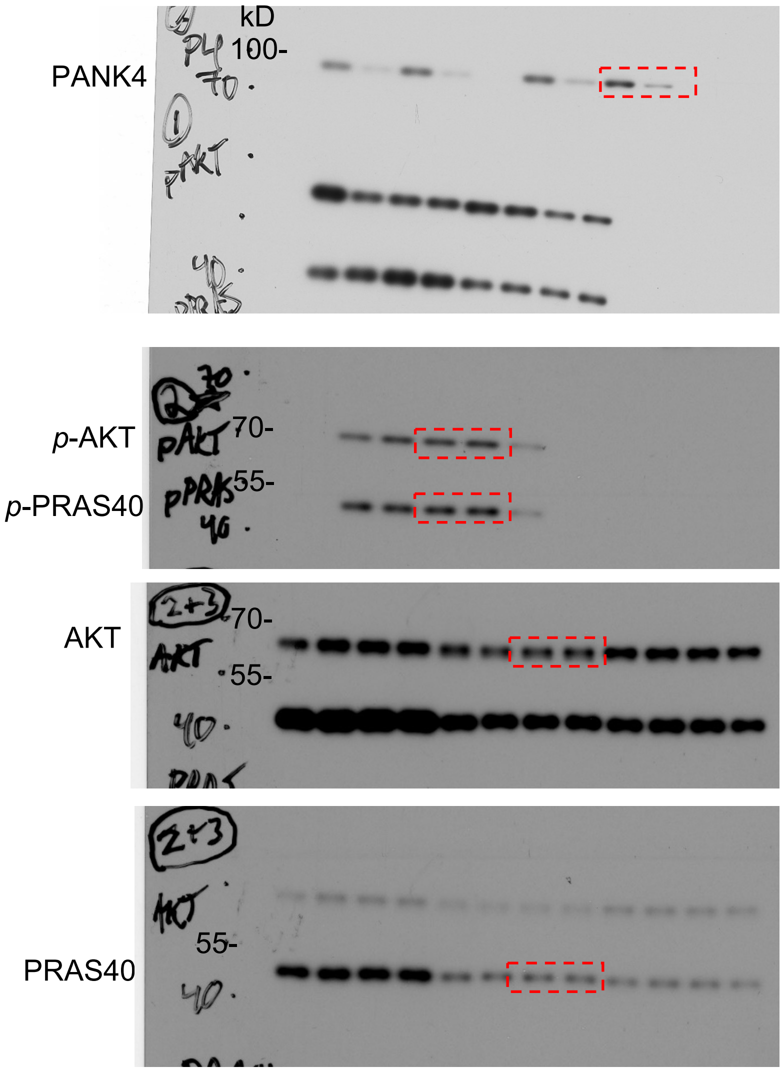

Related to Extended Data Fig. 8a

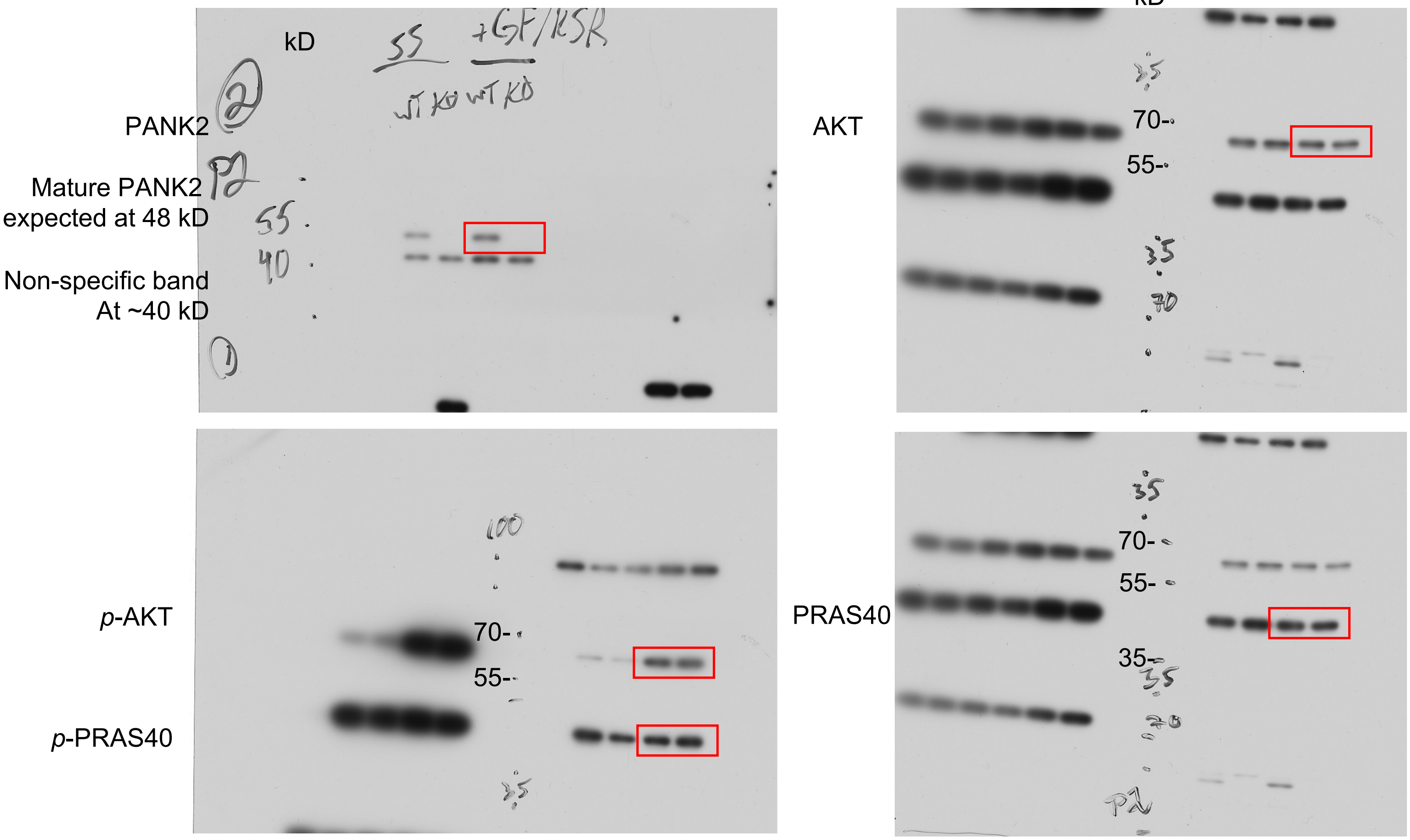

Related to Extended Data Fig. 8b

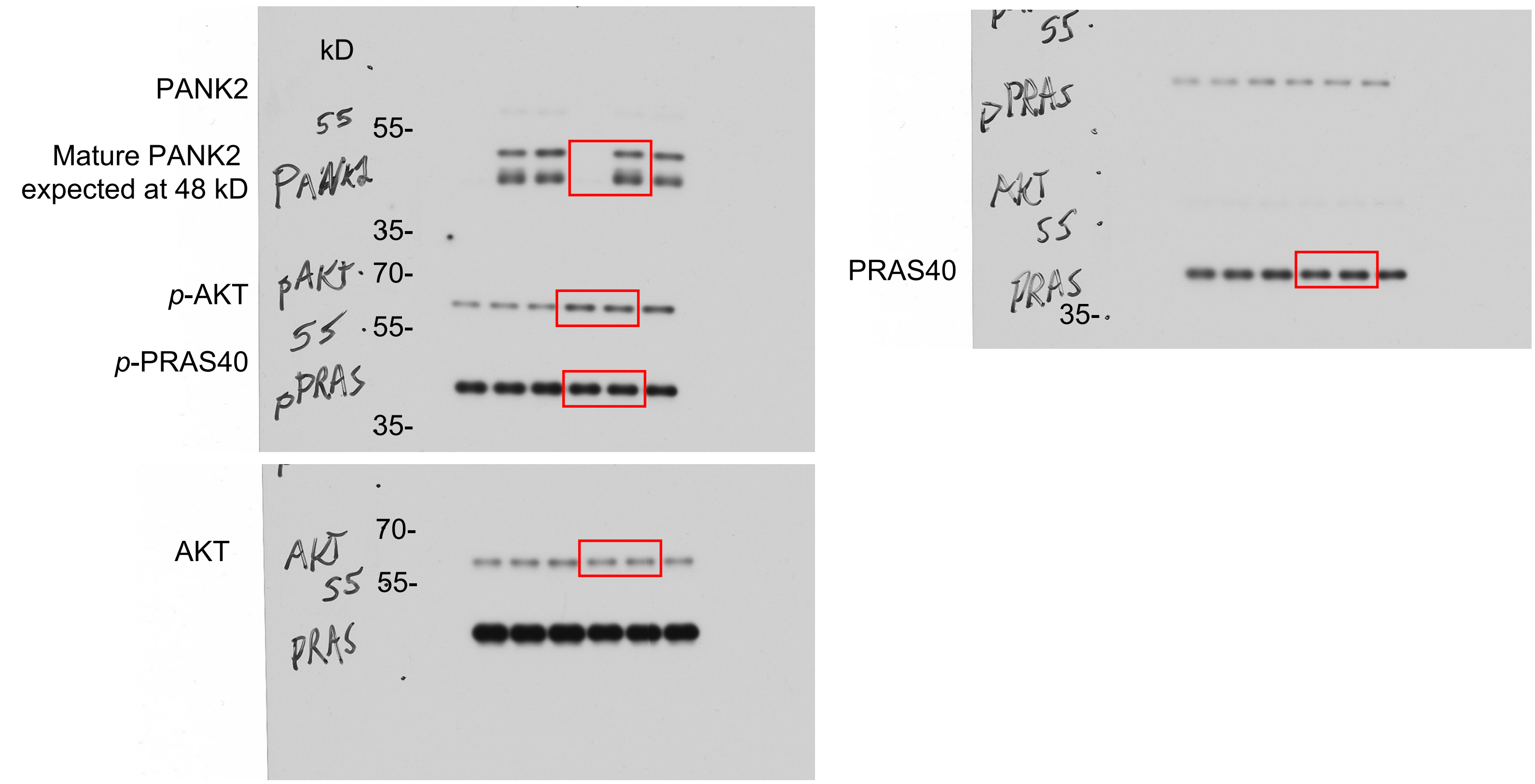

Related to Extended Data Fig. 10b

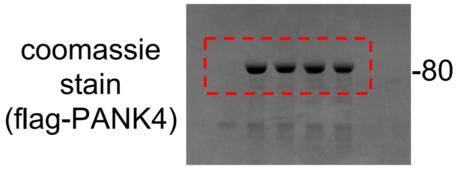

Related to Extended Data Fig. 10d

Related to Figure 10D

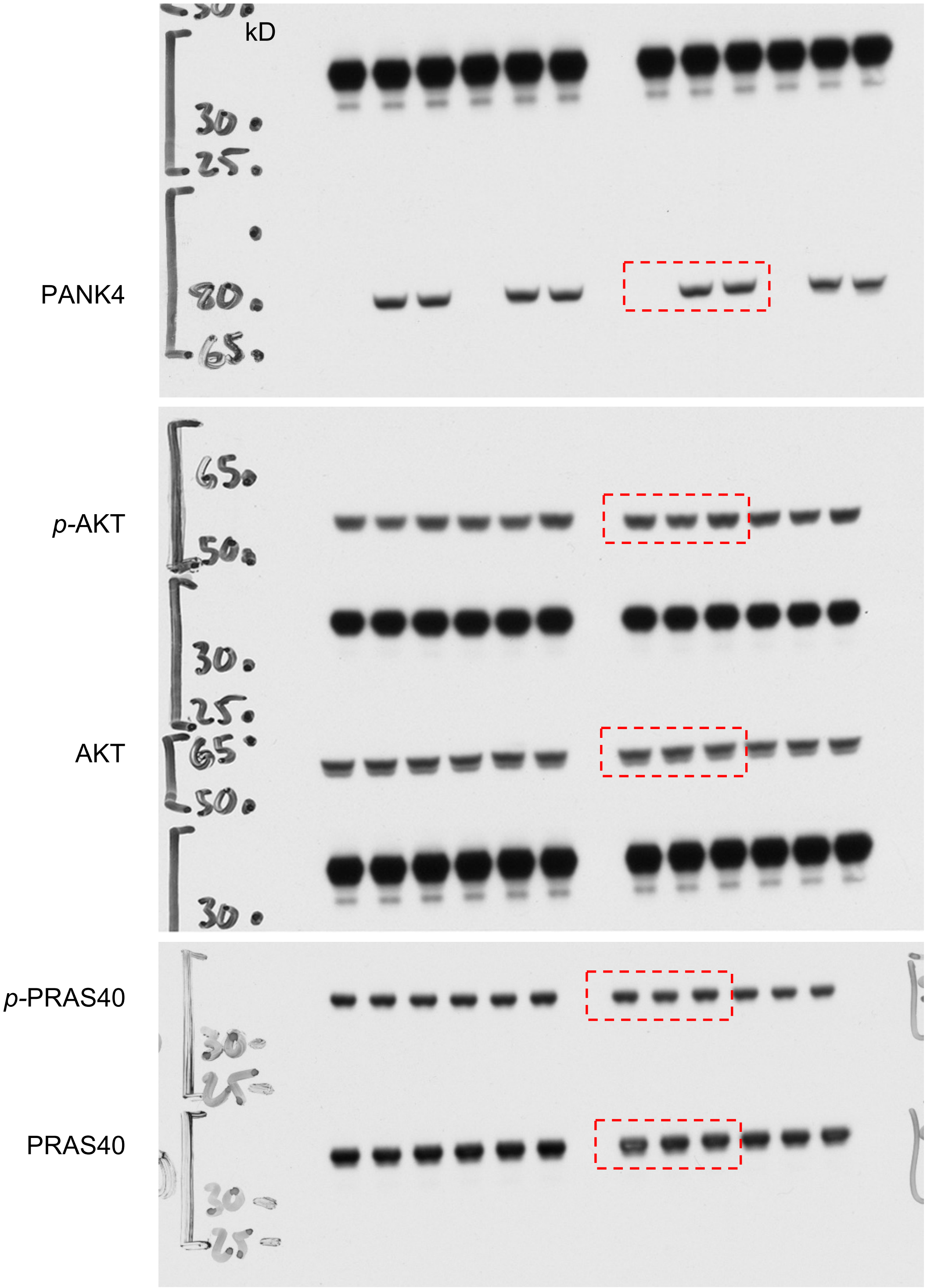

Related to Extended Data Fig. 10e

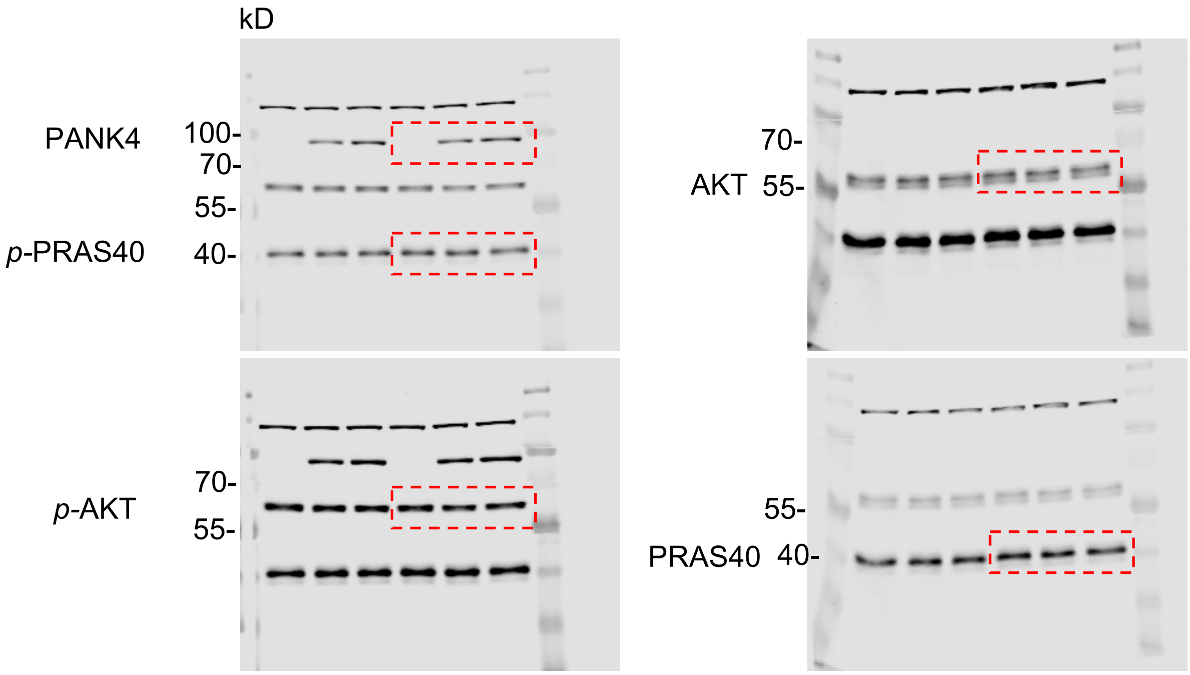

Related to Extended Data Fig. 10f

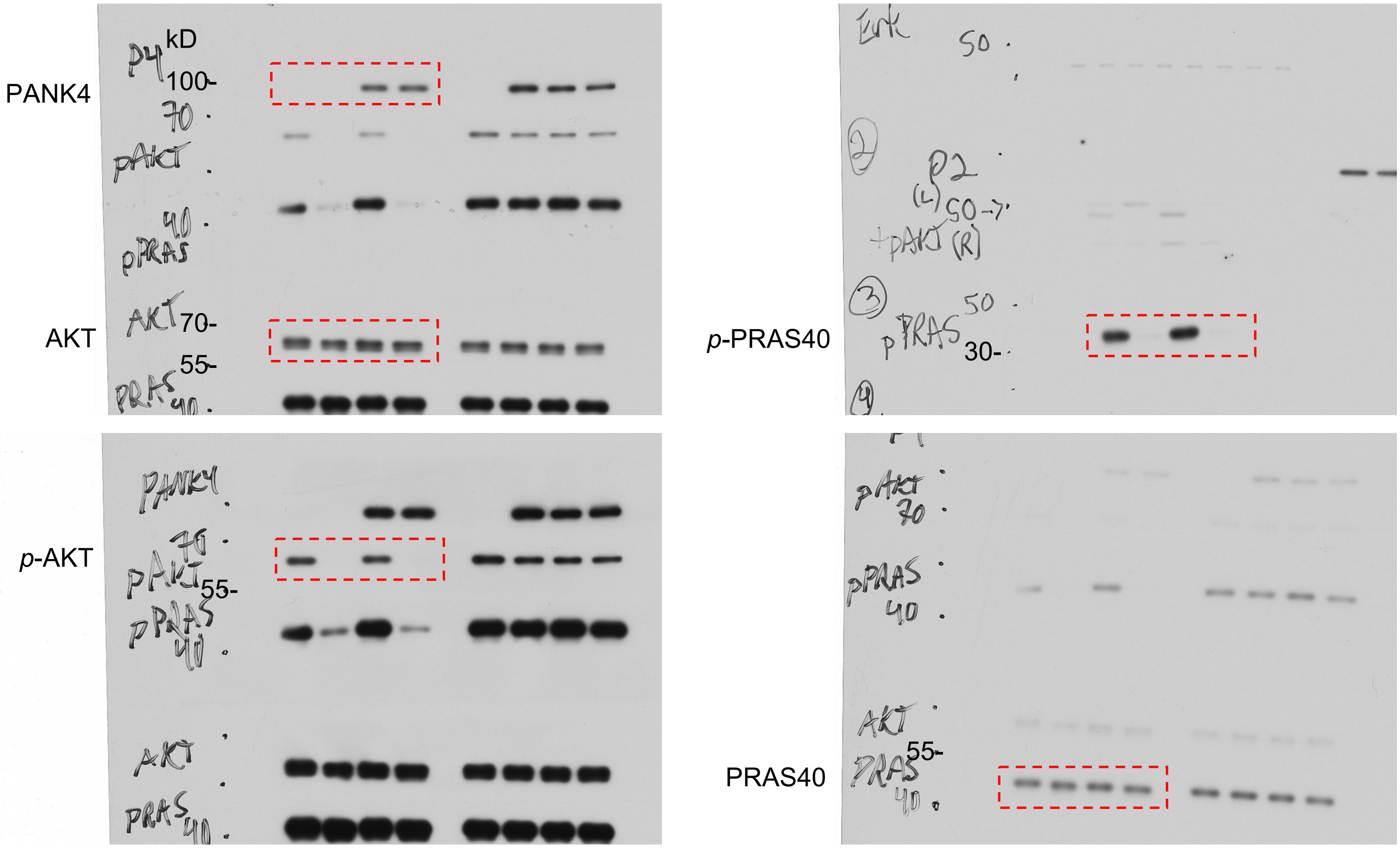

Related to Extended Data Fig. 10g

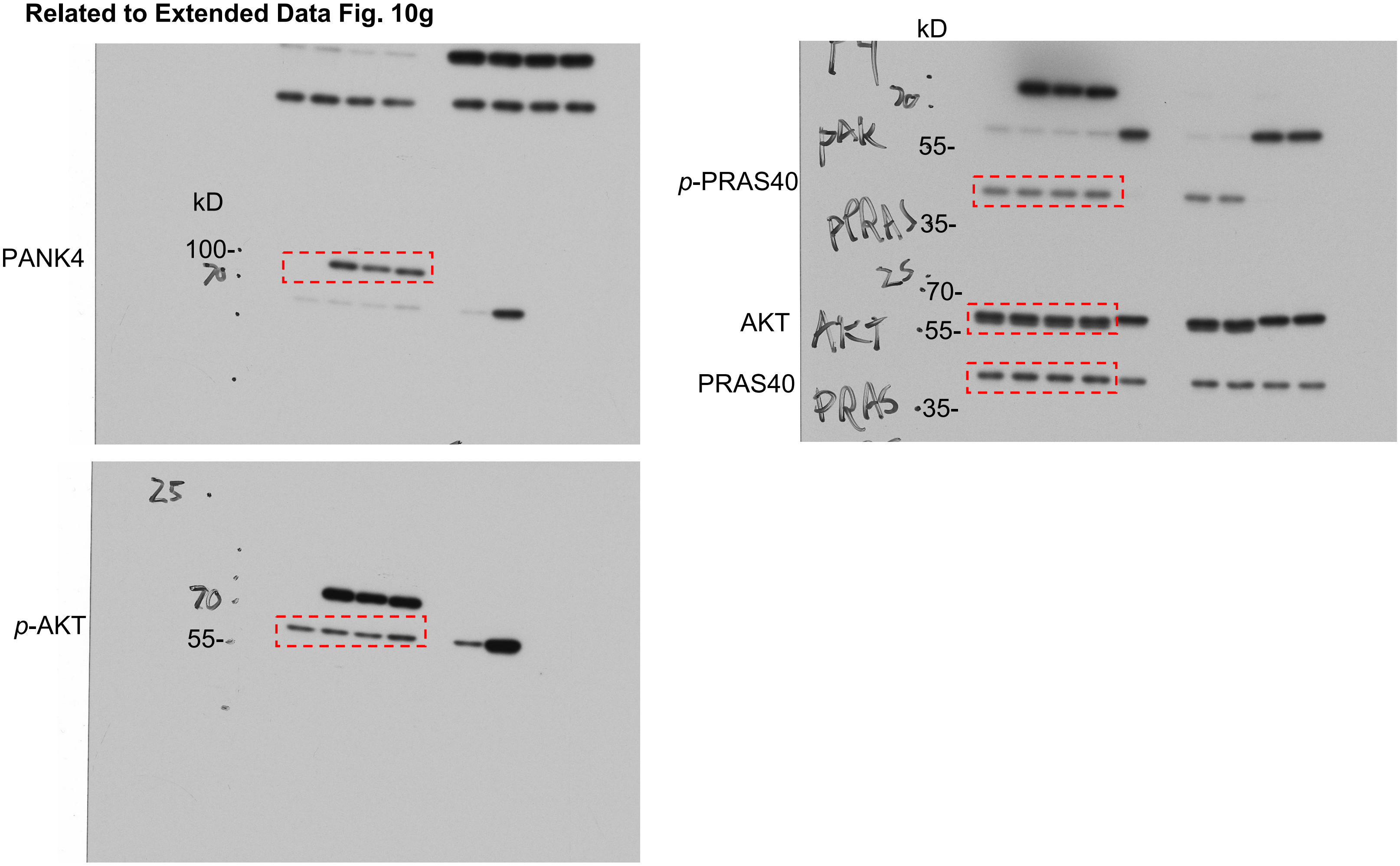

Related to Extended Data Fig. 10h

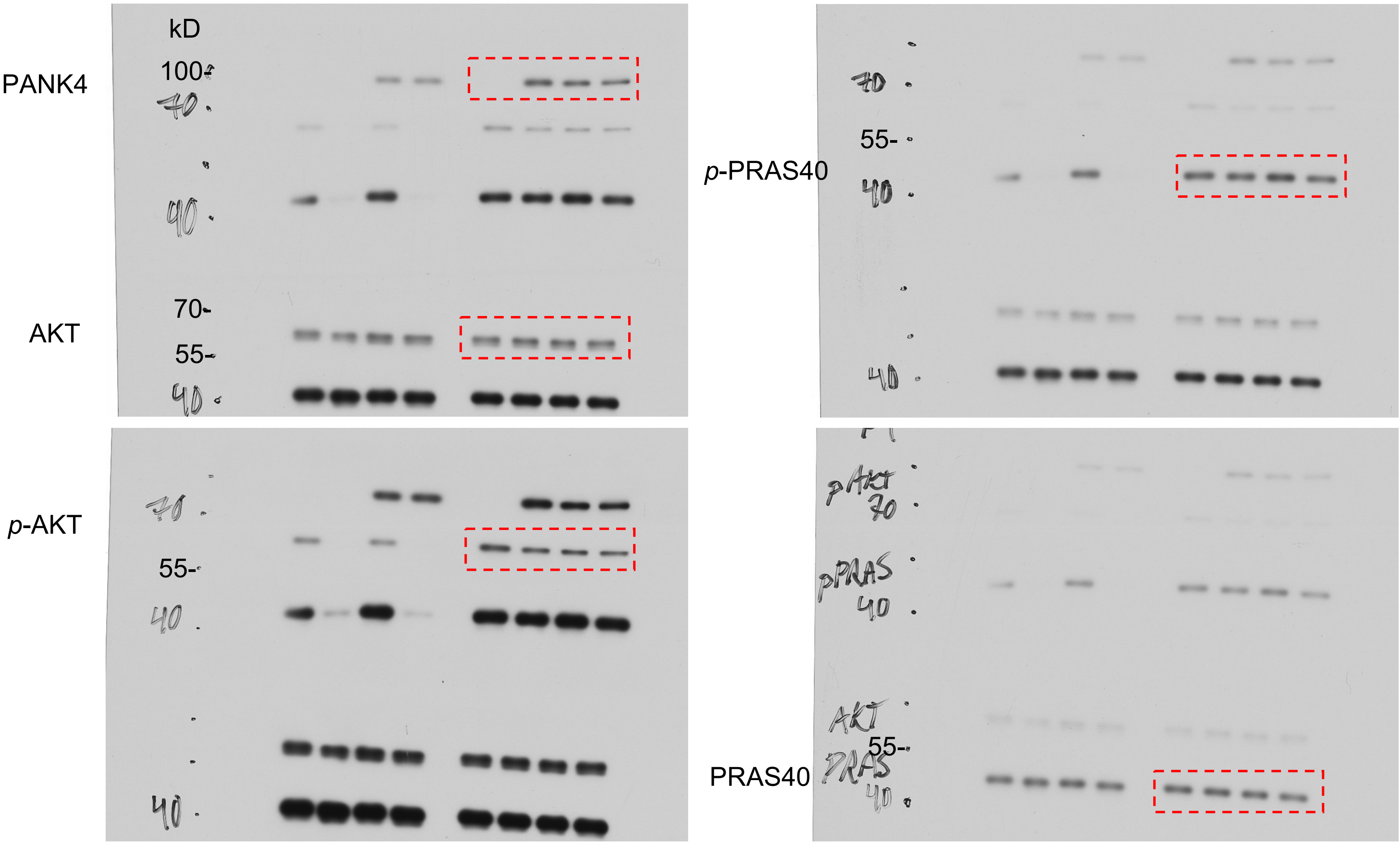

Related to Extended Data Fig. 10i

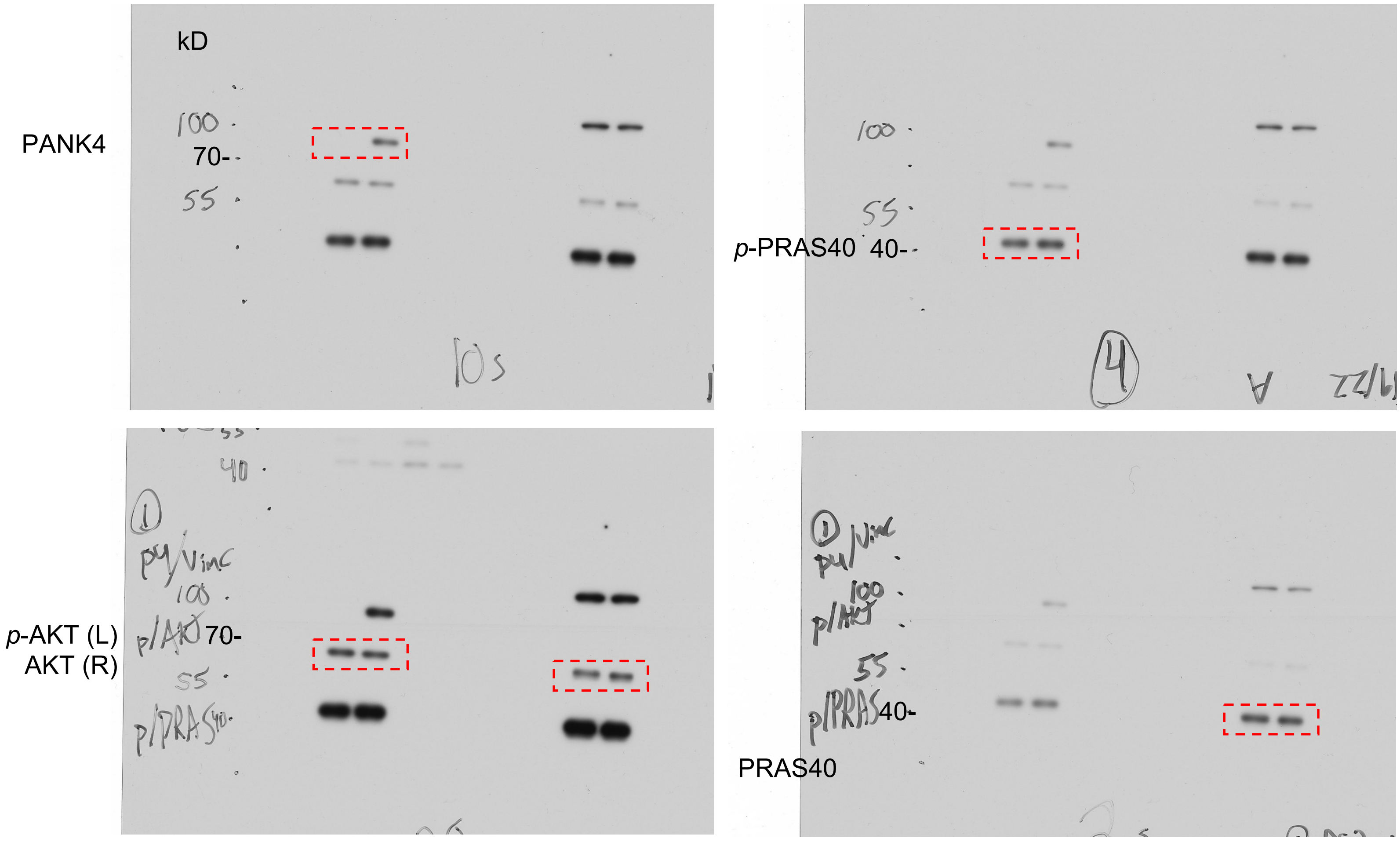

Related to Extended Data Fig. 10k

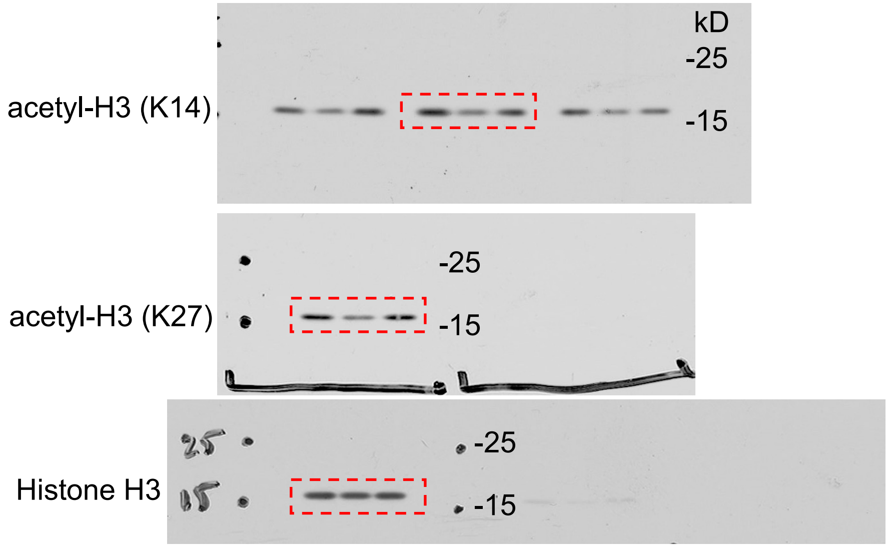

Supplement: Supplementary file 1 — Raw western blot film scans from all main and extended data figures. [file 41586_2022_4984_MOESM1_ESM.pdf]
