# Supplementary material for: PI3K drives the de novo synthesis of coenzyme A from vitamin B5
Source: Nature. 2022 Jul 27;608(7921):192–8. doi: 10.1038/s41586-022-04984-8 (PMC9352595; doi:10.1038/s41586-022-04984-8)
Supplement: Supplementary file 2 — Reporting Summary [file 41586_2022_4984_MOESM2_ESM.pdf]

nature research

Corresponding author(s): Alex Toker and Christian C. Dibble

Last updated by author(s): Mar 4, 2022

Reporting Summary

Nature Research wishes to improve the reproducibility of the work that we publish. This form provides structure for consistency and transparency in reporting. For further information on Nature Research policies, see our Editorial Policies and the Editorial Policy Checklist.

Statistics

For all statistical analyses, confirm that the following items are present in the figure legend, table legend, main text, or Methods section.

n/a	Confirmed	
☐	☒	The exact sample size (*n*) for each experimental group/condition, given as a discrete number and unit of measurement
☐	☒	A statement on whether measurements were taken from distinct samples or whether the same sample was measured repeatedly
☐	☒	The statistical test(s) used AND whether they are one- or two-sided<br>*Only common tests should be described solely by name; describe more complex techniques in the Methods section.*
☒	☐	A description of all covariates tested
☐	☒	A description of any assumptions or corrections, such as tests of normality and adjustment for multiple comparisons
☐	☒	A full description of the statistical parameters including central tendency (e.g. means) or other basic estimates (e.g. regression coefficient) AND variation (e.g. standard deviation) or associated estimates of uncertainty (e.g. confidence intervals)
☐	☒	For null hypothesis testing, the test statistic (e.g. *F*, *t*, *r*) with confidence intervals, effect sizes, degrees of freedom and *P* value noted<br>*Give P values as exact values whenever suitable.*
☒	☐	For Bayesian analysis, information on the choice of priors and Markov chain Monte Carlo settings
☒	☐	For hierarchical and complex designs, identification of the appropriate level for tests and full reporting of outcomes
☒	☐	Estimates of effect sizes (e.g. Cohen's *d*, Pearson's *r*), indicating how they were calculated

Our web collection on statistics for biologists contains articles on many of the points above.

Software and code

Policy information about availability of computer code

Data collection	MultiQuant v3.0 software (AB/SCIEX) for metabolomics data and LipidSearch 4.1.30 software (Thermo Fisher Scientific) and Elements 2.0 software for lipidomics data
Data analysis	PRISM 8 graphing software (Graphpad); ImageJ 1.50i (Wayne Rasband, NIH); MetaboAnalyst 4.0; Microsoft Excel 16.46 (Microsoft); Snapgene 5.1 (Snapgene)

For manuscripts utilizing custom algorithms or software that are central to the research but not yet described in published literature, software must be made available to editors and reviewers. We strongly encourage code deposition in a community repository (e.g. GitHub). See the Nature Research guidelines for submitting code & software for further information.

Data

Policy information about availability of data

All manuscripts must include a data availability statement. This statement should provide the following information, where applicable:

- Accession codes, unique identifiers, or web links for publicly available datasets
- A list of figures that have associated raw data
- A description of any restrictions on data availability

All data available in the main text and figures or in extended data. This study used the following public databases:
Human Metabolome Database: https://hmdb.ca/
Phosphosite Plus: https://www.phosphosite.org/homeAction
Scansite 4.0: https://scansite4.mit.edu/#home

Field-specific reporting

Please select the one below that is the best fit for your research. If you are not sure, read the appropriate sections before making your selection.

☒ Life sciences ☐ Behavioural & social sciences ☐ Ecological, evolutionary & environmental sciences

For a reference copy of the document with all sections, see nature.com/documents/nr-reporting-summary-flat.pdf

Life sciences study design

All studies must disclose on these points even when the disclosure is negative.

Sample size	No calculation was performed to determine sample size. Sample sizes for metabolomics and lipidomics experiments were n=3 per treatment were sufficient for statistical analysis of changes in metabolite and lipid levels, as well as cell growth. A sample size of n=3 was also used due to practical considerations associated with maintaining sample quality and reproducibility. A sample size of n=6 was necessary for statistical analysis of 3D soft agar assays due to intrinsically higher variability in this method.
Data exclusions	No data was excluded from the analyses
Replication	All experiments shown were replicated in full at least once (two times total) with similar results and some key experiments were replicated twice (three times total). Key results were also replicated in distinct cell types.
Randomization	Randomization was not relevant to cell line allocation to experimental groups, due to genetic homogeneity. Initial assignment of mice to treatment groups was randomized.
Blinding	Blinding was not relevant to cell culture studies due to objective measurements of metabolite levels via mass spectrometry. However, two different individuals separately reproduced each others results for key experiments. For mouse studies, researchers were blinded to treatment group during data collection (mouse/tumor assessment and measurement).

Reporting for specific materials, systems and methods

We require information from authors about some types of materials, experimental systems and methods used in many studies. Here, indicate whether each material, system or method listed is relevant to your study. If you are not sure if a list item applies to your research, read the appropriate section before selecting a response.

Materials & experimental systems

n/a	Involved in the study	
☐	☒	Antibodies
☐	☒	Eukaryotic cell lines
☒	☐	Palaeontology and archaeology
☐	☒	Animals and other organisms
☒	☐	Human research participants
☒	☐	Clinical data
☒	☐	Dual use research of concern

Methods

n/a	Involved in the study	
☒	☐	ChIP-seq
☒	☐	Flow cytometry
☒	☐	MRI-based neuroimaging

Antibodies

Antibodies used

pAKT T308 (CST 2965),
pan AKT (CST 4691),
pPRAS40 T246 (CST 2997),
PRAS40 (CST 2691),
pS6 S240/244 (CST 5364),
S6 (CST 2217),
pS6K T389 (CST 9234),
S6K (CST 2708),
p44/42 MAPK (Erk1/2) (CST 4370),
p44/42 MAPK (ERK1/2) (CST 4695)
p-ACLY S455 (CST 4331)
ACLY (CST 13390)
PANK1 (CST 23887),
PANK2 (Origene TA501321, Origene TA501355),
PANK4 (CST 12055, CST 12665),
HA Tag (CST 3724),
Flag Tag (Millipore-Sigma F7425)
pRxRxxS/T (CST 10001),

Histone H3 (CST 4499)
Acetylated-H3 (K14) (CST 7627)
Acetylated-H3 (K27) (CST 8173)
Rabbit IgG Control IP Antibody (CST 3900),
Mouse IgG Control IP Antibody (CST 5415),
Goat Anti-Rabbit IgG Antibody (H+L) HRP conjugate (Sigma-Aldrich AP307P),
Goat Anti-Mouse IgG Antibody (H+L) HRP conjugate (Sigma-Aldrich AP308P),
Anti-Rabbit Conformation Specific (CST 5127),
Rabbit Anti-Mouse IgG (Light Chain Specific) (D3V2A) mAb (HRP Conjugate) (CST 58802)

Validation

Commercially available PANK1, PANK2, and PANK4 antibodies were validated for western blot detection of each respective protein through siRNA knockdowns in this study.
Antibodies towards phosphorylated AKT, PRAS40, S6K, S6, MAPK, RxRxxS/T, and ACLY were validated by specific pathway activation via growth factor stimulation and genetic manipulation, as well as specific small molecule inhibitor treatment in this study.
The following antibodies were previously validated according to the manufacturer:
AKT: recombinant AKT and cell lysates (HeLa, NIH/3T3, C6, COS)
PRAS40: cell lysates (MCF7, HeLa, A204, RD, NIH/3T3, RAW, KNRK, COS7)
S6: cell lysates (HeLa, NIH/3T3, PC12, COS)
S6K: siRNA-mediated knockdown in HeLa
MAPK: siRNA-mediated knockdown in HEK293
ACLY: cell lysates (MCF7, HeLa, HepG2, mIMCD-3)
HA: transfection in HeLa
FLAG: overexpression in HEK293
Histone H3: cell lysates (HeLa, NIH/3T3, C6, COS)
H3K14ac: Trichostatin A treatment in HeLa, C2C12, COS7
H3K27ac: Trichostatin A treatment in HeLa, C2C12

Eukaryotic cell lines

Policy information about cell lines

Cell line source(s)	MCF10A (ATCC); MCF10A E17K/+ (Horizon Discovery, HD101-007); MCF10A PIK3CA/+ (Horizon Discovery HD-101-011); NIH 3T3 mouse fibroblasts (ATCC), SUM159 (Asterand Bioscience / BioIVT, SUM159PT); MDA-MB-468 (ATCC, HTB- 132); T47D (ATCC, HTB-133); HEK-293T (ATCC, CRL-11268)
Authentication	Cell lines were obtained directly from ATCC or BioIVT and not further authenticated.
Mycoplasma contamination	All cell lines tested negative for mycoplasma on a regular basis using the MycoAlert Detection Kit (Lonza, LT07-218)
Commonly misidentified lines (See ICLAC register)	No commonly misidentified cell lines were used in this study.

Animals and other organisms

Policy information about studies involving animals; ARRIVE guidelines recommended for reporting animal research

Laboratory animals	8-10 week-old female C57BL/6J mice; 11-12 week-old male C57BL/6J mice; 6-8 week-old NCr nude female mice
Wild animals	No wild animals were used in this study.
Field-collected samples	No field-collected samples were used in this study.
Ethics oversight	All animal experiments carried out at Beth Israel Deaconess Medical Center (BIDMC) were approved by and performed in accordance with the guidelines of the BIDMC Institutional Animal Care and Use Committee (IACUC) (animal protocol #102–2015). ES272 allograft tumor studies were approved by and performed in accordance with the guidelines of the Weill Cornell Medicine IACUC (animal protocol #2013-0116).

Note that full information on the approval of the study protocol must also be provided in the manuscript.
