# Supplementary figures and images for: PI3K drives the de novo synthesis of coenzyme A from vitamin B5
Source: Nature. 2022 Jul 27;608(7921):192–8. doi: 10.1038/s41586-022-04984-8 (PMC9352595; doi:10.1038/s41586-022-04984-8)

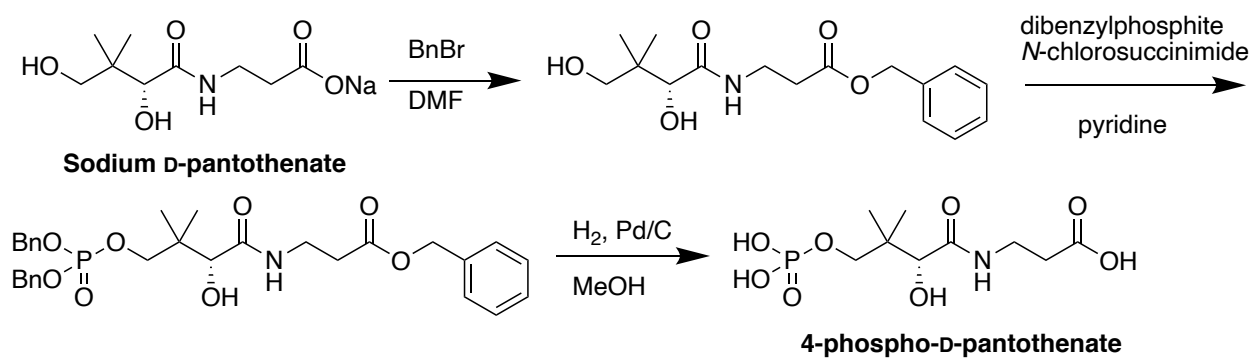

**Supplemental Information Fig. 2.** Diagram of steps for 4'-phosphopantothenate synthesis.

Supplement: Supplementary file 3 — Diagram of steps for 4′-phosphopantothenate synthesis. [file 41586_2022_4984_MOESM3_ESM.pdf]
